# Green electrochemistry-enabled one-pot synthesis of halogenated amides from readily available carboxylic acids

**DOI:** 10.1039/d6ra04649e

**Published:** 2026-07-09

**Authors:** Sudipta Ponra, Oscar Verho

**Affiliations:** a Uppsala Biomedical Centre, Department of Medicinal Chemistry, Uppsala University SE-75123 Uppsala Sweden oscar.verho@ilk.uu.se sudiptaponra@gmail.com

## Abstract

The direct use of carboxylic acids as acylating agents represents an attractive yet challenging goal in sustainable synthesis, particularly when subsequent functionalization is desired. Herein, we report a streamlined electrochemical acylation-halogenation one-pot protocol that converts readily available carboxylic acids into halogenated *N*-aryl amides *via* the *in situ* generation of reactive acyl halides. In this process, the carboxylic acid is first transformed into the corresponding acyl chloride or bromide, which subsequently undergoes amide bond formation followed by regioselective C–H halogenation under mild electrochemical conditions utilizing the released halide ion. The entire sequence proceeds in a single vessel without the need for supporting electrolyte or additional catalysts. This modular approach enables direct access to chloro- and bromo-substituted amides from simple carboxylic acid precursors, thereby providing a practical and resource-efficient alternative to conventional stepwise activation-functionalization strategies. Notably, the present method exhibits broad substrate scope and represents an innovative approach for integrating acyl activation with electrochemical late-stage C–H functionalization.

## Abstract 2

This sustainable electrochemical one-pot method enables the conversion of readily available carboxylic acids into halogenated *N*-aryl amides *via in situ* acyl halide formation, combining amidation and C–H halogenation in a single operation.

## Graphical abstract

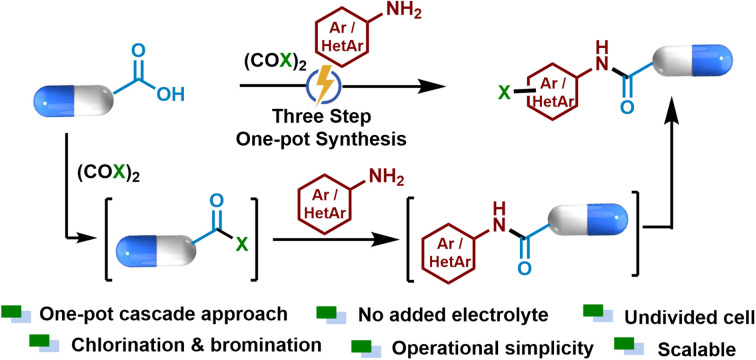

## Introduction

**Fig. 1:**
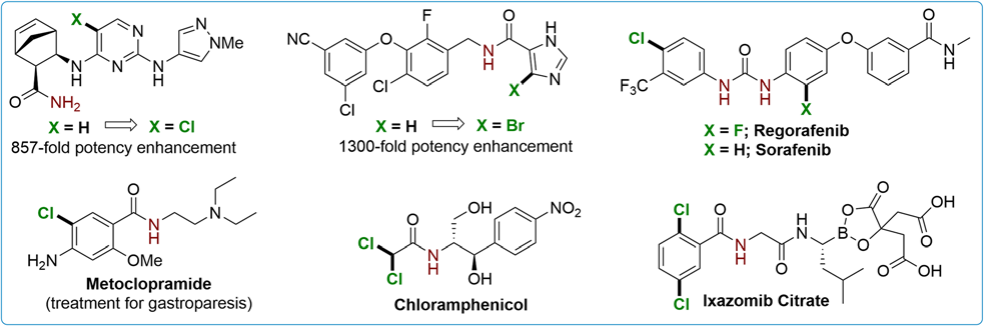
Halogenated *N*-aryl amides in bioactive molecules.

The amide moiety^1–10^ and aromatic carbon–halogen bonds^11–15^ are among the most prevalent structural motifs in organic and medicinal chemistry, underpinning the synthesis of pharmaceuticals, agrochemicals, and other value-added chemicals (Fig. 1). Consequently, methods that enable efficient amide bond formation and controlled aryl halogenation continue to attract considerable attention within the organic synthesis community. Although both transformations are individually well-established,^16–22^ they are typically performed as discrete operations, often requiring stoichiometric activating reagents, distinct reaction conditions, and intermediate purification steps. Strategies that merge amide bond formation with aryl C–H halogenation in a single operational step would therefore offer practical advantages in terms of step economy, sustainability, and overall synthetic efficiency.

Guided by the principles of green chemistry, chemists have for many years pursued the development of atom-economical, energy-efficient, and selective synthetic protocols that enable the environmentally benign production of value-added chemicals.^23–25^ Despite significant progress over the past decades, innovations in sustainable chemical processes still remain highly relevant. The chemical industry currently accounts for approximately 7% of global anthropogenic greenhouse gas emissions and it is the most energy-intense industrial sector,^26^ underscoring the need for greener and more efficient chemical processes in large-scale manufacturing.

**Scheme 1:**
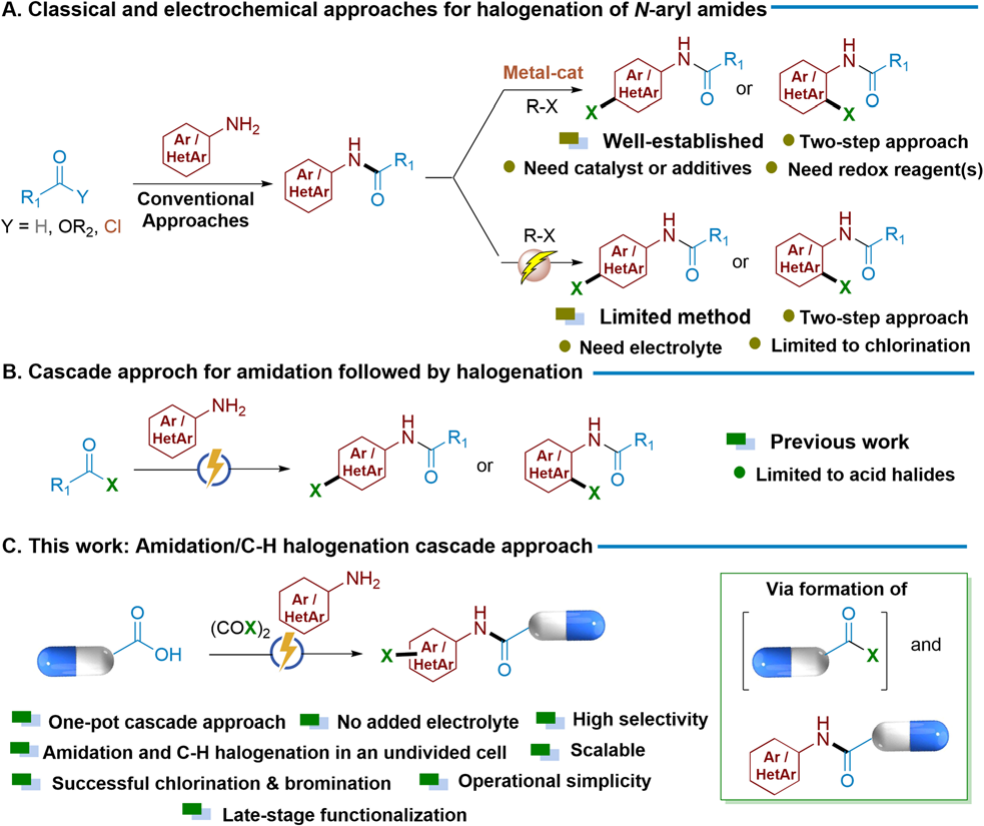
Background and reaction design. (A) Previous electrochemical methods for the halogenation of *N*-aryl amides; (B) our previous cascade approch for amidation followed by halogenation; (C) This work: a novel amidation/C–H halogenation one-pot procedure capable of transforming carboxylic acids into halogenated amide derivatives.

Cascade and one-pot methodologies provide an attractive framework for streamlining organic synthesis, as they allow sequential bond-forming events to proceed without isolation of intermediates.^27–30^ In this context, electrochemistry has emerged as a particularly powerful enabling technology with huge potential to be implemented in cascade and one-pot protocols. By using electrons as traceless redox reagents, electrochemistry enables the *in situ* generation of reactive intermediates under mild conditions, often obviating the need for external oxidants, catalysts, or stoichiometric additives.^31–48^ In particular, the anodic oxidation of halide ions provides a clean and modular entry to electrophilic halogen species capable of enabling direct and highly selective aromatic C–H halogenation (Scheme 1A). To date, a wide range of halogenating reagents have been employed for this purpose, including HX- and X_2_-type reagents,^31–38^ various metal halide salts,^39–44^ and other organic chlorine sources.^45–48^ However, despite this extensive prior art, the integration of amidation and electrochemical C–H halogenation into a unified cascade process remains surprisingly underexplored.^49,50^ In most reported cases, the halogen source, acylating agent, and redox event are introduced independently, which limits opportunities for reaction integration.

As part of our ongoing efforts to develop electrolyte-free and more sustainable halogenation strategies,^48,49^ we recently reported a mild, additive-free electrochemical cascade protocol for the efficient synthesis of halogenated *N*-aryl amides from readily available amines and acyl halides (Scheme 1B).^49^ However, this protocol was dependent on the use of acyl halides, which are generally hazardous reagents with limited commercial availability. By contrast, carboxylic acids are more practical and widely available building blocks, which prompted us to explore whether our cascade reaction could be initiated directly from carboxylic acids rather than from preformed acyl halides. To broaden the scope of our cascade methodology, we investigated whether *in situ* formation of acyl halides from carboxylic acids, followed by amidation and subsequent electrochemical C–H functionalization, could be achieved in an operationally simple and user-friendly one-pot process. This approach would directly link amidation and halogenation through a shared, widely accessible reactive intermediate, and enabling a streamlined and highly economical one-pot process.

Encouragingly, we found that acyl halide formation, amidation, and regioselective aryl halogenation can be seamlessly integrated under electrochemical conditions within a single reaction vessel, without the need for external halogenating reagents or metal catalysts (Scheme 1C). An additional advantage of this approach is that the acylating species required for amidation does not need to be introduced as an isolated reagent but can instead be generated *in situ* from the corresponding carboxylic acids, further enhancing the practicality and modularity of the method. As described herein, this electrochemical amidation-halogenation one-pot sequence provides direct access to halogenated *N*-aryl amides from simple carboxylic acid starting materials under mild reaction conditions, while maintaining broad functional group tolerance and high regioselectivity. Remarkably, the electrochemically induced C–H halogenation step proceeds with high efficiency at room temperature, without any supporting electrolyte beyond the anilinium halide salts that are transiently formed over the course of the reactions,^49^ highlighting the operational simplicity and high sustainability of this method. Notably, the robustness of this cascade enables its application to structurally complex and pharmaceutically relevant molecules, demonstrating its potential for late-stage functionalization and medicinal chemistry workflows.

## Results and discussion

Our initial efforts focused on the one-pot conversion of carboxylic acids to the corresponding acyl halides, followed by electrochemical halogenation of the *in situ* generated *N*-aryl amides. Optimization of this electrochemical one-pot protocol was carried out using cyclohexanecarboxylic acid (1) and aniline (3) as model substrates with various halogenating reagents, and was greatly inspired by our previous results.^49^ All experiments (Table 1) were conducted in a one-pot fashion in an undivided cell without any additional additives or catalysts other than the inserted anode and cathode. In the first experiment, oxalyl chloride (2) was employed as the halogenating reagent in the presence of a catalytic amount of dimethylformamide (DMF), with dichloromethane (DCM) as the solvent. After stirring for 2 h and removing the DCM, electrolysis was performed using a carbon cloth anode and a platinum plate cathode, and dimethylacetamide (DMA) as the solvent. Under these conditions, product 4 was obtained in an excellent yield of 97% (entry 1). Screening experiments conducted in the absence of a halogenating reagent revealed that such a reagent is essential for converting the carboxylic acid to the corresponding acyl chloride prior to the electrochemical amidation/halogenation cascade (entry 2). As expected, exclusive formation of amide 3a was observed in the absence of applied current (entry 3), with no chlorinated product detected, highlighting the crucial role of electrochemistry in promoting the final C–H chlorination step. Moreover, performing the reaction without removing dichloromethane, or using a 1 : 1 mixture of dichloromethane and DMA as the solvent, resulted in only 35% yield of the desired product 4 along with 15% of amide 3a (entry 4). Switching to thionyl chloride (SOCl_2_) as the halogenating reagent resulted only in the formation of the intermediate amide without any detectable amounts of chlorinated products 4 or 5 (entries 5 and 6). Furthermore, the use of hydrogen chloride (HCl) proved unfruitful under comparable conditions as well (entry 7). Interestingly, doubling the equivalents of oxalyl chloride and increasing the current to 10 mA significantly enhanced the formation of the dichlorinated product 5, which reached 47% yield (entry 8). Encouraged by the successful chlorination sequence, we investigated whether an analogous acylation/bromination variant could be achieved by replacing oxalyl chloride with oxalyl bromide. Gratifyingly, by increasing the reaction time to 6.5 h and applying a current of 15 mA, the corresponding brominated product *N*-(4-bromophenyl)acetamide could be obtained in 88% yield (entry 9). Based on these optimization studies, oxalyl halide was selected as the halogenating reagent along with a CC(+)/Pt(−) electrode setup for subsequent substrate scope investigations. The optimized conditions were as follows: 1.5 equivalents of oxalyl chloride and 5 mA current for monochlorination; 1.5 equivalents of oxalyl bromide and 15 mA current for monobromination; and 3.0 equivalents of oxalyl chloride with a 10 mA current for dichlorination.

**Table 1:** Optimization study of the electrochemical cascade approach^a^

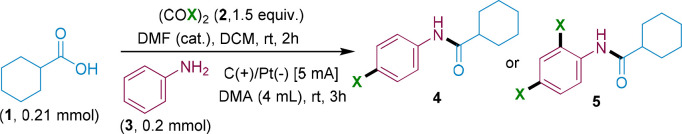				
Entry	Halogenating reagent	Modification	Charge	4 : 5
1	Oxalyl chloride	None	2.8 F	97 : 0
2	—	Without oxalyl chloride	2.8 F	0 : 0
3	Oxalyl chloride	Without current	0	0 : 0
4	Oxalyl chloride	Without removal of DCM	2.8 F	35 : 0
5	Thionyl chloride	None	2.8 F	0 : 0
6	Thionyl chloride	Without removal of DCM	2.8 F	0 : 0
7	Hydrochloric acid	None	2.8 F	0 : 0
8	Oxalyl chloride (3 equiv.)	10 mA, 40 h instead of 5 mA, 3 h	74.6 F	0 : 47
9	Oxalyl bromide	15 mA, 6.5 h instead of 5 mA, 3 h	18.2 F	88 : 0

a Reagents and conditions: first, carboxylic acid 1 (0.21 mmol), (COX)_2_ (0.315 mmol) and DMF (cat.) in DCM (2 mL, rt, 2 h, and then 2 (0.2 mmol) in DMA (4 mL), 5 mA in an undivided cell at rt, unless otherwise noted. Yields of 4 and 5 were determined by ^1^H-NMR against 1,3,5-trimethoxybenzene as the internal standard.

**Scheme 2:**
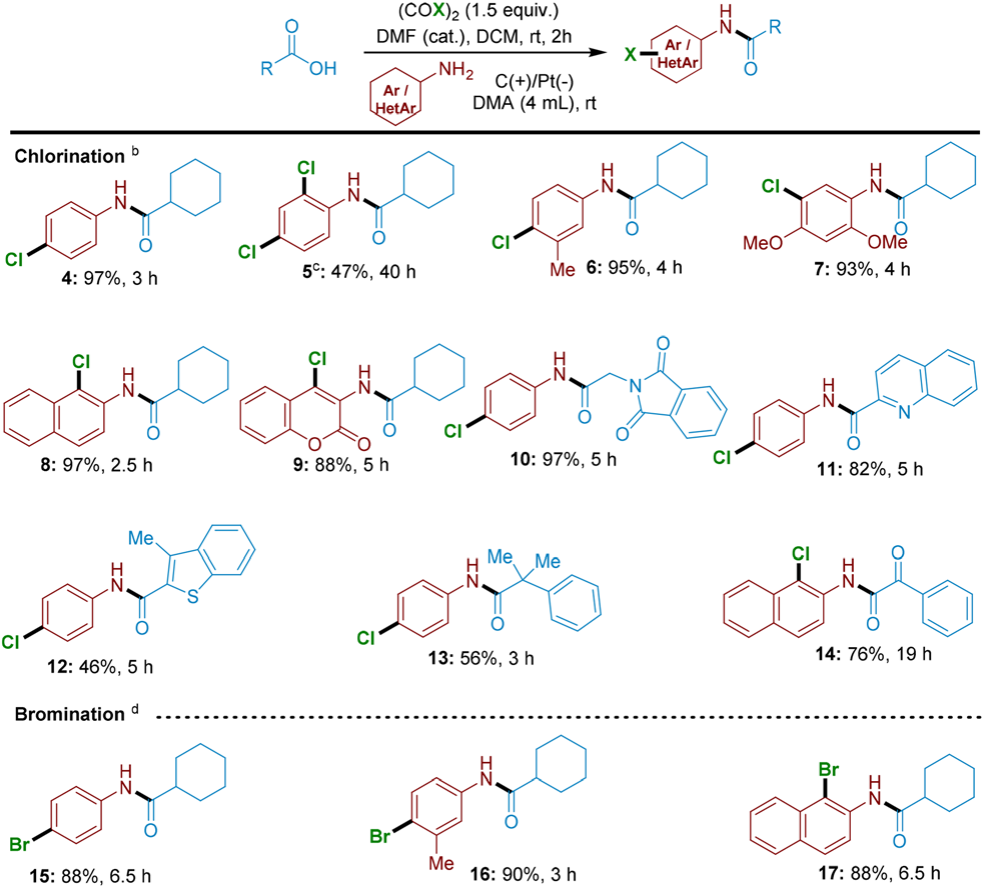
Generation of acyl halide followed by cascade amidation/halogenation^*a*^. Reagents and conditions: ^*a*^first carboxylic acid (0.21 mmol), (COX)_2_ (0.315 mmol) and DMF (cat.) in DCM (2 mL), rt, 2 h, then removal of DCM under reduced pressure followed by amine (0.2 mmol) in DMA (4 mL), in an undivided cell with carbon cloth (anode) and platinum (cathode). ^*b*^(COCl)_2_, 5 mA; ^*c*^(COCl)_2_ (0.63 mmol). ^*d*^(COBr)_2_ (2M in DCM), 15 mA. All yields refer to isolated yields.

With the optimized conditions in hand, we explored the scope of the electrochemical one-pot sequence using different amines and carboxylic acids for both chlorination and bromination (Scheme 2). First, the reactions of cyclohexanecarboxylic acid (1) with a variety of aromatic and heteroaromatic amines were examined. As demonstrated by the reactions providing products 4, 6 and 7, anilines with free *para*-positions represent highly competent reaction partners in this one-pot protocol and exclusively gave the *para*-chlorinated products in 93–97% yield. For aromatic amines with blocked *para* positions, C–H chlorination was instead found to occur in high regioselectivity at the position adjacent to the amide group. This was exemplified by the reactions of 2-naphthylamine and 3-aminocoumarin, which afforded product 8 in 97% yield within 2.5 h and product 9 in 88% yield within 5 h, respectively. To further assess the generality of this one-pot protocol, various carboxylic acids were investigated using aniline (2) as the model amine. 2-(1,3-Dioxoisoindolin-2-yl)acetic acid reacted smoothly to give product 10 in 97% yield within 5 h. Heterocyclic acids such as quinoline-2-carboxylic acid and 3-methylbenzo[*b*]thiophene-2-carboxylic acid were also competent substrates, affording products 11 and 12 in 82% and 46% yields, respectively. Sterically congested aliphatic acids, including 2-methyl-2-phenylpropanoic acid, provided product 13 in 56% yield. Notably, 2-oxo-2-phenylacetic acid also showed excellent compatibility, furnishing product 14 in 76% yield, underscoring the versatility of this cascade approach. Following the chlorination studies, we evaluated the generality of the amidation/bromination cascade using oxalyl bromide at a 15 mA current. Similar trends were observed: amines with unoccupied *para*-positions afforded the corresponding *para*-brominated products 15 and 16 in 88% and 90% yield, respectively. In the case of 2-naphthylamine, bromination occurred selectively at the position adjacent to the amide group, delivering product 17 in 88% yield.

**Scheme 3:**
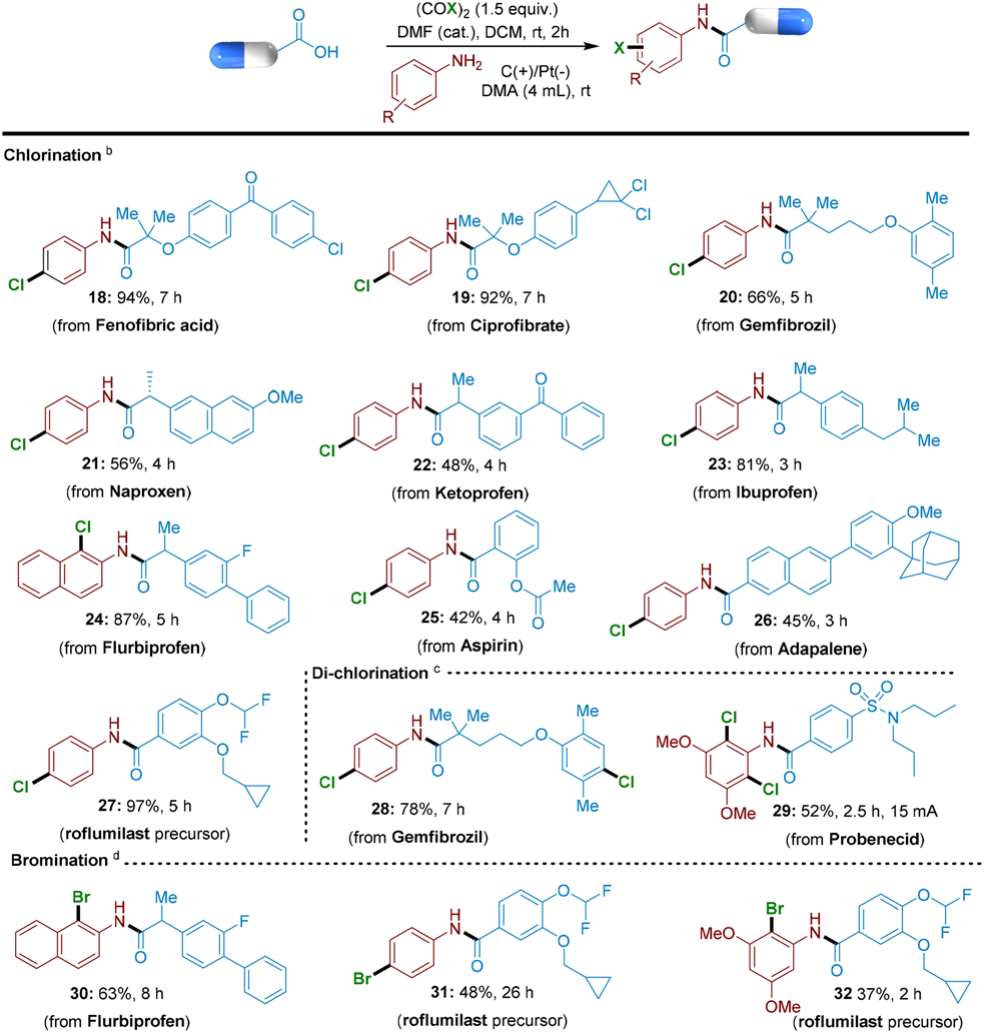
Electrochemical one-pot synthesis of halogenated bioactive molecules^*a*^. Reagents and conditions: ^*a*^first carboxylic acid reagent (0.21 mmol), (COX)_2_ (0.315 mmol) and DMF (cat.) in DCM (2 mL), rt, 2 h, then removal of DCM under reduced pressure followed by amine (0.2 mmol) in DMA (4 mL), in an undivided cell with carbon cloth (anode) and platinum (cathode). ^*b*^(COCl)_2_, 5 mA; ^*c*^carboxylic acid (0.42 mmol), (COCl)_2_ (0.63 mmol) DCM (2 mL), DMF (cat.), rt, 2 h, and amine (0.2 mmol) in DMA (4 mL), 5 mA; ^*d*^(COBr)_2_ (2 M in DCM), 15 mA. All yields refer to isolated yields.

Furthermore, we explored the potential of our method to be used for the late-stage functionalization of pharmaceutically relevant scaffolds (Scheme 3). For example, the lipid-lowering drugs Fenofibric acid, Ciprofibrate, and Gemfibrozil all proved compatible with our one-pot protocol, affording the corresponding halogenated amide analogs 18–20 in 66–94% yields. Nonsteroidal anti-inflammatory drugs including Naproxen, Ketoprofen, Ibuprofen, Flurbiprofen, and Aspirin furnished products 21–25 in yields ranging between 42–87%. Adapalene gave the chlorinated amide analog 26 in 45% yield, while a synthetic fragment of the COPD drug Roflumilast (27) could be synthesized in an excellent yield of 97%. By increasing the equivalents of carboxylic acid reaction partner and oxalyl chloride, the dichlorinated analogs 28 and 29 could be obtained in 78% amd 52% yield respectively. Mono-brominated pharmaceutical analogues were also successfully prepared using this protocol, as demonstrated by the reactions providing products 30–32 in 37–63% yield, when using a 15 mA current in the final C–H bromination step.

**Scheme 4:**
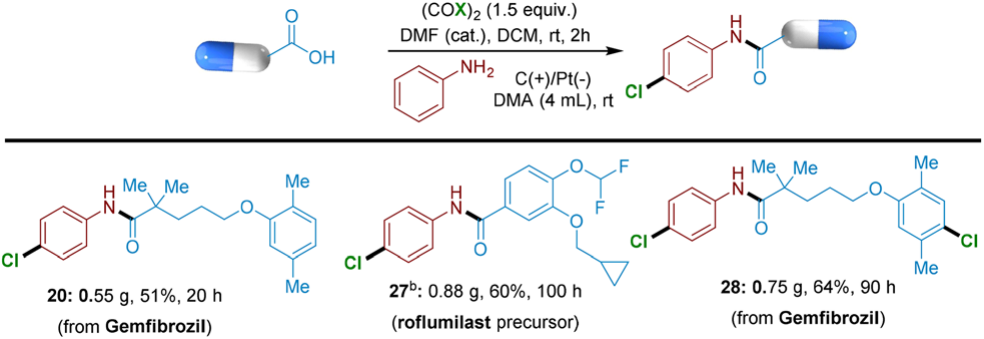
>500 mg scale examples involving bioactive molecules^*a*^. Reagents and conditions: ^*a*^first carboxylic acid (6.3 mmol), (COCl)_2_ (9.45 mmol) and DMF (cat.) in DCM (20 mL), rt, 2 h, followed by aniline (3.0 mmol) in DMA (30 mL), 20 mA in an undivided cell with carbon cloth (anode and cathode); ^*b*^carboxylic acid (4.2 mmol), (COCl)_2_ (6.3 mmol), DCM (20 mL), DMF (cat.), rt, 2 h, and aniline (4.0 mmol) in DMA (30 mL), 20 mA in an undivided cell with carbon cloth (anode and cathode); all yields refer to isolated yields.

Interestingly, our electrochemical one-pot protocol exhibited excellent scalability, as demonstrated by the preparation of the pharmaceutically relevant compounds 20, 27, and 28 on a >500 mg scale. Notably, in all the large-scale examples, a CC(+)/CC(−) electrode pairing served as a cost-effective alternative to CC(+)/Pt(−) without compromising reaction performance. Overall, the results summarized in Schemes 3 and 4 clearly underscore the broad synthetic utility of our acylation/C–H halogenation one-pot protocol, which enables rapid access to both established pharmaceutical agents and structurally novel analogues never previously investigated for their potential biological activity.

**Fig. 2:**
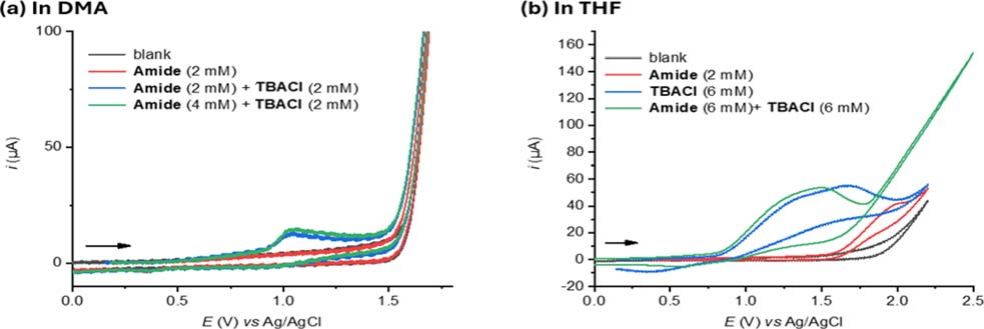
Cyclic voltammetry studies in DMA (a) and THF (b). Measurements were carried out using *n*-Bu_4_NBF_4_ (0.1 M) as the supporting electrolyte in anhydrous, degassed solvent at 20 °C, employing a 3 mm glassy carbon working electrode, a platinum wire counter electrode, and an Ag/AgCl (3 M NaCl) reference electrode, with a scan rate of 100 mV s^−1^. “Amide” refers to *N*-phenylcyclohexanecarboxamide. Adapted from Ponra *et al.*, *Angew. Chem. Int. Ed.*, 2026, DOI: https://doi.org/10.1002/anie.9028210.48. Licensed under CC BY 4.0.

To gain insight into the underlying pathway of our one-pot protocol, a series of control and monitoring experiments were conducted. The first step involves the well-established formation of an acyl halide from the corresponding carboxylic acid using oxalyl halide.^51^ NMR and LC-MS analyses of mixtures containing the amine and the *in situ* generated acyl halide in DMA prior to electrolysis confirmed rapid amide formation. Upon application of current, LC-MS monitoring revealed the gradual formation of the *N*-aryl halogenated products, accompanied by a concomitant decrease in the concentration of the *N*-aryl amide. These observations suggest that the *in situ* generated acyl halide undergoes a sequential process involving amide formation followed by electrochemically induced C–H halogenation, mediated by electrophilic halogen species generated at the anode. To decisively rule out the different reaction mechanism from an initial anodic oxidation of the transient amide's aromatic core, we performed cyclic voltammetry (CV) experiments (Fig. 2).^49^ Unfortunately, the CV analysis in DMA was complicated by the solvent oxidation limit (“solvent wall”, Fig. 2a), which prevented us from observing the oxidation peak potential of *N*-phenylcyclohexanecarboxamide. This called for the CV analysis to be performed in tetrahydrofuran (THF) instead, where the oxidation peak could be clearly observed approximately 1.8 V *vs.* Ag/AgCl (Fig. 2b). Upon addition of tetrabutylammonium chloride (TBACl) as a chloride source, an additional oxidation peak could be observed in both solvents at *ca* 1.0 V *vs.* Ag/AgCl, indicating the oxidation of Cl^−^ occurring before oxidation of the *in situ* formed amide. This observation is consistent with a C–H halogenation mechanism proceeding *via* anodic oxidation of chloride ions to electrophilic chlorine species, in agreement with multiple previous studies of related electrochemical halogenation reactions.^31–46^

Regarding the identity of the electrophilic species involved, we previously conducted a detailed DFT study on a related protocol to distinguish between mechanistic pathways involving either Cl^+^ or Cl_2_.^49^ These investigations indicated that Cl_2_, generated through anodic oxidation of chloride ions, serves as the active chlorinating species in an electrophilic aromatic substitution (EAS)-type C–H halogenation pathway. In agreement with this mechanism, the experimentally observed selectivity toward *para*-chlorinated products can be rationalized by DFT calculations showing that the σ-complex intermediate leading to *para* substitution is energetically favored over the corresponding *ortho* isomer by approximately 5 kcal mol^−1^, while the *meta* pathway is strongly disfavored. Additional computational analysis further suggested that DMA facilitates heterolytic cleavage of Cl_2_ and stabilizes charged intermediates through hydrogen bonding and noncovalent interactions.

**Scheme 5:**
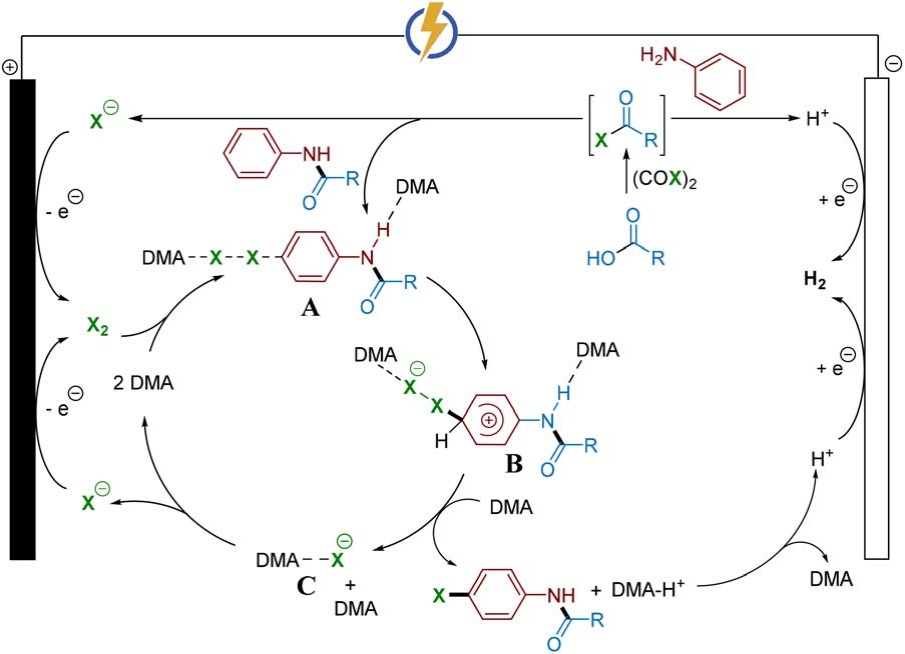
Plausible reaction pathway of electrochemical cascade *para*-halogenation.

Based on these mechanistic investigations,^49^ we propose a mechanism for this one-pot sequence as outlined in Scheme 5. Under the developed reaction conditions, the acid halide is first formed *via* the reaction of the carboxylic acid with oxalyl halide in the presence of a catalytic amount of DMF at room temperature. Upon addition of the amine, the corresponding amide is generated through reaction with the acyl halide, releasing a halide anion that can participate in the subsequent electrochemical C–H halogenation. Anodic oxidation of this halide anion generates molecular halogen (X_2_), and interacts with DMA and the aromatic ring of the amide in the solution phase (A). Next, DMA-assisted heterolytic cleavage of X_2_ affords a transient σ-complex intermediate, preferentially in the *para*-configuration (B), which undergoes deprotonation by DMA. Finally, the released, DMA-stabilized X^−^ (C) is re-oxidized at the anode to regenerate X_2_, which can enter the next reaction cycle. This mechanism highlights the multiple roles of DMA in the reaction, enabling highly efficient electrochemical halogenation of complex molecular scaffolds.

## Conclusions

Herein, we describe a mild, electrolyte-free electrochemical acylation/C–H halogenation one-pot protocol that enables the direct conversion of readily available carboxylic acids into halogenated *N*-aryl amides. By repurposing halide ions released during amidation as the halogen source for anodic C–H functionalization, the method integrates acyl activation and aryl halogenation without the need for external halogenating reagents, metal catalysts, or stoichiometric oxidants. The transformation proceeds under mild conditions in an undivided cell, exhibits broad substrate scope and high regioselectivity, and is applicable to both the late-stage functionalization of pharmaceutically relevant compounds and gram-scale synthesis. This work highlights how electrochemistry can be leveraged to merge carboxylic acid activation, amide bond formation and C–H halogenation into a streamlined and resource-efficient one-pot sequence. Further studies aimed at expanding the scope and applications of this cascade strategy are currently ongoing in our laboratory.

## Author contributions

SP and OV conceived the project. SP initiated a feasibility study of this project. SP optimized the reaction conditions, designed the experiments, surveyed the substrate scope, purification and isolation of products, analysed the data, and discussed the results with OV. SP and OV wrote the manuscript. OV gathered the research funding for this project and supervised it.

## Conflicts of interest

There are no conflicts to declare.

## Supplementary Material

RA-016-D6RA04649E-s001

## Data Availability

All experimental details and characterization data supporting this article are available in the supplementary information (SI). Supplementary information is available. See DOI: https://doi.org/10.1039/d6ra04649e.
