# Supplementary material for: Green electrochemistry-enabled one-pot synthesis of halogenated amides from readily available carboxylic acids
Source: RSC Adv. 2026 Jul 9;16(37):39505–11. doi: 10.1039/d6ra04649e (PMC13348519; doi:10.1039/d6ra04649e)
Supplement: Supplementary file 1 [file RA-016-D6RA04649E-s001.pdf]

Supplementary Information (SI) for RSC Advances.
This journal is © The Royal Society of Chemistry 2026

Green Electrochemistry-Enabled One-Pot Synthesis of Halogenated Amides from Readily Available Carboxylic Acids

Sudipta Ponra,*† Oscar Verho*†

† Uppsala Biomedical Centre, Department of Medicinal Chemistry, Uppsala University, SE-

E-mail: oscar.verho@ilk.uu.se, sudiptaponra@gmail.com

Table of contents

Experimental section....................S1

General chemistry information....................S1

General procedure A: One-pot cascade acylation/chlorination....................S2

General procedure B: One-pot cascade acylation/bromination....................S3

Cascades involving pharmaceutically-relevant molecules....................S8

General procedure C: Scale-up....................S15

List of reported compounds....................S17

References....................S17

NMR spectra… S18

General information. All substrates, reagents, and solvents were commercially available and used without further purification. Heating was carried out using a 17.4 mm DrySyn reaction vial insert compatible with 10 mL microwave vials. Purifications were performed on an automated Biotage Isolera Flash Chromatography System using 25 or 10 g prepacked Biotage SNAP KP-SIL columns. Accurate mass values were determined on a mass spectrometer equipped with an electrospray ion source and TOF detector. NMR spectra were recorded on a Bruker Avance III HD at 25 °C and 400 MHz for ^{1}H, 101 MHz for ^{13}C, and ^{19}F at 376.5 MHz using a SmartProbe BB/^{1}H probe or on a Varian Mercury plus at 25 °C and 400 MHz for ^{1}H, 101 MHz for ^{13}C, and ^{19}F at 376.5 MHz. Chemical shifts (δ) are reported in ppm, indirectly referenced to tetramethylsilane (TMS) via the residual solvent signal (^{1}H: $CHCl_3$ δ 7.26, CD_3OD δ 3.31, ^{13}C: $CDCl_3$ δ 77.2, CD_3OD δ 49.0). Electrocatalytic reactions were performed

with platinum (Pt) electrodes (25 mm × 10 mm × 0.125 mm, 99.9%; obtained from redox.me, Sweden) and carbon cloth (C) electrodes (15 mm × 10 mm × 410 µm; obtained from Fuel Cell Store, USA) connected by stainless steel adapters. Electrocatalytic reactions were conducted using an AX-3005PQ programmable power supply from AXIOMET.

General procedure: Generation of acid halide followed by cascade acylation/halogenation[a]

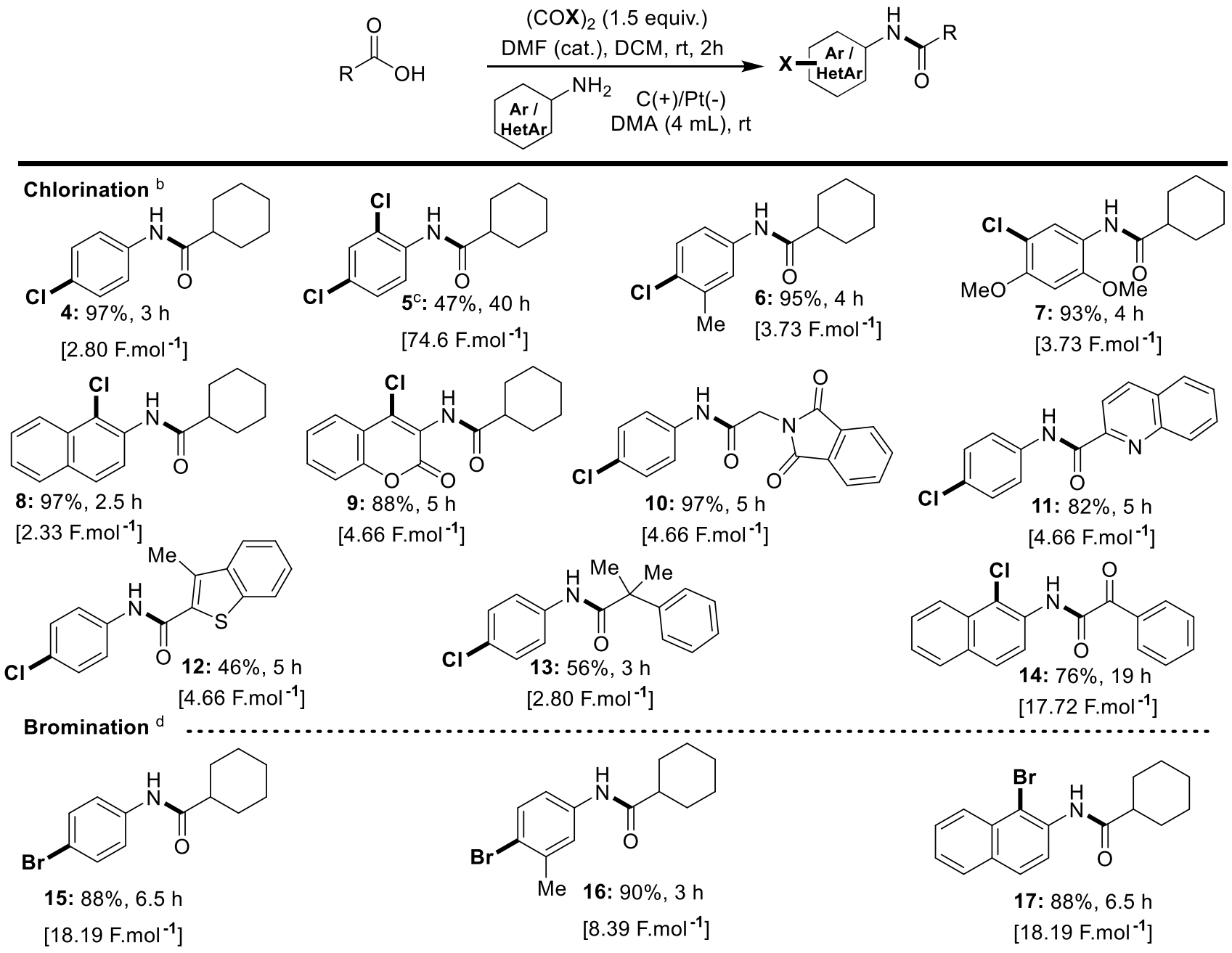

Reagents and conditions: [a]First carboxylic acid (0.21 mmol), $(COX)_2$ (0.315 mmol) and DMF (cat.) in DCM (2 mL), rt, 2 h, then removal of DCM under reduced pressure followed by amine (0.2 mmol) in DMA (4 mL), in an undivided cell with carbon cloth (anode) and platinum (cathode). [b]$(COCl)_2$, 5 mA. [c]$(COCl)_2$ (0.63 mmol). [d]$(COBr)_2$ (2M in DCM), 15 mA. All yields refer to isolated yields.

General procedure A: The electrolysis was carried out in 10 mL microwave reaction vial in an undivided cell setup under air. A carbon cloth anode (15 mm × 10 mm × 410 µm) and a platinum (Pt) cathode (25 mm × 10 mm × 0.125 mm) with electrode holders made of stainless steel were used and the distance between two electrodes was 10 mm. The cell was charged with the acid (0.21 mmol, 1.05 equiv.) and a teflon-coated magnetic stirring bar (15 × 6 mm), after which DCM (2 mL) and DMF (one drop) were added to the reaction vessel and cooled down to 0 ^{o}C. To this cooled and stirring solution, $(COCl)_2$ (26 µL, 0.315 mmol, 1.5 equiv.) was added dropwise and the reaction mixture was stirred at room temperature for 2 h. After 2 h, DCM was removed completely under reduced pressure and DMA (2 mL) was added. The resulting solution was placed under stirring and to it was slowly added amine (0.20 mmol, 1.0 equiv.) dissolved in DMA (2 mL). Subsequently, the electrolysis was performed at room temperature with a constant current of 5.0 mA that was maintained for 3 h (unless otherwise stated) with a stirring rate of 600 rpm. After completion of the reaction, the reaction mixture was diluted with ethyl acetate (2 mL) and transferred to a round bottom flask. The electrodes (carbon cloth and platinum) were washed in the reaction flask with ethyl acetate (3 × 5 mL) in an ultrasonic cleaner (3 ×

3 min) and the washes were combined in the round bottom flask. The reaction mixture was then transferred to a separating funnel and washed with brine (15 mL). The aqueous layer was washed with ethyl acetate (3 x 15 mL) and the combined organic layer was washed with brine (5 x 30 mL). The organic layer was dried with sodium sulfate and the solvent was removed under vacuum to obtain crude product. The crude products were purified by flash column chromatography using isohexane and ethyl acetate mixture (20:1 to 3:1) to afford the title compounds.

General procedure B: The electrolysis was carried out in 10 mL microwave reaction vial in an undivided cell setup under air. A carbon cloth anode (15 mm × 10 mm × 410 μm) and a platinum (Pt) cathode (25 mm × 10 mm × 0.125 mm) with electrode holders made of stainless steel were used and the distance between two electrodes was 10 mm. The cell was charged with the acid (0.21 mmol, 1.05 equiv.) and a teflon-coated magnetic stirring bar (15 × 6 mm), after which DCM (2 mL) and DMF (one drop) were added to the reaction vessel and cooled down to 0 °C. To this cooled and stirring solution $(COBr)_2$ (2.0 M in DCM, 158 μL, 0.315 mmol, 1.5 equiv.) was added dropwise and the reaction mixture was stirred at room temperature for 2 h. After 2 h, DCM was removed completely under reduced pressure and DMA (2 mL) was added. The resulting solution was placed under stirring and to it was slowly added amine (0.20 mmol, 1.0 equiv.) dissolved in DMA (2 mL). Subsequently, the electrolysis was performed at room temperature with a constant current of 5.0 mA that was maintained for 3 h (unless otherwise stated) with a stirring rate of 600 rpm. After completion of the reaction, the reaction mixture was diluted with ethyl acetate (2 mL) and transferred to a round bottom flask. The electrodes (carbon cloth and platinum) were washed in the reaction flask with ethyl acetate (3 × 5 mL) in an ultrasonic cleaner (3 × 3 min) and the washes were combined in the round bottom flask. The reaction mixture was then transferred to a separating funnel and washed with brine (15 mL). The aqueous layer was washed with ethyl acetate (3 x 15 mL) and the combined organic layer was washed with brine (5 x 30 mL). The organic layer was dried with sodium sulfate and the solvent was removed under vacuum to obtain crude product. The crude products were purified by flash column chromatography using isohexane and ethyl acetate mixture (20:1 to 3:1) to afford the title compounds.

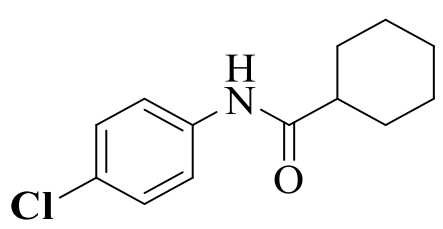

***N*-(4-chlorophenyl)cyclohexanecarboxamide (4)**[1a] was synthesized using 26.0 mg of cyclohexane carboxylic acid, 26.0 μL of $(COCl)_2$, 18.6 mg of aniline and following general procedure A using 5 mA constant current for 3 h. Compound **4** was isolated as a white solid (46 mg, 97% yield) following work-up without the need for any further purification. ^{1}H NMR (400 MHz, DMSO-d_6) δ 9.92 (s, 1H), 7.70 – 7.50 (m, 2H), 7.37 – 7.23 (m, 2H), 2.30 (tt, *J* = 11.8, 3.3 Hz, 1H), 1.77 (tt, *J* = 15.0, 2.2 Hz, 4H), 1.67 – 1.61 (m, 1H), 1.39 (qd, *J* = 13.5, 13.1, 3.5 Hz, 2H), 1.32 – 1.14 (m, 3H). ^{13}C NMR (101 MHz, DMSO-

d6) δ 174.4, 138.4, 128.5, 126.3, 120.5, 44.8, 29.1, 25.4, 25.2. HRMS (ESI): m/z $[M+H]^+$ calcd for $C_{13}H_{17}NOCl$: 238.0999; found: 238.0988.

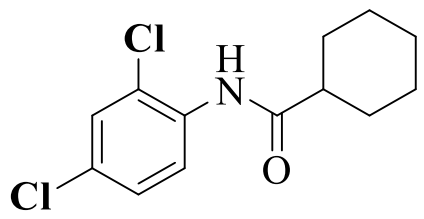

***N*-(2,4-dichlorophenyl)cyclohexanecarboxamide (5)**[1a] was synthesized using 26.0 mg of cyclohexane carboxylic acid, 52.0 μL of $(COCl)_2$, 18.6 mg of aniline and following general procedure A using 5 mA constant current for 40 h. Compound **5** was isolated using automated flash chromatography (silica gel, gradient elution 5-30% EtOAc in isohexane) as a white solid (25.5 mg, 47% yield). ^{1}H NMR (400 MHz, $CDCl_3$) δ 8.38 (d, *J* = 8.9 Hz, 1H), 7.64 (s, 1H), 7.37 (d, *J* = 2.4 Hz, 1H), 7.24 (dd, *J* = 8.9, 2.4 Hz, 1H), 2.30 (tt, *J* = 11.7, 3.5 Hz, 1H), 2.03 – 1.97 (m, 2H), 1.85 (dt, *J* = 13.1, 3.6 Hz, 2H), 1.72 (dddd, *J* = 11.2, 4.9, 2.9, 1.5 Hz, 1H), 1.52 (td, *J* = 12.3, 3.2 Hz, 2H), 1.37 – 1.25 (m, 3H). ^{13}C NMR (101 MHz, CDCl3) δ 174.4, 133.6, 128.9, 128.7, 128.0, 123.2, 122.3, 46.7, 29.8, 25.8, 25.7.

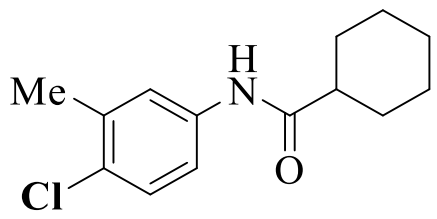

***N*-(4-chloro-3-methylphenyl)cyclohexanecarboxamide (6)**[1a] was synthesized using 26.0 mg of cyclohexane carboxylic acid, 26.0 μL of $(COCl)_2$, 21.4 mg of *m*-toluidine and following general procedure A using 5 mA constant current for 4 h. Compound **6** was isolated as a white solid (47.5 mg, 95% yield) following work-up without the need for any further purification. ^{1}H NMR (400 MHz, DMSO-d_6) δ 9.83 (s, 1H), 7.60 (d, *J* = 2.5 Hz, 1H), 7.44 (dd, *J* = 8.7, 2.6 Hz, 1H), 7.28 (d, *J* = 8.7 Hz, 1H), 2.31 (dt, *J* = 11.6, 3.2 Hz, 1H), 2.27 (s, 3H), 1.82 – 1.70 (m, 4H), 1.66 – 1.60 (m, 1H), 1.39 (qd, *J* = 13.7, 13.1, 3.5 Hz, 2H), 1.31 – 1.17 (m, 3H). ^{13}C NMR (101 MHz, DMSO-d_6) δ 174.3, 138.4, 135.4, 128.8, 126.6, 121.4, 118.2, 44.8, 29.1, 25.4, 25.2, 19.8. HRMS (ESI): m/z $[M+H]^+$ calcd for $C_{14}H_{19}NOCl$: 252.1155; found: 252.1147.

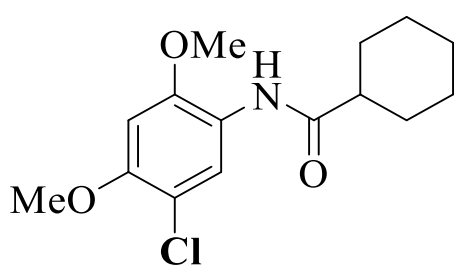

***N*-(5-chloro-2,4-dimethoxyphenyl)cyclohexanecarboxamide (7)**[1a] 26.0 mg of cyclohexane carboxylic acid, 26.0 μL of $(COCl)_2$, 30.6 mg of 2,4-dimethoxyaniline and following general procedure A using 5 mA constant current for 4 h. Compound **7** was isolated using automated flash chromatography (silica gel, gradient elution 5-30% EtOAc in isohexane) as an off-white solid (55.0 mg, 93% yield). ^{1}H NMR (400 MHz, $CDCl_3$) δ 8.45 (s, 1H), 7.57 (s, 1H), 6.49 (s, 1H), 3.89 (s, 3H), 3.86 (s, 3H), 2.23 (tt, *J* = 11.7, 3.5 Hz, 1H), 1.98 – 1.90 (m, 2H), 1.86 – 1.79 (m, 2H), 1.72 – 1.66 (m, 1H), 1.51 (qd, *J* = 12.1, 3.4 Hz, 2H), 1.37 – 1.24 (m, 3H). ^{13}C NMR (101 MHz, $CDCl_3$) δ 174.0, 151.1, 147.6, 121.8, 121.5,

113.8, 96.7, 56.8, 56.2, 46.7, 29.8, 25.8, 25.8. HRMS (ESI): m/z $[M+H]^+$ calcd for $C_{15}H_{21}NO_3Cl$: 298.1210; found: 298.1197.

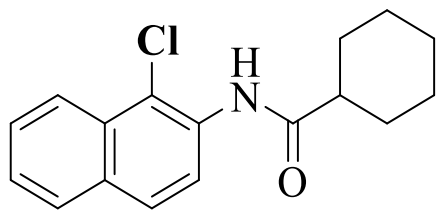

***N*-(1-chloronaphthalen-2-yl)cyclohexanecarboxamide (8)**[1a] 26.0 mg of cyclohexane carboxylic acid, 26.0 μL of $(COCl)_2$, 28.6 mg of naphthalen-2-amine and following general procedure A using 5 mA constant current for 2.5 h. Compound **8** was isolated as a pale brownish solid (55.5 mg, 97% yield) following work-up without the need for any further purification. ^{1}H NMR (400 MHz, $CDCl_3$) δ 8.56 (dd, *J* = 9.0, 4.0 Hz, 1H), 8.17 (dt, *J* = 8.6, 0.9 Hz, 1H), 7.99 – 7.90 (m, 1H), 7.87 – 7.72 (m, 2H), 7.58 (ddd, *J* = 8.4, 6.8, 1.3 Hz, 1H), 7.46 (ddd, *J* = 8.1, 6.8, 1.2 Hz, 1H), 2.39 (tt, *J* = 11.7, 3.5 Hz, 1H), 2.13 – 2.01 (m, 2H), 1.88 (dt, *J* = 12.4, 3.3 Hz, 2H), 1.74 (dtd, *J* = 10.8, 3.1, 1.5 Hz, 1H), 1.66 – 1.55 (m, 2H), 1.35 (dddd, *J* = 23.6, 15.3, 12.3, 9.0 Hz, 3H). ^{13}C NMR (101 MHz, $CDCl_3$) δ 174.6, 133.0, 131.2, 130.7, 128.2, 127.6, 127.5, 125.5, 123.9, 120.7, 118.2, 46.8, 29.8, 25.8, 25.8. HRMS (ESI): m/z $[M+H]^+$ calcd for $C_{17}H_{19}NOCl$: 288.1155; found: 288.1145.

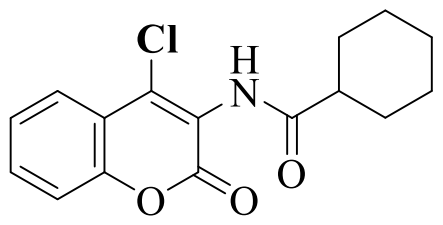

***N*-(4-chloro-2-oxo-2H-chromen-3-yl)cyclohexanecarboxamide (9)**[1a] 26.0 mg of cyclohexane carboxylic acid, 26.0 μL of $(COCl)_2$, 32.2 mg of 3-amino-2*H*-chromen-2-one and following general procedure A using 5 mA constant current for 5 h. Compound **9** was isolated using automated flash chromatography (silica gel, gradient elution 5-30% EtOAc in isohexane) as a white solid (53.5 mg, 88% yield). ^{1}H NMR (400 MHz, $CDCl_3$) δ 7.91 (dd, *J* = 8.0, 1.5 Hz, 1H), 7.57 (ddd, *J* = 8.6, 7.4, 1.6 Hz, 1H), 7.42 – 7.34 (m, 2H), 7.20 (s, 1H), 2.39 (tt, *J* = 11.6, 3.6 Hz, 1H), 2.06 – 1.99 (m, 2H), 1.85 (dt, *J* = 11.7, 3.3 Hz, 2H), 1.73 – 1.68 (m, 1H), 1.64 – 1.52 (m, 2H), 1.40 – 1.24 (m, 3H). ^{13}C NMR (101 MHz, $CDCl_3$) δ 173.8, 158.7, 150.5, 141.1, 132.2, 125.9, 125.3, 121.5, 118.9, 116.8, 45.8, 29.6, 25.8, 25.7. LC-MS: m/z $[M+H]^+$ calcd for $C_{16}H_{17}NO_3Cl$: 306.1; found: 306.1.

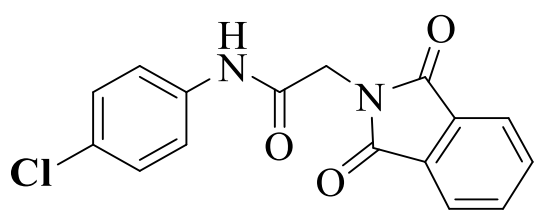

***N*-(4-chlorophenyl)-2-(1,3-dioxoisoindolin-2-yl)acetamide (10)** was synthesized using 43.0 mg of 2-(1,3-dioxoisoindolin-2-yl)acetic acid, 26.0 μL of $(COCl)_2$, 18.6 mg of aniline and following general procedure A using 5 mA constant current for 5 h. Compound **10** was isolated using automated flash chromatography (silica gel, gradient elution 5-30% EtOAc in isohexane) as a white solid (61 mg, 97% yield). ^{1}H NMR (400 MHz, DMSO-d_6) δ 10.47 (s, 1H), 8.00 – 7.83 (m, 4H), 7.62 – 7.52 (m, 2H), 7.42 – 7.31 (m, 2H), 4.45 (s, 2H). ^{13}C NMR (101 MHz, DMSO-d_6) δ 167.5, 165.0, 137.4, 134.7, 131.6, 128.8, 127.2, 123.3, 120.8, 40.7.

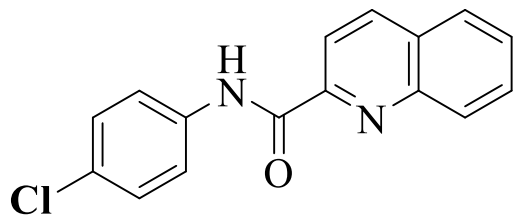

***N*-(4-chlorophenyl)quinoline-2-carboxamide (11)** was synthesized using 36.3 mg of quinoline-2-carboxylic acid, 26.0 μL of $(COCl)_2$, 18.6 mg of aniline and following general procedure A using 5 mA constant current for 5 h. Compound **11** was isolated using automated flash chromatography (silica gel, gradient elution 5-30% EtOAc in isohexane) as a white solid (46 mg, 82% yield). ^{1}H NMR (400 MHz, $CDCl_3$) δ 10.2 (s, 1H), 8.39 – 8.32 (m, 2H), 8.18 – 8.14 (m, 1H), 7.91 – 7.88 (m, 1H), 7.83 – 7.77 (m, 3H), 7.64 (ddd, J = 8.2, 6.9, 1.2 Hz, 1H), 7.39 – 7.34 (m, 2H). ^{13}C NMR (101 MHz, $CDCl_3$) δ 162.2, 149.4, 146.3, 138.0, 136.5, 130.5, 129.7, 129.6, 129.2, 128.4, 127.9, 121.0, 119.8, 118.8.

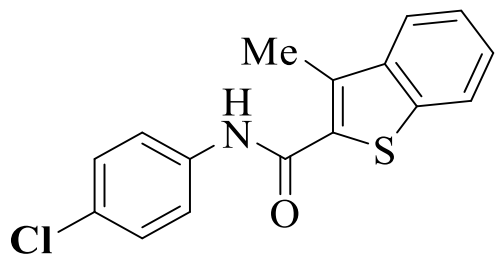

***N*-(4-chlorophenyl)-3-methylbenzo[*b*]thiophene-2-carboxamide (12)** was synthesized using 40.3 mg of 3-methylbenzo[*b*]thiophene-2-carboxylic acid, 26.0 μL of $(COCl)_2$, 18.6 mg of aniline and following general procedure A using 5 mA constant current for 5 h. Compound **12** was isolated using automated flash chromatography (silica gel, gradient elution 5-30% EtOAc in isohexane) as a white solid (27.7 mg, 46% yield). ^{1}H NMR (400 MHz, $CDCl_3$) δ 7.80 – 7.75 (m, 2H), 7.56 (d, J = 5.0 Hz, 1H), 7.53 – 7.48 (m, 2H), 7.43 – 7.39 (m, 2H), 7.30 – 7.26 (m, 2H), 2.70 (s, 3H). ^{13}C NMR (101 MHz, $CDCl_3$) δ 161.7, 140.7, 138.5, 138.1, 136.3, 129.9, 129.3, 127.2, 125.1, 123.7, 122.8, 121.6, 120.4, 13.3. HRMS (ESI): m/z $[M+H]^+$ calcd for $C_{16}H_{13}NOSCl$: 302.0406; found: 302.0399.

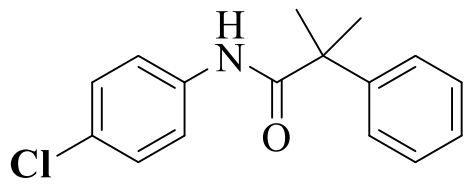

***N*-(4-chlorophenyl)-2-methyl-2-phenylpropanamide (13)** was synthesized using 34.4 mg of 2-methyl-2-phenylpropanoic acid, 26.0 μL of $(COCl)_2$, 18.6 mg of aniline and following general procedure A using 5 mA constant current for 3 h. Compound **13** was isolated using automated flash chromatography (silica gel, gradient elution 5-30% EtOAc in isohexane) as a white solid (30.5 mg, 56% yield). ^{1}H NMR (400 MHz, $CDCl_3$) δ 7.46 – 7.38 (m, 4H), 7.35 – 7.28 (m, 3H), 7.23 – 7.19 (m, 2H), 6.77 (s, 1H), 1.66 (s, 6H). ^{13}C NMR (101 MHz, $CDCl_3$) δ 175.8, 144.5, 136.7, 129.2, 129.0, 127.7, 126.6, 121.0 (2C), 48.2, 27.1 (2C).

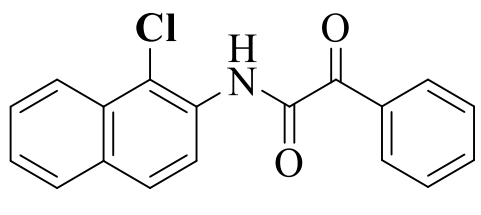

***N*-(1-chloronaphthalen-2-yl)-2-oxo-2-phenylacetamide (14)** was synthesized using 31.5 mg of 2-oxo-2-phenylacetic acid, 26.0 μL of $(COCl)_2$, 28.6 mg of naphthalen-2-amine and following general procedure A using 5 mA constant current for 19 h. Compound **14** was isolated using automated flash

chromatography (silica gel, gradient elution 5-30% EtOAc in isohexane) as a yellow solid (47 mg, 76% yield). ^{1}H NMR (400 MHz, $CDCl_3$) δ 9.83 (s, 1H), 8.67 (d, *J* = 9.0 Hz, 1H), 8.52 – 8.41 (m, 2H), 8.24 (dd, *J* = 8.6, 1.1 Hz, 1H), 7.88 – 7.84 (m, 2H), 7.71 – 7.66 (m, 1H), 7.62 (ddd, *J* = 8.4, 6.8, 1.3 Hz, 1H), 7.53 (dddd, *J* = 9.7, 8.1, 6.9, 1.4 Hz, 3H). ^{13}C NMR (101 MHz, $CDCl_3$) δ 187.0, 159.1, 134.9, 133.1, 131.8, 131.8, 131.7, 131.7, 130.8, 128.8, 128.3, 127.9, 126.2, 124.2, 119.9, 119.6. HRMS (ESI): m/z $[M-H]^-$ calcd for $C_{18}H_{13}NO_2Cl$: 308.0484; found: 308.0485.

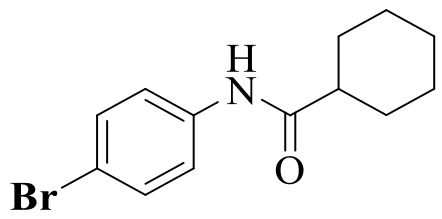

***N*-(4-bromophenyl)cyclohexanecarboxamide (15)**[1a] was synthesized using 26.0 mg of cyclohexane carboxylic acid, 158.0 μL of $(COBr)_2$ (2.0 M in DCM), 18.6 mg of aniline and following general procedure B using 15 mA constant current for 6.5 h. Compound **15** was isolated using automated flash chromatography (silica gel, gradient elution 20-40% EtOAc in isohexane) as a white solid (49.4 mg, 88% yield). ^{1}H NMR (400 MHz, $CDCl_3$) δ 7.45 – 7.38 (m, 4H), 7.29 (s, 1H), 2.22 (tt, *J* = 11.7, 3.5 Hz, 1H), 1.97 – 1.90 (m, 2H), 1.83 (dq, *J* = 13.7, 3.7 Hz, 2H), 1.73 – 1.67 (m, 1H), 1.53 (qd, *J* = 12.0, 3.3 Hz, 2H), 1.35 – 1.23 (m, 3H). ^{13}C NMR (101 MHz, $CDCl_3$) δ 174.5, 137.3, 132.0, 121.4, 116.7, 46.7, 29.8, 25.8 (2C). HRMS (ESI): m/z $[M+H]^+$ calcd for $C_{13}H_{17}NOBr$: 282.0494; found: 282.0497.

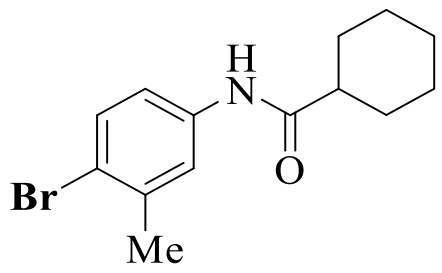

***N*-(4-bromo-3-methylphenyl)cyclohexanecarboxamide (16)**[1a] was synthesized using 26.0 mg of cyclohexane carboxylic acid, 158.0 μL of $(COBr)_2$ (2.0 M in DCM), 21.4 mg of *m*-toluidine and following general procedure B using 15 mA constant current for 3.0 h. Compound **16** was isolated using automated flash chromatography (silica gel, gradient elution 20-40% EtOAc in isohexane) as a white solid (53.0 mg, 90% yield). ^{1}H NMR (400 MHz, $CDCl_3$) δ 7.49 (d, *J* = 2.6 Hz, 1H), 7.42 (d, *J* = 8.6 Hz, 1H), 7.21 – 7.15 (m, 2H), 2.35 (s, 3H), 2.21 (tt, *J* = 11.7, 3.5 Hz, 1H), 1.97 – 1.91 (m, 2H), 1.83 (dq, *J* = 13.6, 3.7 Hz, 2H), 1.73 – 1.66 (m, 1H), 1.53 (qd, *J* = 12.1, 3.3 Hz, 2H), 1.36 – 1.21 (m, 3H). ^{13}C NMR (101 MHz, $CDCl_3$) δ 174.5, 138.6, 137.4, 132.7, 122.1, 119.3, 118.8, 46.7, 29.8, 25.8, 25.8, 23.2. HRMS (ESI): m/z $[M+H]^+$ calcd for $C_{14}H_{19}NOBr$: 296.0650; found: 296.0652.

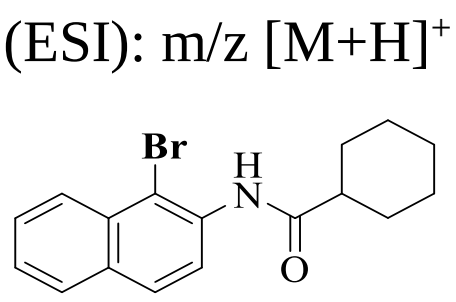

***N*-(1-bromonaphthalen-2-yl)cyclohexanecarboxamide (17)**[1a] was synthesized using 26.0 mg of cyclohexane carboxylic acid, 158.0 μL of $(COBr)_2$ (2.0 M in DCM), 28.6 mg of naphthalen-2-amine and following general procedure B using 15 mA constant current for 6.5 h. Compound **17** was isolated using automated flash chromatography (silica gel, gradient elution 20-40% EtOAc in isohexane) as a

white solid (58.2 mg, 88% yield). ^{1}H NMR (400 MHz, $CDCl_3$) δ 8.51 (d, J = 9.0 Hz, 1H), 8.15 (dd, J = 8.6, 1.1 Hz, 1H), 7.99 (s, 1H), 7.80 (dd, J = 8.9, 2.2 Hz, 2H), 7.57 (ddd, J = 8.4, 6.9, 1.3 Hz, 1H), 7.50 – 7.44 (m, 1H), 2.39 (tt, J = 11.7, 3.5 Hz, 1H), 2.12 – 2.05 (m, 2H), 1.89 (dq, J = 12.6, 3.4 Hz, 2H), 1.74 (dtd, J = 10.8, 3.3, 1.7 Hz, 1H), 1.60 (qd, J = 12.1, 3.3 Hz, 2H), 1.35 (dddd, J = 24.0, 15.2, 12.2, 9.0 Hz, 3H). ^{13}C NMR (101 MHz, $CDCl_3$) δ 174.6, 134.6, 132.1, 131.6, 128.4, 128.3, 127.8, 126.6, 125.5, 121.1, 111.7, 46.9, 29.9, 25.8, 25.8. HRMS (ESI): m/z $[M+H]^+$ calcd for $C_{17}H_{19}NOBr$: 332.0650; found: 332.0657.

Cascades involving pharmaceutically-relevant molecules [a]

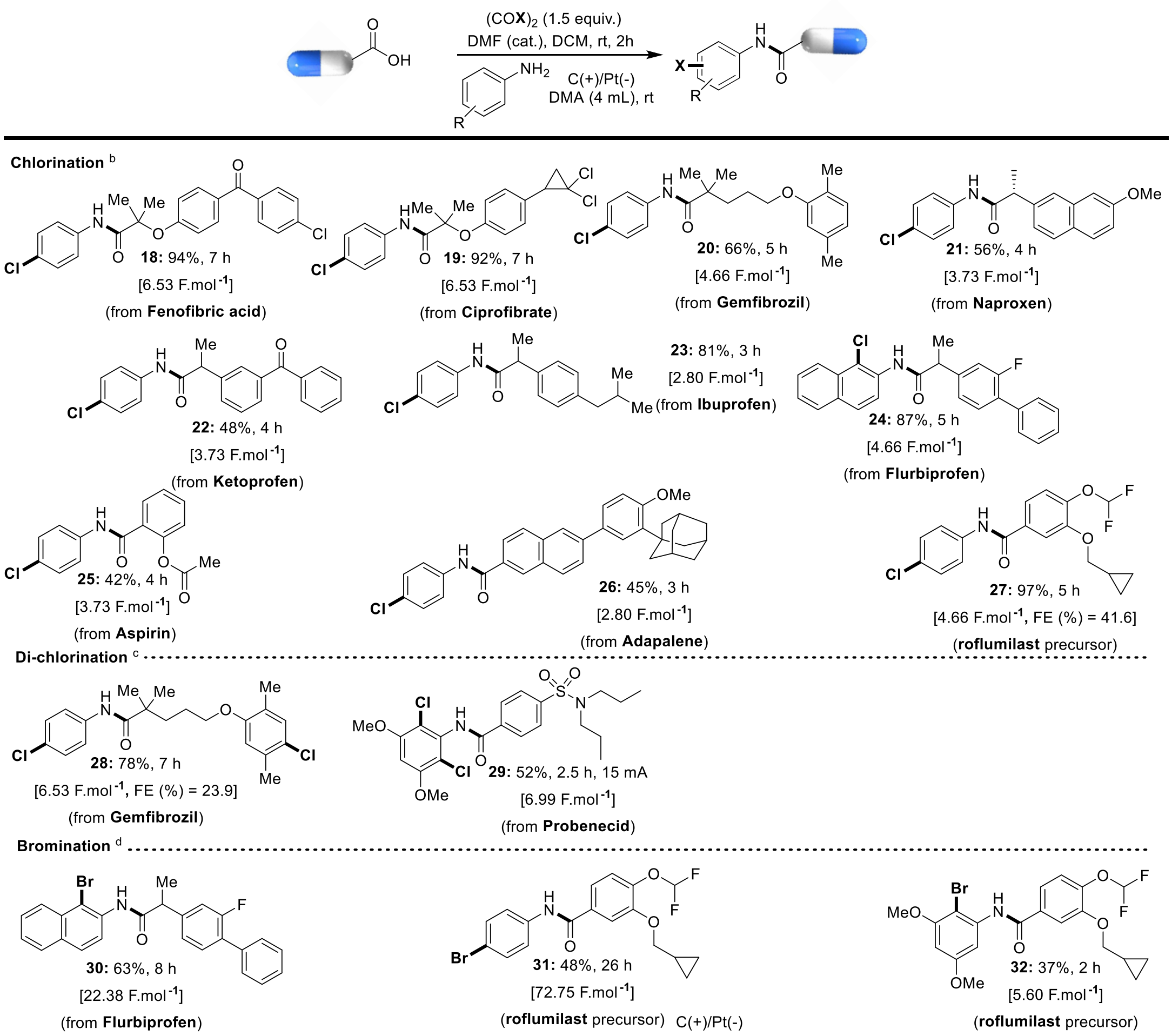

Reagents and conditions: [a] First carboxylic acid reagent (0.21 mmol), $(COX)_2$ (0.315 mmol), DMF (cat.), DCM (2 mL), rt, 2 h, then removal of DCM under reduced pressure followed by amine (0.2 mmol) in DMA (4 mL), in an undivided cell with carbon cloth (anode) and platinum (cathode). [b] $(COCl)_2$, 5 mA; [c] Carboxylic acid (0.42 mmol), $(COCl)_2$ (0.63 mmol) DCM (2 mL), DMF (cat.), rt, 2h, and amine (0.2 mmol) in DMA (4 mL), 5 mA; [d] $(COBr)_2$ (2M in DCM), 15 mA. All yields refer to isolated yields.

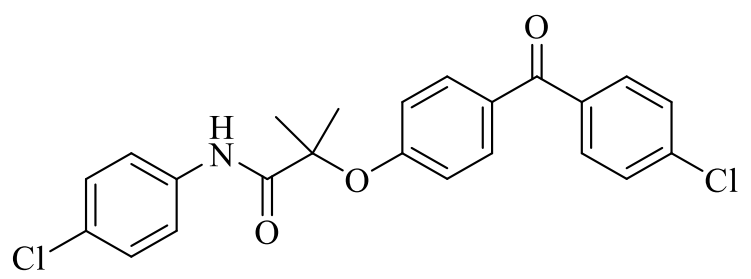

2-(4-(4-chlorobenzoyl)phenoxy)-*N*-(4-chlorophenyl)-2-methylpropanamide (18) was synthesized using 66.8 mg of 2-(4-(4-chlorobenzoyl)phenoxy)-2-methylpropanoic acid, 26.0 μL of $(COCl)_2$, 18.6 mg of aniline and following general procedure A using 5 mA constant current for 7 h. Compound **18** was isolated using automated flash chromatography (silica gel, gradient elution 5-30% EtOAc in isohexane) as a white solid (80 mg, 94% yield). ^{1}H NMR (400 MHz, $CDCl_3$) δ 8.30 (s, 1H), 7.71 – 7.61 (m, 4H), 7.45 – 7.35 (m, 4H), 7.24 – 7.18 (m, 2H), 6.98 – 6.93 (m, 2H), 1.59 (s, 6H). ^{13}C NMR (101 MHz, $CDCl_3$) δ 194.2, 172.4, 158.1, 138.9, 136.0, 135.9, 132.4, 132.1, 131.3, 129.8, 129.2, 128.8, 121.3, 120.3, 82.5, 25.2. HRMS (ESI): m/z $[M+H]^+$ calcd for $C_{23}H_{20}NO_3Cl_2$: 428.0820; found: 428.0808.

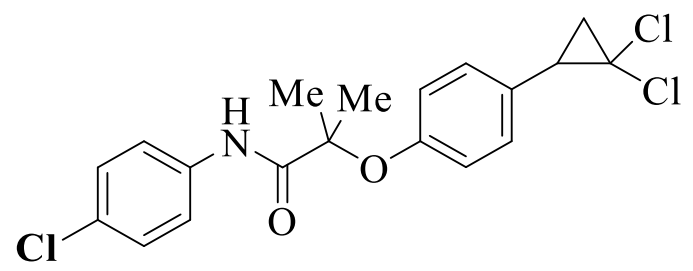

***N*-(4-chlorophenyl)-2-(4-(2,2-dichlorocyclopropyl)phenoxy)-2-methylpropanamide (19)** was synthesized using 60.5 mg of 2-(4-(2,2-dichlorocyclopropyl)phenoxy)-2-methylpropanoic acid, 26.0 μL of $(COCl)_2$, 18.6 mg of aniline and following general procedure A using 5 mA constant current for 7 h. Compound **19** was isolated using automated flash chromatography (silica gel, gradient elution 5-30% EtOAc in isohexane) as a white solid (73 mg, 92% yield). ^{1}H NMR (400 MHz, $CDCl_3$) δ 8.58 (s, 1H), 7.53 (d, *J* = 8.8 Hz, 2H), 7.29 (d, *J* = 8.8 Hz, 2H), 7.22 – 7.14 (m, 2H), 6.96 (d, *J* = 8.6 Hz, 2H), 2.86 (dd, *J* = 10.7, 8.3 Hz, 1H), 1.97 (dd, *J* = 10.7, 7.5 Hz, 1H), 1.81 (dd, *J* = 8.3, 7.4 Hz, 1H), 1.57 (s, 6H). ^{13}C NMR (101 MHz, $CDCl_3$) δ 173.0, 153.3, 136.1, 130.4, 130.0, 129.5, 129.1, 121.8, 121.2, 82.2, 60.8, 34.9, 26.0, 25.1, 25.0. HRMS (ESI): m/z $[M+H]^+$ calcd for $C_{19}H_{19}NO_2Cl_3$: 398.0481; found: 398.0488.

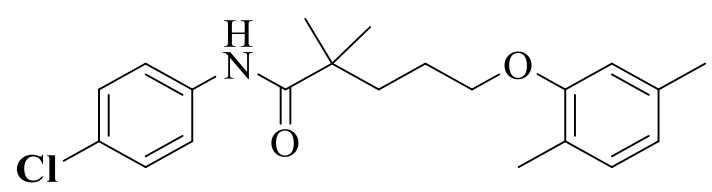

***N*-(4-chlorophenyl)-5-(2,5-dimethylphenoxy)-2,2-dimethylpentanamide (20)** was synthesized using 52.5 mg of 5-(2,5-dimethylphenoxy)-2,2-dimethylpentanoic acid, 26.0 μL of $(COCl)_2$, 18.6 mg of aniline and following general procedure A using 5 mA constant current for 5 h. Compound **20** was isolated using automated flash chromatography (silica gel, gradient elution 5-30% EtOAc in isohexane) as a white solid (47.4 mg, 66% yield). ^{1}H NMR (400 MHz, $CDCl_3$) δ 7.51 – 7.45 (m, 2H), 7.40 (s, 1H), 7.32 – 7.28 (m, 2H), 7.03 (d, *J* = 7.5 Hz, 1H), 6.69 (dd, *J* = 7.6, 1.7 Hz, 1H), 6.63 (d, *J* = 1.7 Hz, 1H), 3.97 (p, *J* = 2.5 Hz, 2H), 2.32 (s, 3H), 2.20 (s, 3H), 1.85 (q, *J* = 3.0, 2.6 Hz, 4H), 1.36 (s, 6H). ^{13}C NMR

(101 MHz, $CDCl_3$) δ 175.9, 156.9, 136.7, 136.6, 130.5, 129.4, 129.0, 123.6, 121.5, 121.0, 112.3, 67.9, 43.0, 37.7, 25.7, 25.2, 21.5, 16.0.

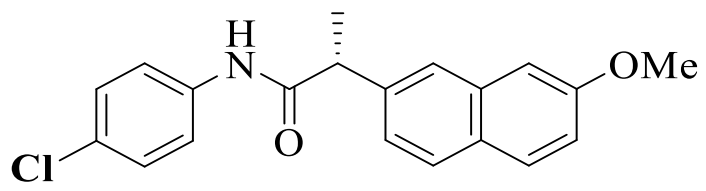

(*R*)-*N*-(4-chlorophenyl)-2-(7-methoxynaphthalen-2-yl)propanamide (21) was synthesized using 48.3 mg of (*R*)-2-(7-methoxynaphthalen-2-yl)propanoic acid, 26.0 μL of $(COCl)_2$, 18.6 mg of aniline and following general procedure A using 5 mA constant current for 4 h. Compound **21** was isolated using automated flash chromatography (silica gel, gradient elution 5-30% EtOAc in isohexane) as a white solid (38 mg, 56% yield). ^{1}H NMR (400 MHz, $CDCl_3$) δ 8.23 (d, *J* = 8.8 Hz, 1H), 7.76 (d, *J* = 8.9 Hz, 2H), 7.56 (dd, *J* = 8.7, 1.8 Hz, 1H), 7.43 – 7.31 (m, 3H), 7.28 – 7.19 (m, 2H), 7.12 – 7.03 (m, 2H), 4.04 (s, 3H), 3.87 (q, *J* = 7.1 Hz, 1H), 1.68 (d, *J* = 7.1 Hz, 3H). ^{13}C NMR (101 MHz, $CDCl_3$) δ 172.2, 152.9, 137.9, 136.9, 131.4, 129.7, 129.1, 128.0, 127.5, 126.6, 124.8, 124.5, 121.1, 119.8, 114.4, 57.1, 48.1, 18.7. HRMS (ESI): m/z $[M+H]^+$ calcd for $C_{20}H_{19}NO_2Cl$: 340.1104; found: 340.1112.

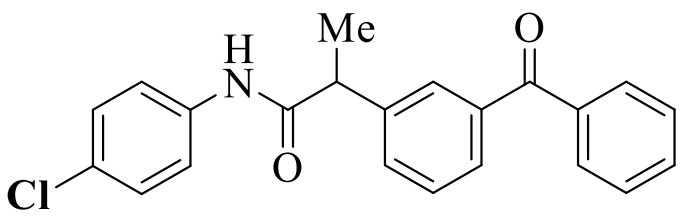

2-(3-benzoylphenyl)-*N*-(4-chlorophenyl)propanamide (22) was synthesized using 53.3 mg of 2-(3-benzoylphenyl)propanoic acid, 26.0 μL of $(COCl)_2$, 18.6 mg of aniline and following general procedure A using 5 mA constant current for 4 h. Compound **22** was isolated using automated flash chromatography (silica gel, gradient elution 5-30% EtOAc in isohexane) as a colorless sticky gum (35 mg, 48% yield). ^{1}H NMR (400 MHz, $CDCl_3$) δ 7.83 – 7.75 (m, 3H), 7.70 – 7.57 (m, 3H), 7.47 (td, *J* = 7.7, 2.0 Hz, 3H), 7.39 (dd, *J* = 8.3, 6.0 Hz, 3H), 7.25 – 7.20 (m, 2H), 3.76 (q, *J* = 7.1 Hz, 1H), 1.60 (d, *J* = 7.1 Hz, 3H). ^{13}C NMR (101 MHz, $CDCl_3$) δ 196.8, 171.8, 141.5, 138.3, 137.4, 136.5, 132.9, 131.6, 130.2, 129.7, 129.5, 129.3, 129.2, 129.1, 128.5, 121.2, 48.1, 18.9. HRMS (ESI): m/z $[M+H]^+$ calcd for $C_{22}H_{19}NO_2Cl$: 364.1104; found: 364.1100.

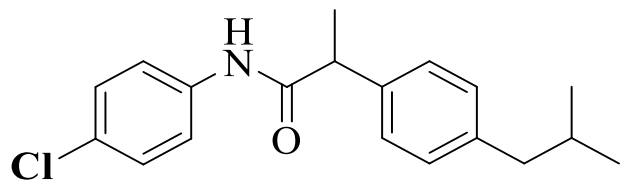

***N*-(4-chlorophenyl)-2-(4-isobutylphenyl)propanamide (23)** was synthesized using 43.3 mg of 2-(4-isobutylphenyl)propanoic acid, 26.0 μL of $(COCl)_2$, 18.6 mg of aniline and following general procedure A using 5 mA constant current for 3 h. Compound **23** was isolated using automated flash chromatography (silica gel, gradient elution 5-30% EtOAc in isohexane) as a white solid (51 mg, 81% yield). ^{1}H NMR (400 MHz, $CDCl_3$) δ 7.41 – 7.35 (m, 3H), 7.29 – 7.20 (m, 4H), 7.20 – 7.15 (m, 2H), 3.71 (q, *J* = 7.1 Hz, 1H), 2.50 (d, *J* = 7.2 Hz, 2H), 1.89 (dp, *J* = 13.5, 6.7 Hz, 1H), 1.60 (d, *J* = 7.2 Hz, 3H), 0.94 (d, *J* = 6.7 Hz, 6H). ^{13}C NMR (101 MHz, $CDCl_3$) δ 172.9, 141.3, 137.9, 136.6, 130.0, 129.2, 128.9, 127.5, 121.1, 47.7, 45.1, 30.3, 22.5, 18.6.

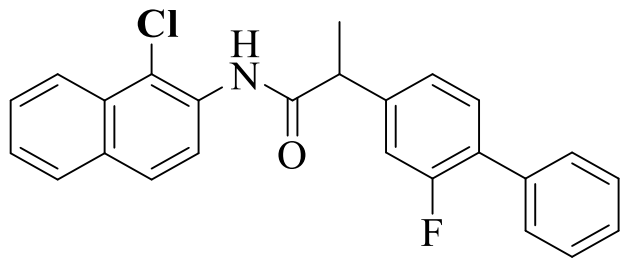

***N*-(1-chloronaphthalen-2-yl)-2-(2-fluoro-[1,1'-biphenyl]-4-yl)propanamide (24)** was synthesized using 51.2 mg of 2-(2-fluoro-[1,1'-biphenyl]-4-yl)propanoic acid, 26.0 μL of $(COCl)_2$, 28.6 mg of naphthalen-2-amine and following general procedure A using 5 mA constant current for 5 h. Compound **24** was isolated using automated flash chromatography (silica gel, gradient elution 5-30% EtOAc in isohexane) as a white solid (70 mg, 87% yield). ^{1}H NMR (400 MHz, $CDCl_3$) δ 8.52 (d, *J* = 9.0 Hz, 1H), 8.11 (dd, *J* = 8.5, 1.1 Hz, 1H), 7.93 (s, 1H), 7.83 – 7.73 (m, 2H), 7.60 – 7.43 (m, 7H), 7.42 – 7.36 (m, 1H), 7.34 – 7.24 (m, 2H), 3.89 (q, *J* = 7.2 Hz, 1H), 1.72 (d, *J* = 7.1 Hz, 3H). ^{13}C NMR (101 MHz, $CDCl_3$) δ 172.0, 161.4, 158.9, 141.9 (d, *J* = 7.5 Hz), 135.4, 132.7, 131.6 (d, *J* = 4.0 Hz), 131.4, 130.6, 129.1 (d, *J* = 2.9 Hz), 128.6, 128.2, 128.0, 127.6 (d, *J* = 3.6 Hz), 125.7, 124.0 (d, *J* = 3.4 Hz), 123.9, 120.3, 118.5, 115.8, 115.5, 48.1, 18.3. ^{19}F NMR (376 MHz, $CDCl_3$) δ -116.51 (t, *J* = 9.8 Hz). HRMS (ESI): m/z $[M+H]^+$ calcd for $C_{25}H_{20}NOClF$: 404.1217; found: 404.1220.

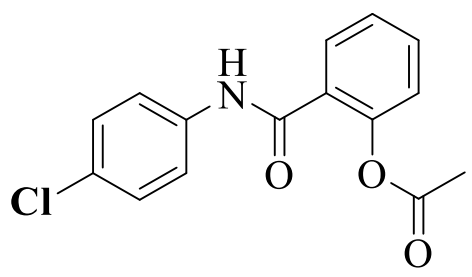

2-((4-chlorophenyl)carbamoyl)phenyl acetate (25)[1a] was synthesized using 37.8 mg of 2-acetoxybenzoic acid, 26.0 μL of $(COCl)_2$, 18.6 mg of aniline and following general procedure A using 5 mA constant current for 4 h. Compound **25** was isolated using automated flash chromatography (silica gel, gradient elution 5-30% EtOAc in isohexane) as an off-white solid (24.0 mg, 42% yield). ^{1}H NMR (400 MHz, $CDCl_3$) δ 8.07 (s, 1H), 7.81 (dd, *J* = 7.8, 1.7 Hz, 1H), 7.57 – 7.49 (m, 3H), 7.38 – 7.29 (m, 3H), 7.15 (dd, *J* = 8.1, 1.2 Hz, 1H), 2.32 (s, 3H). ^{13}C NMR (101 MHz, $CDCl_3$) δ 169.4, 163.8, 147.9, 136.5, 132.5, 130.0, 129.8, 129.3, 128.6, 126.7, 123.5, 121.2, 21.2.

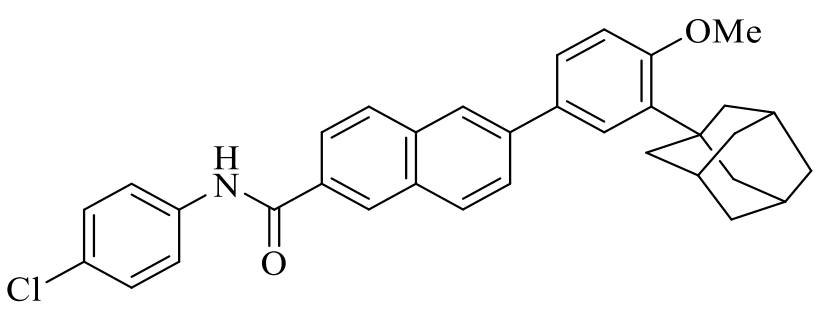

6-(3-((3r,5r,7r)-adamantan-1-yl)-4-methoxyphenyl)-*N*-(4-chlorophenyl)-2-naphthamide (26) was synthesized using 86.6 mg of 6-(3-((3r,5r,7r)-adamantan-1-yl)-4-methoxyphenyl)-2-naphthoic acid, 26.0 μL of $(COCl)_2$, 18.6 mg of aniline and following general procedure A using 5 mA constant current for 3 h. Compound **26** was isolated using automated flash chromatography (silica gel, gradient elution 5-30% EtOAc in isohexane) as a white solid (47 mg, 45% yield). ^{1}H NMR (400 MHz, $CDCl_3$) δ 8.49 (d, *J* = 8.9 Hz, 1H), 8.41 (d, *J* = 1.8 Hz, 1H), 8.03 (dd, *J* = 8.9, 1.9 Hz, 2H), 7.89 (d, *J* = 8.4 Hz, 1H), 7.74 – 7.69 (m, 2H), 7.57 (d, *J* = 8.4 Hz, 1H), 7.44 – 7.36 (m, 4H), 7.22 – 7.16 (m, 1H), 6.99 (d, *J* = 8.4 Hz, 1H), 3.92 (s, 3H), 2.16 (d, *J* = 3.0 Hz, 6H), 2.08 (t, *J* = 3.0 Hz, 3H), 1.79 (d, *J* = 3.2 Hz, 6H). ^{13}C

NMR (101 MHz, $CDCl_3$) δ 165.4, 158.7, 140.4, 138.3, 138.0, 133.1, 132.8, 132.6, 131.7, 130.3, 129.5, 129.3, 128.7, 128.2, 127.9, 127.8, 126.2, 124.9, 124.9, 120.4, 111.3, 55.2, 40.8, 37.3, 37.3, 29.2. HRMS (ESI): m/z $[M+H]^+$ calcd for $C_{34}H_{33}NO_2Cl$: 522.2200; found: 522.2200.

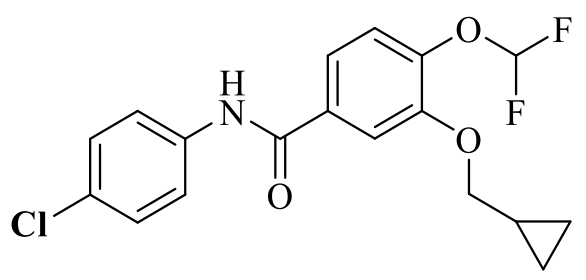

***N*-(4-chlorophenyl)-3-(cyclopropylmethoxy)-4-(difluoromethoxy)benzamide (27)** was synthesized using 54.2 mg of 3-(cyclopropylmethoxy)-4-(difluoromethoxy)benzoic acid, 26.0 μL of $(COCl)_2$, 18.6 mg of aniline and following general procedure A using 5 mA constant current for 5 h. Compound **27** was isolated using automated flash chromatography (silica gel, gradient elution 5-30% EtOAc in isohexane) as a white solid (71 mg, 97% yield). ^{1}H NMR (400 MHz, $CDCl_3$) δ 7.78 (s, 1H), 7.61 – 7.55 (m, 2H), 7.53 (d, *J* = 2.1 Hz, 1H), 7.36 – 7.29 (m, 3H), 7.23 (d, *J* = 8.3 Hz, 1H), 6.72 (t, *J* = 75.0 Hz, 1H), 3.94 (d, *J* = 6.9 Hz, 2H), 1.31 (dddd, *J* = 11.8, 8.2, 5.1, 2.2 Hz, 1H), 0.70 – 0.63 (m, 2H), 0.40 – 0.32 (m, 2H). ^{13}C NMR (101 MHz, $CDCl_3$) δ 164.8, 151.0, 143.3, 136.4, 133.1, 129.9, 129.3, 122.4, 121.6, 118.8, 117.2 (t, *J* = 261.2 Hz), 114.0, 74.3, 10.2, 3.4. ^{19}F NMR (376 MHz, $CDCl_3$) δ -81.97 (d, *J* = 74.9 Hz). HRMS (ESI): m/z $[M+H]^+$ calcd for $C_{18}H_{17}NO_3F_2Cl$: 368.0865; found: 368.0868.

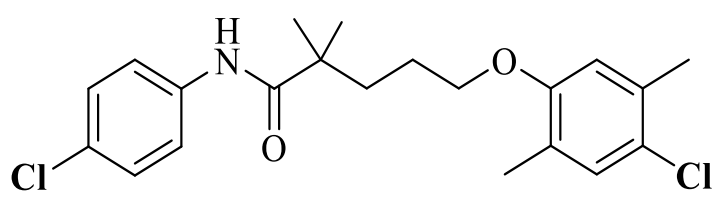

5-(4-chloro-2,5-dimethylphenoxy)-*N*-(4-chlorophenyl)-2,2-dimethylpentanamide (28) was synthesized using 52.5 mg of 5-(2,5-dimethylphenoxy)-2,2-dimethylpentanoic acid, 52.0 μL of $(COCl)_2$, 18.6 mg of aniline and following general procedure A using 5 mA constant current for 7 h. Compound **28** was isolated using automated flash chromatography (silica gel, gradient elution 10-30% EtOAc in isohexane) as a pale brown solid (61.3 mg, 78% yield). ^{1}H NMR (400 MHz, $CDCl_3$) 7.47 – 7.43 (m, 2H), 7.31 (s, 1H), 7.29 – 7.26 (m, 2H), 7.07 (s, 1H), 6.61 (s, 1H), 3.93 – 3.90 (m, 2H), 2.29 (s, 3H), 2.14 (s, 3H), 1.80 (d, *J* = 2.9 Hz, 4H), 1.33 (s, 6H). ^{13}C NMR (101 MHz, $CDCl_3$) δ 175.8, 155.5, 136.5, 133.9, 130.7, 129.4, 129.1, 125.9, 125.1, 121.4, 113.8, 68.3, 43.0, 37.7, 25.7, 25.2, 20.2, 15.7. HRMS (ESI): m/z $[M+H]^+$ calcd for $C_{21}H_{26}NO_2Cl_2$: 394.1341; found: 394.1349.

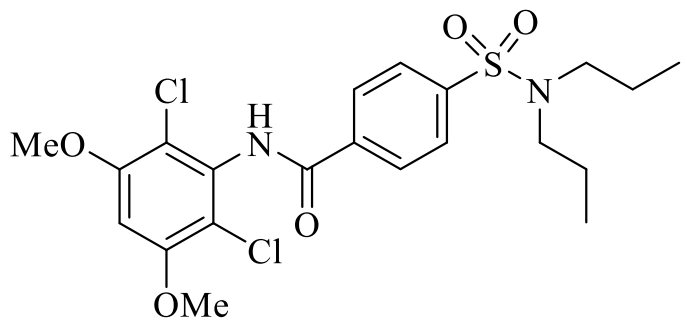

***N*-(2,6-dichloro-3,5-dimethoxyphenyl)-4-(*N*,*N*-dipropylsulfamoyl)benzamide (29)** was synthesized using 119.8 mg of 4-(*N*,*N*-dipropylsulfamoyl)benzoic acid, 52.0 μL of $(COCl)_2$, 30.6 mg of 3,5-dimethoxyaniline and following general procedure A using 15 mA constant current for 2.5 h.

Compound **29** was isolated using automated flash chromatography (silica gel, gradient elution 10-30% EtOAc in isohexane) as a brown solid (50.5 mg, 52% yield). ^{1}H NMR (400 MHz, DMSO-d$_6$) δ 10.51 (s, 1H), 8.15 (d, *J* = 8.3 Hz, 2H), 8.02 – 7.89 (m, 2H), 6.96 (s, 1H), 3.96 (s, 6H), 3.14 – 2.94 (m, 4H), 1.49 (q, *J* = 7.5 Hz, 4H), 0.82 (t, *J* = 7.4 Hz, 6H). ^{13}C NMR (101 MHz, DMSO-d$_6$) δ 164.0, 154.3, 142.3, 137.0, 134.0, 128.7, 127.1, 113.1, 97.4, 56.8, 49.7, 21.7, 11.0. HRMS (ESI): m/z [M+H]$^+$ calcd for $C_{21}H_{27}N_2O_5SCl_2$: 489.1018; found: 489.0995.

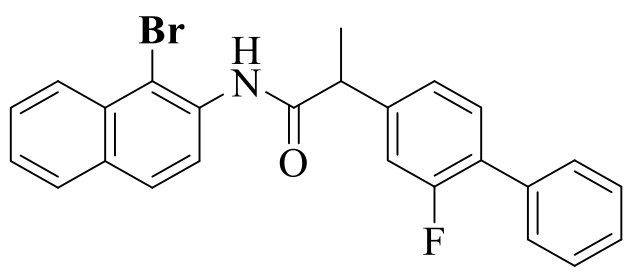

***N*-(1-bromonaphthalen-2-yl)-2-(2-fluoro-[1,1'-biphenyl]-4-yl)propanamide (30)** was synthesized using 51.2 mg of 2-(2-fluoro-[1,1'-biphenyl]-4-yl)propanoic acid, 158.0 μL of $(COBr)_2$ (2.0 M in DCM), 28.6 mg of naphthalen-2-amine and following general procedure B using 15 mA constant current for 8 h. Compound **30** was isolated using automated flash chromatography (silica gel, gradient elution 5-30% EtOAc in isohexane) as an off-white solid (56 mg, 63% yield). ^{1}H NMR (400 MHz, $CDCl_3$) δ 8.49 (d, *J* = 9.0 Hz, 1H), 8.09 (d, *J* = 8.6 Hz, 1H), 7.97 (s, 1H), 7.83 – 7.76 (m, 2H), 7.63 – 7.37 (m, 8H), 7.34 – 7.25 (m, 2H), 3.90 (q, *J* = 7.1 Hz, 1H), 1.73 (d, *J* = 7.2 Hz, 3H). ^{13}C NMR (101 MHz, $CDCl_3$) δ 172.0, 161.4, 158.9, 141.8 (d, *J* = 7.3 Hz), 135.4, 134.2, 132.0, 131.7, 131.6 (d, *J* = 4.0 Hz), 129.1 (d, *J* = 3.0 Hz), 128.6, 128.3, 128.0, 127.9 (d, *J* = 10.5 Hz), 126.6, 125.7, 124.1 (d, *J* = 4.0 Hz), 120.7, 115.9, 115.6, 111.8, 48.1, 18.2. ^{19}F NMR (376 MHz, $CDCl_3$) δ -116.51 (t, *J* = 9.8 Hz). HRMS (ESI): m/z [M+H]$^+$ calcd for $C_{25}H_{20}NOBrF$: 448.0712; found: 448.0727.

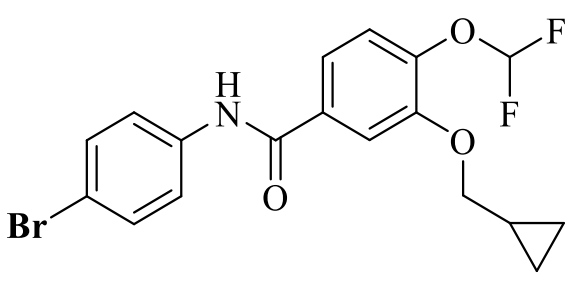

***N*-(4-bromophenyl)-3-(cyclopropylmethoxy)-4-(difluoromethoxy)benzamide (31)** was synthesized using 54.2 mg of 3-(cyclopropylmethoxy)-4-(difluoromethoxy)benzoic acid, 158.0 μL of $(COBr)_2$ (2.0 M in DCM), 18.6 mg of aniline and following general procedure B using 15 mA constant current for 26 h. Compound **31** was isolated using automated flash chromatography (silica gel, gradient elution 5-30% EtOAc in isohexane) as an off-white solid (39 mg, 48% yield). ^{1}H NMR (400 MHz, DMSO-d$_6$) δ 10.31 (s, 1H), 7.75 – 7.70 (m, 2H), 7.62 (d, *J* = 2.1 Hz, 1H), 7.58 – 7.51 (m, 3H), 7.31 (d, *J* = 8.3 Hz, 1H), 7.20 (t, *J* = 74.2 Hz, 1H), 3.98 (d, *J* = 7.0 Hz, 2H), 1.34 – 1.20 (m, 1H), 0.65 – 0.49 (m, 2H), 0.43 – 0.30 (m, 2H).^{13}C NMR (101 MHz, DMSO-d$_6$) δ 164.6, 149.5, 142.4 (t, *J* = 3.2 Hz), 138.4, 132.6, 131.5, 122.4, 120.4 (d, *J* = 26.7 Hz), 119.0, 116.5 (t, *J* = 258.6 Hz), 115.5, 113.7, 73.3, 9.9, 3.1. ^{19}F NMR (376 MHz, $CDCl_3$) δ -81.76 (d, *J* = 74.3 Hz). HRMS (ESI): m/z [M+H]$^+$ calcd for $C_{18}H_{17}NO_3BrF_2$: 412.0360; found: 412.0377.

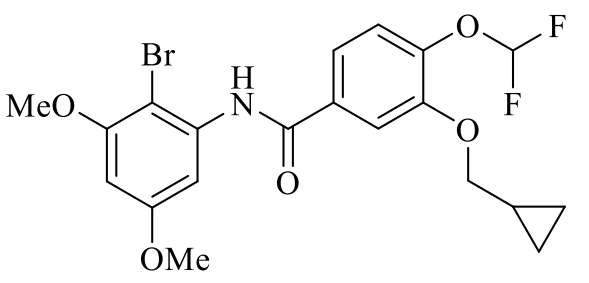

***N*-(2-bromo-3,5-dimethoxyphenyl)-3-(cyclopropylmethoxy)-4-(difluoromethoxy)benzamide (32)** was synthesized using 54.2 mg of 3-(cyclopropylmethoxy)-4-(difluoromethoxy)benzoic acid, 158.0 µL of $(COBr)_2$ (2.0 M in DCM), 30.6 mg of 3,5-dimethoxyaniline and following general procedure B using 15 mA constant current for 2 h. Compound **32** was isolated using automated flash chromatography (silica gel, gradient elution 5-30% EtOAc in isohexane) as an off-white solid (34.8 mg, 37% yield). ^{1}H NMR (400 MHz, $CDCl_3$) δ 8.61 (s, 1H), 7.94 (d, J = 2.7 Hz, 1H), 7.60 (d, J = 2.1 Hz, 1H), 7.43 (dd, J = 8.3, 2.1 Hz, 1H), 7.28 (d, J = 8.4 Hz, 1H), 6.73 (t, J = 74.9 Hz, 1H), 6.34 (d, J = 2.7 Hz, 1H), 3.97 (d, J = 7.0 Hz, 2H), 3.89 (s, 3H), 3.86 (s, 3H), 1.37 – 1.29 (m, 1H), 0.70 – 0.65 (m, 2H), 0.40 – 0.36 (m, 2H). ^{13}C NMR (101 MHz, $CDCl_3$) δ 164.5, 160.5, 156.6, 151.0, 143.5, 137.2, 133.1, 122.5, 119.0, 115.9 (t, J = 261.1 Hz), 114.0, 97.9, 96.3, 94.3, 74.3, 56.5, 55.9, 10.2, 3.4. ^{19}F NMR (376 MHz, $CDCl_3$) δ -81.93 (d, J = 74.8 Hz). HRMS (ESI): m/z $[M+H]^+$ calcd for $C_{20}H_{21}NO_5BrF_2$: 472.0571; found: 472.0584.

General procedure E: Scale-up

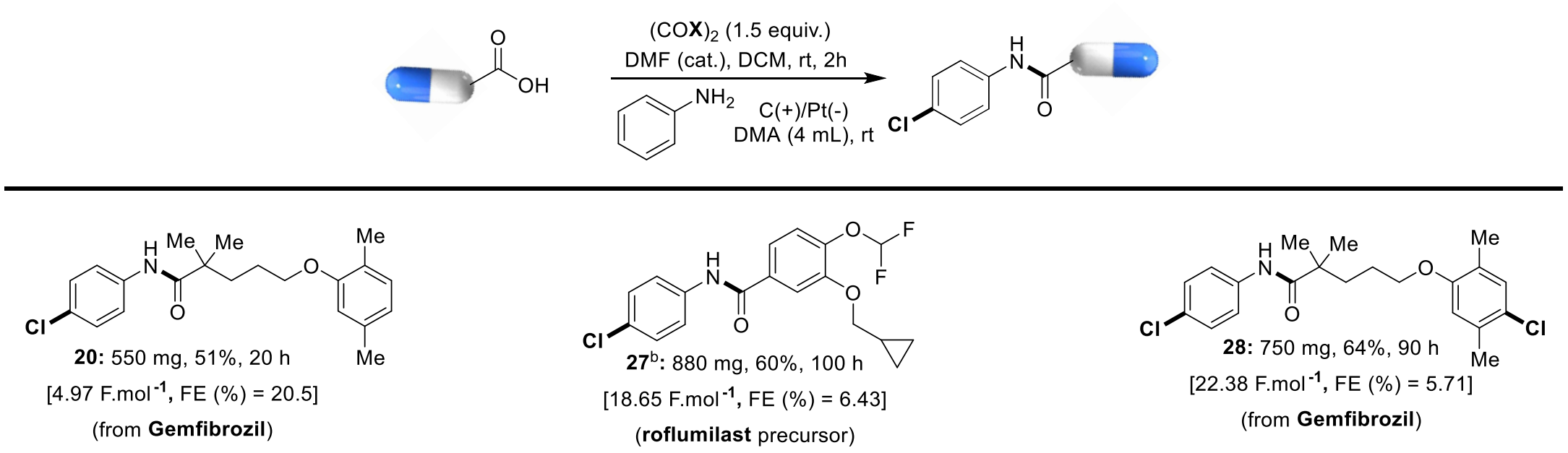

Reagents and conditions: [a] Carboxylic acid (6.3 mmol), $(COCl)_2$ (9.45 mmol), DCM (20 mL), DMF (cat.), rt, 2h, and aniline (3.0 mmol) in DMA (30 mL), 20 mA in an undivided cell with carbon cloth (anode and cathode); [b] Carboxylic acid (4.2 mmol), $(COCl)_2$ (6.3 mmol), DCM (20 mL), DMF (cat.), rt, 2h, and aniline (4.0 mmol) in DMA (30 mL), 20 mA in an undivided cell with carbon cloth (anode and cathode); All yields refer to isolated yields.

Procedure C: The electrolysis was carried out in 50 mL round bottom flask in an undivided cell setup under air. A carbon cloth anode (40 mm × 30 mm × 410 µm) and a carbon cloth cathode (40 mm × 30 mm × 410 µm) with electrode holders made of stainless steel were used and the distance between two electrodes was 20 mm. The cell was charged with the acid (1.05 equiv.) and a teflon-coated magnetic stirring bar (15 × 6 mm). DCM (20 mL) of was then added followed by few drops of DMF, and the reaction vessel was cooled down to 0 ºC. To this cooled and stirring solution, $(COCl)_2$ (1.5 equiv.) was added dropwise and the resulting reaction mixture was stirred at room temperature for 2 h. Upon completion of the reaction, DCM was removed completely under reduced pressure and DMA (20 mL) was added. Then, amine (1.00 equiv.) dissolved in DMA (10 mL) was added slowly to the stirring

reaction solution. Electrolysis was performed at room temperature with a constant current of 20.0 mA (unless otherwise stated) with a stirring rate of 600 rpm. After completion of the reaction, the reaction mixture was diluted with ethyl acetate (20 mL) and transferred to a round bottom flask. The electrodes (carbon cloth and platinum) were washed in the reaction flask with ethyl acetate (3 × 10 mL) in an ultrasonic cleaner (3 × 3 min) and the washes were combined in the round bottom flask. The reaction mixture was then transferred to a separating funnel and washed with brine (50 mL). The aqueous layer was washed with ethyl acetate (3 x 50 mL) and the combined organic layer was washed with brine (5 x 100 mL). The organic layer was dried with sodium sulfate and the solvent was removed under vacuum to obtain crude product. The crude products were purified by flash column chromatography using isohexane and ethyl acetate mixture (20:1 to 0:100) to afford the title compounds.

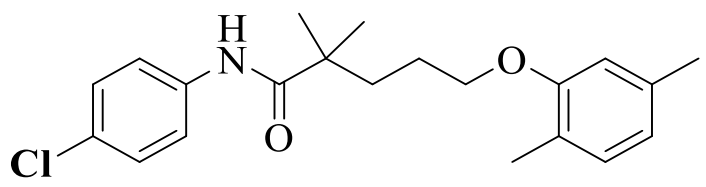

***N*-(4-chlorophenyl)-5-(2,5-dimethylphenoxy)-2,2-dimethylpentanamide (20)** was synthesized using 5-(2,5-dimethylphenoxy)-2,2-dimethylpentanoic acid (1.575 g, 6.3 mmol, 2.1 equiv.), $(COCl)_2$ (810.0 μL, 9.45 mmol, 3.0 equiv.), aniline (279 mg, 3.0 mmol, 1.0 equiv.) and following general procedure C using 20 mA constant current for 20 h. Compound **20** was isolated using automated flash chromatography (silica gel, gradient elution 5-30% EtOAc in isohexane) as a white solid (550 mg, 51% yield). ^{1}H NMR (400 MHz, $CDCl_3$) δ 7.51 – 7.45 (m, 2H), 7.40 (s, 1H), 7.32 – 7.28 (m, 2H), 7.03 (d, *J* = 7.5 Hz, 1H), 6.69 (dd, *J* = 7.6, 1.7 Hz, 1H), 6.63 (d, *J* = 1.7 Hz, 1H), 3.97 (p, *J* = 2.5 Hz, 2H), 2.32 (s, 3H), 2.20 (s, 3H), 1.85 (q, *J* = 3.0, 2.6 Hz, 4H), 1.36 (s, 6H). ^{13}C NMR (101 MHz, $CDCl_3$) δ 175.9, 156.9, 136.7, 136.6, 130.5, 129.4, 129.0, 123.6, 121.5, 121.0, 112.3, 67.9, 43.0, 37.7, 25.7, 25.2, 21.5, 16.0.

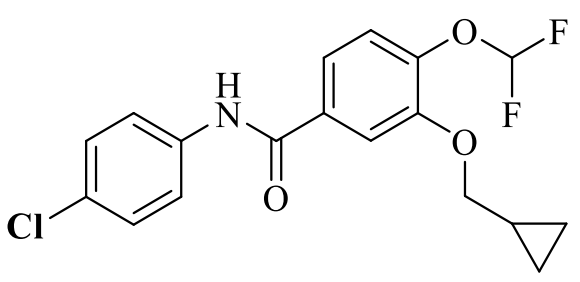

***N*-(4-chlorophenyl)-3-(cyclopropylmethoxy)-4-(difluoromethoxy)benzamide (27)** was synthesized using 3-(cyclopropylmethoxy)-4-(difluoromethoxy)benzoic acid (1.84 g, 4.2 mmol, 1.05 equiv.), $(COCl)_2$ (546.0 μL, 6.3 mmol, 1.5 equiv.), aniline (373 mg, 4.0 mmol, 1.0 equiv.) and following general procedure C using 20 mA constant current for 100 h. Compound **27** was isolated using automated flash chromatography (silica gel, gradient elution 5-30% EtOAc in isohexane) as a white solid (880 mg, 60% yield). ^{1}H NMR (400 MHz, $CDCl_3$) δ 7.78 (s, 1H), 7.61 – 7.55 (m, 2H), 7.53 (d, *J* = 2.1 Hz, 1H), 7.36 – 7.29 (m, 3H), 7.23 (d, *J* = 8.3 Hz, 1H), 6.72 (t, *J* = 75.0 Hz, 1H), 3.94 (d, *J* = 6.9 Hz, 2H), 1.31 (dddd, *J* = 11.8, 8.2, 5.1, 2.2 Hz, 1H), 0.70 – 0.63 (m, 2H), 0.40 – 0.32 (m, 2H). ^{13}C NMR (101 MHz, $CDCl_3$) δ 164.8, 151.0, 143.3, 136.4, 133.1, 129.9, 129.3, 122.4, 121.6, 118.8, 117.2 (t, *J* = 261.2 Hz), 114.0,

74.3, 10.2, 3.4. ^{19}F NMR (376 MHz, $CDCl_3$) δ -81.97 (d, J = 74.9 Hz). HRMS (ESI): m/z $[M+H]^+$ calcd for $C_{18}H_{17}NO_3F_2Cl$: 368.0865; found: 368.0868.

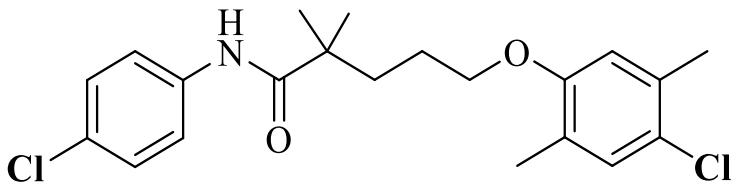

5-(4-chloro-2,5-dimethylphenoxy)-*N*-(4-chlorophenyl)-2,2-dimethylpentanamide (28) was synthesized using 5-(2,5-dimethylphenoxy)-2,2-dimethylpentanoic acid (1.575 g, 6.3 mmol, 2.1 equiv.), $(COCl)_2$ (810.0 μL, 9.45 mmol, 3.0 equiv.), aniline (279 mg, 3.0 mmol, 1.0 equiv.) and following general procedure C using 20 mA constant current for 90 h. Compound **28** was isolated using automated flash chromatography (silica gel, gradient elution 5-30% EtOAc in isohexane) as a white solid (750 mg, 64% yield). ^{1}H NMR (400 MHz, $CDCl_3$) δ 7.47 – 7.43 (m, 2H), 7.31 (s, 1H), 7.29 – 7.26 (m, 2H), 7.07 (s, 1H), 6.61 (s, 1H), 3.93 – 3.90 (m, 2H), 2.29 (s, 3H), 2.14 (s, 3H), 1.80 (d, J = 2.9 Hz, 4H), 1.33 (s, 6H). ^{13}C NMR (101 MHz, $CDCl_3$) δ 175.8, 155.5, 136.5, 133.9, 130.7, 129.4, 129.1, 125.9, 125.1, 121.4, 113.8, 68.3, 43.0, 37.7, 25.7, 25.2, 20.2, 15.7. HRMS (ESI): m/z $[M+H]^+$ calcd for $C_{21}H_{26}NO_2Cl_2$: 394.1341; found: 394.1349.

List of reported compounds:

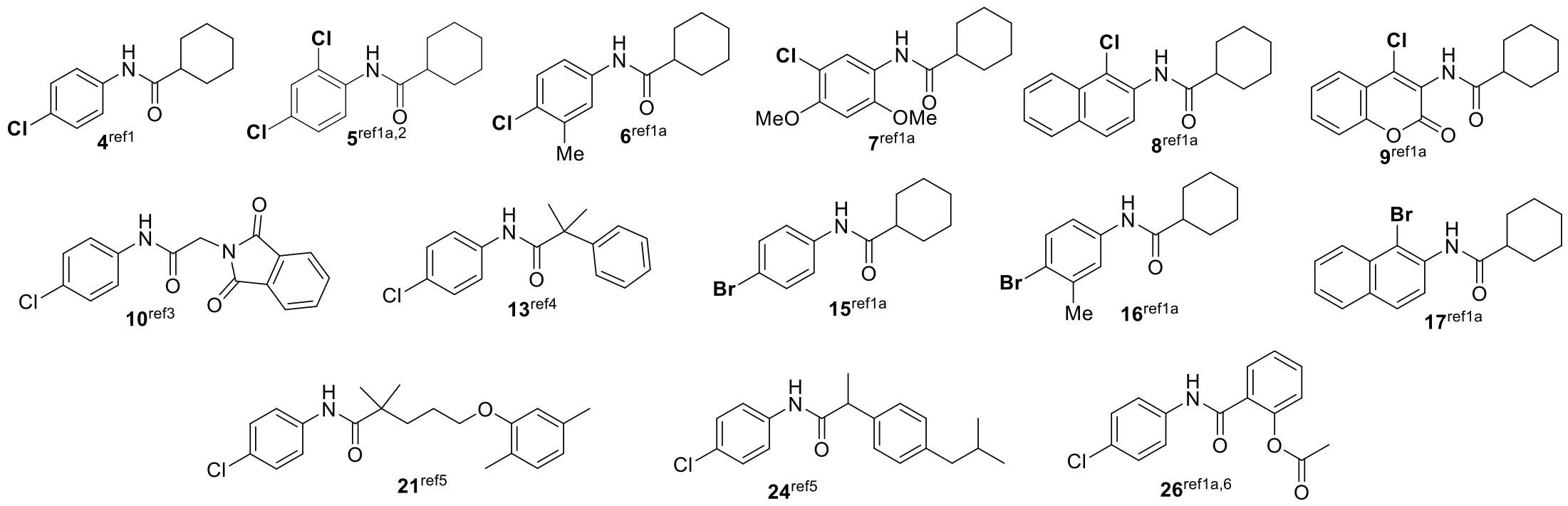

References

1. (a) S. Ponra, R. Sitdikov, H. Aman, A. Calis, G. Laczkó, V. Rouffeteau, M. R. Vitale, I. Pápai and O. Verho, *Angew. Chem. Int. Ed.*, **2026**, DOI: 10.1002/anie.9028210. (b) L. Lu, F. Qiu, H. Alhumade, H. Zhang and A. Lei, *ACS Catal.*, **2022**, *12*, 9664–9669.

2. Q. Yan, Q.-J. Yuan, A. Shatskiy, G. R. Alvey, E. V. Stepanova, J.-Q. Liu, M. D. Kärkäs and X.-S. Wang, *Org. Lett.*, **2024**, *26*, 3380–3385.

3. H.-Y. Zhang, X.-W. Tao, L.-N. Yi, Z.-G. Zhao and Q. Yang, *J. Org. Chem.*, **2022**, *87*, 231–242.

4. H.-Y. Yang, Y.-H. Yao, M. Chen, Z.-H. Ren and Z.-H. Guan, *J. Am. Chem. Soc.*, **2021**, *143*, 7298–7305.

5. S.-P. Wang, C. W. Cheung and J.-A. Ma, *J. Org. Chem.*, **2019**, *84*, 13922–13934.

6. M. K. Dahlgren, A. M. Kauppi, I.-M. Olsson, A. Linusson and M. Elofsson, *J. Med. Chem.*, **2007**, *50*, 6177–6188.

NMR spectra

***N*-(4-chlorophenyl)cyclohexanecarboxamide: (4)**

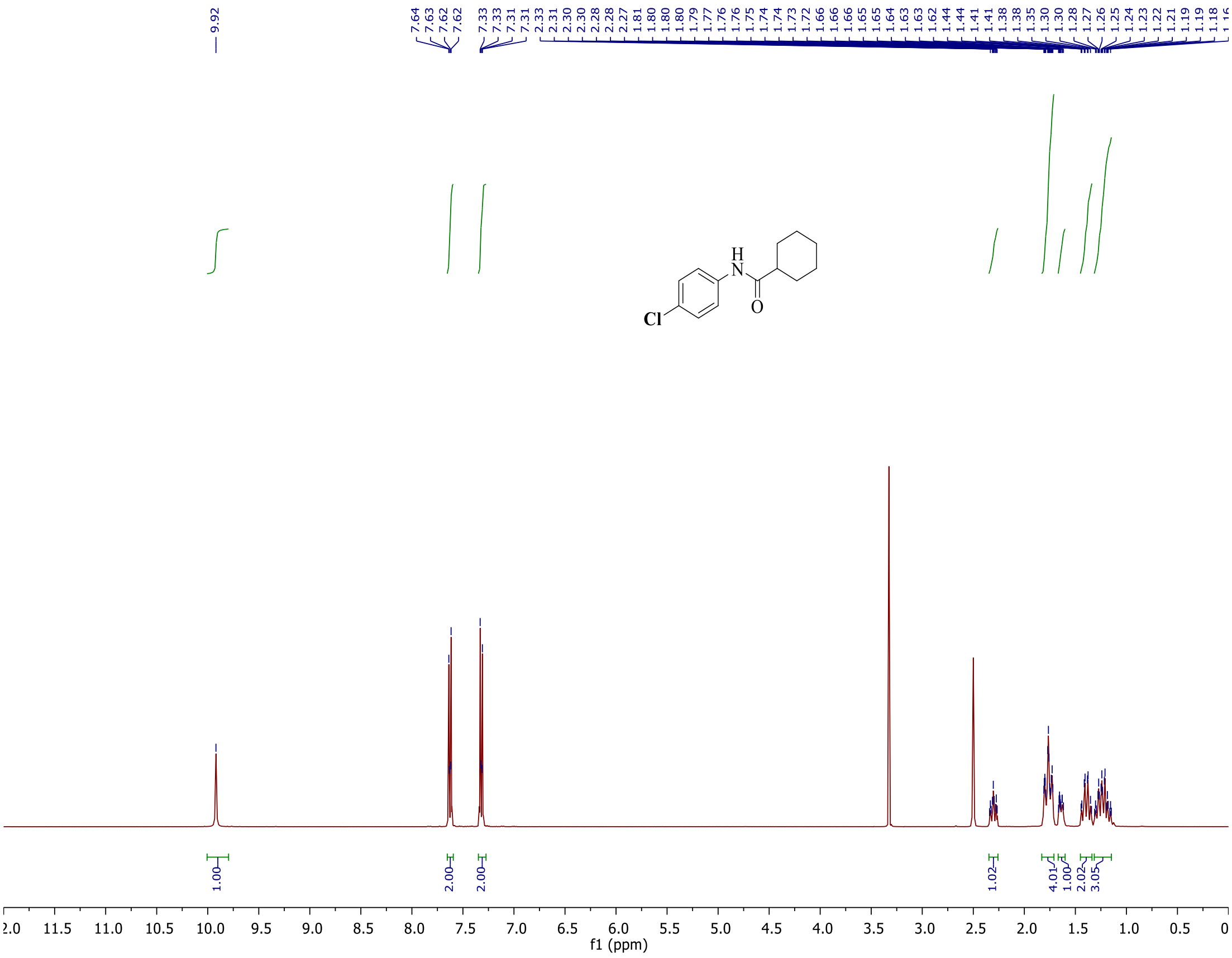

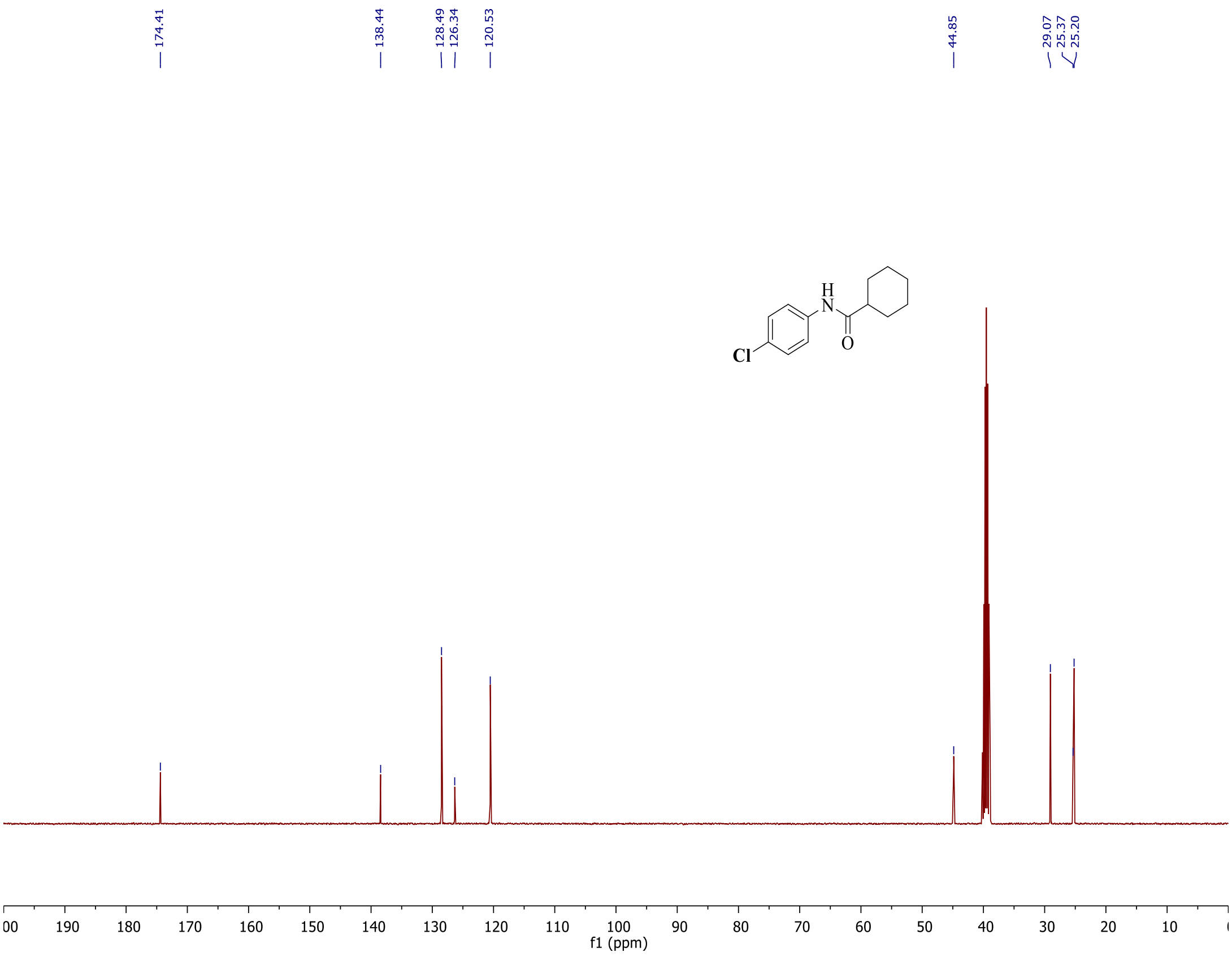

***N*-(2,4-dichlorophenyl)cyclohexanecarboxamide: (5)**

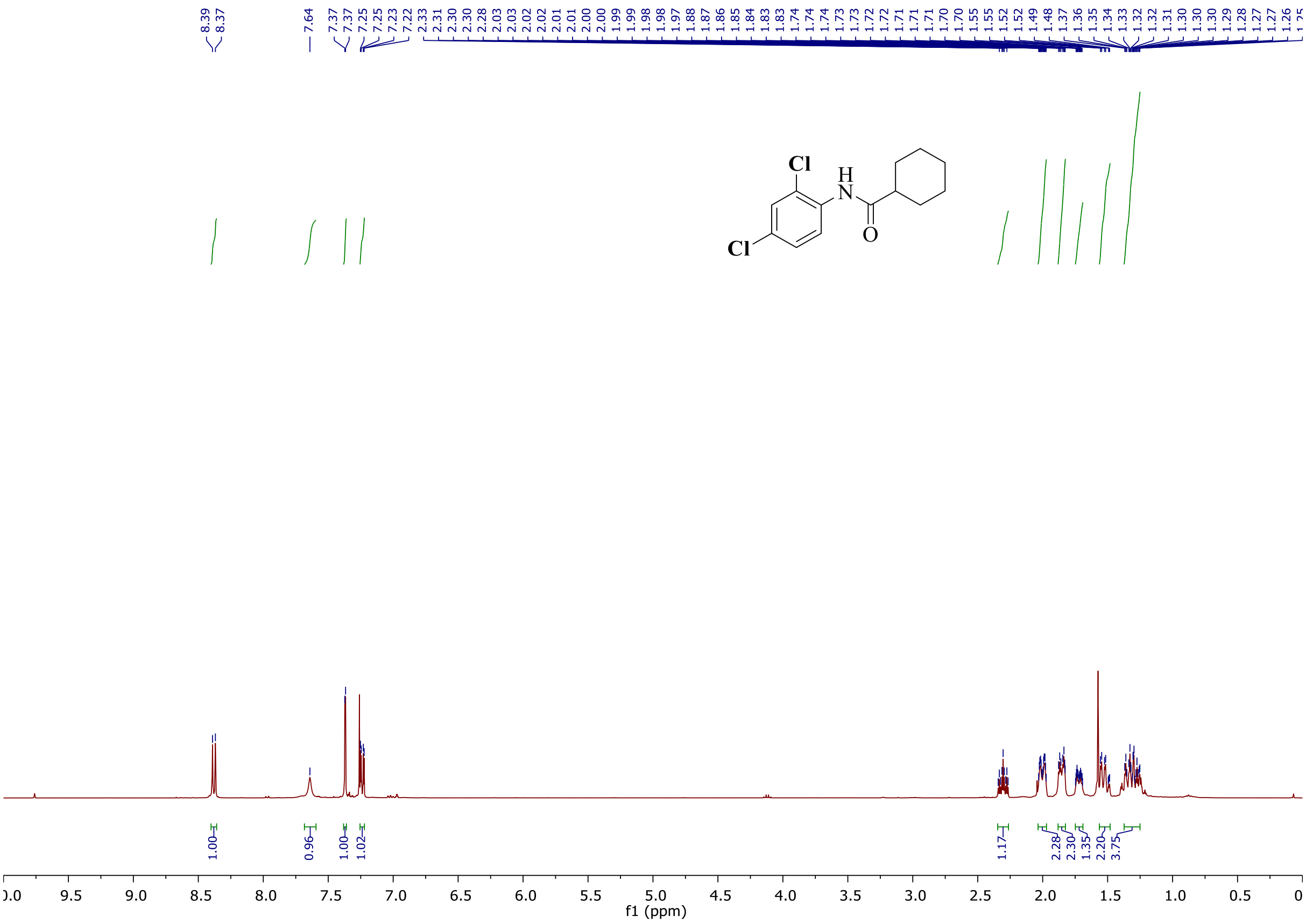

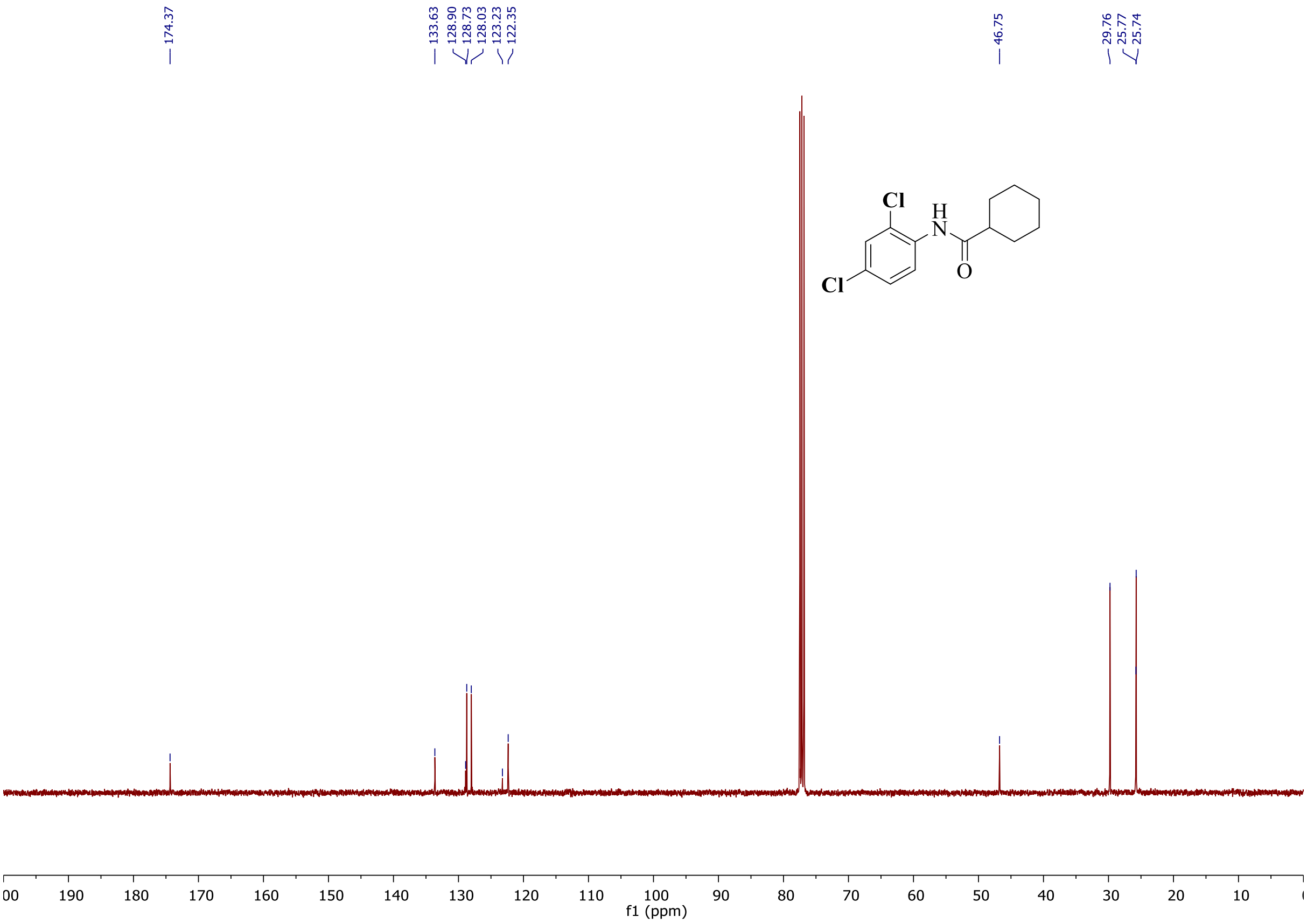

***N*-(4-chloro-3-methylphenyl)cyclohexanecarboxamide: (6)**

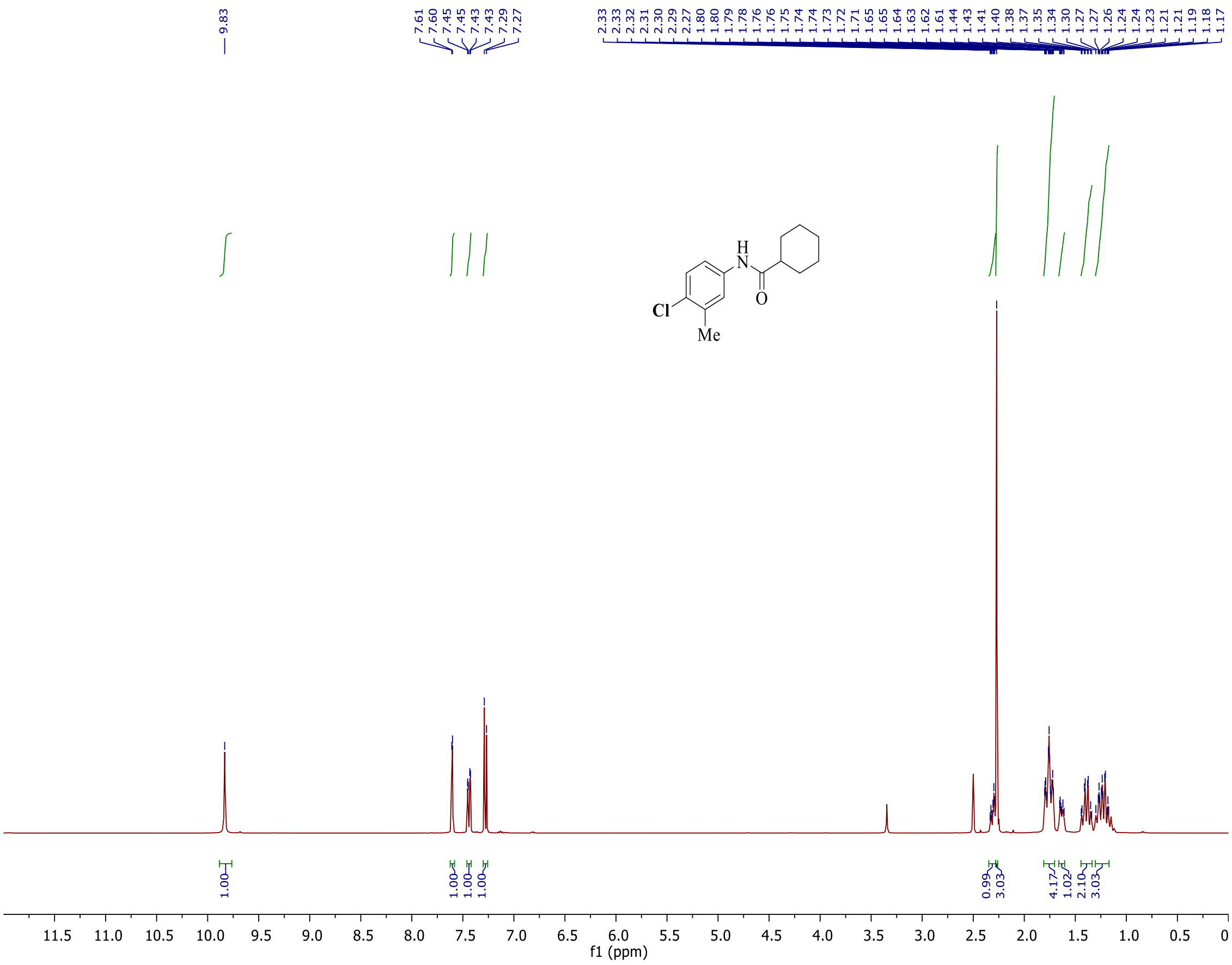

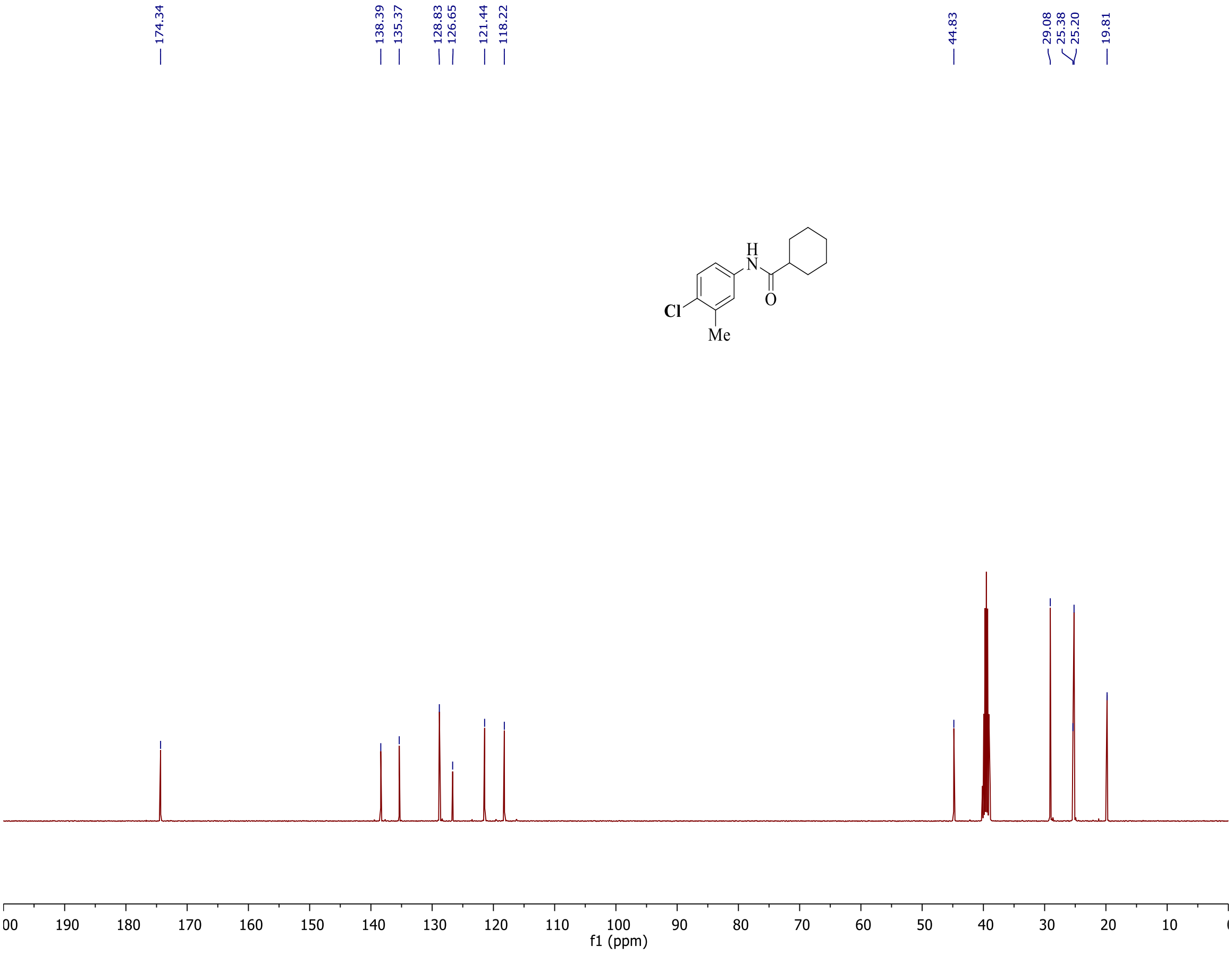

_N_-(5-chloro-2,4-dimethoxyphenyl)cyclohexanecarboxamide: (7)

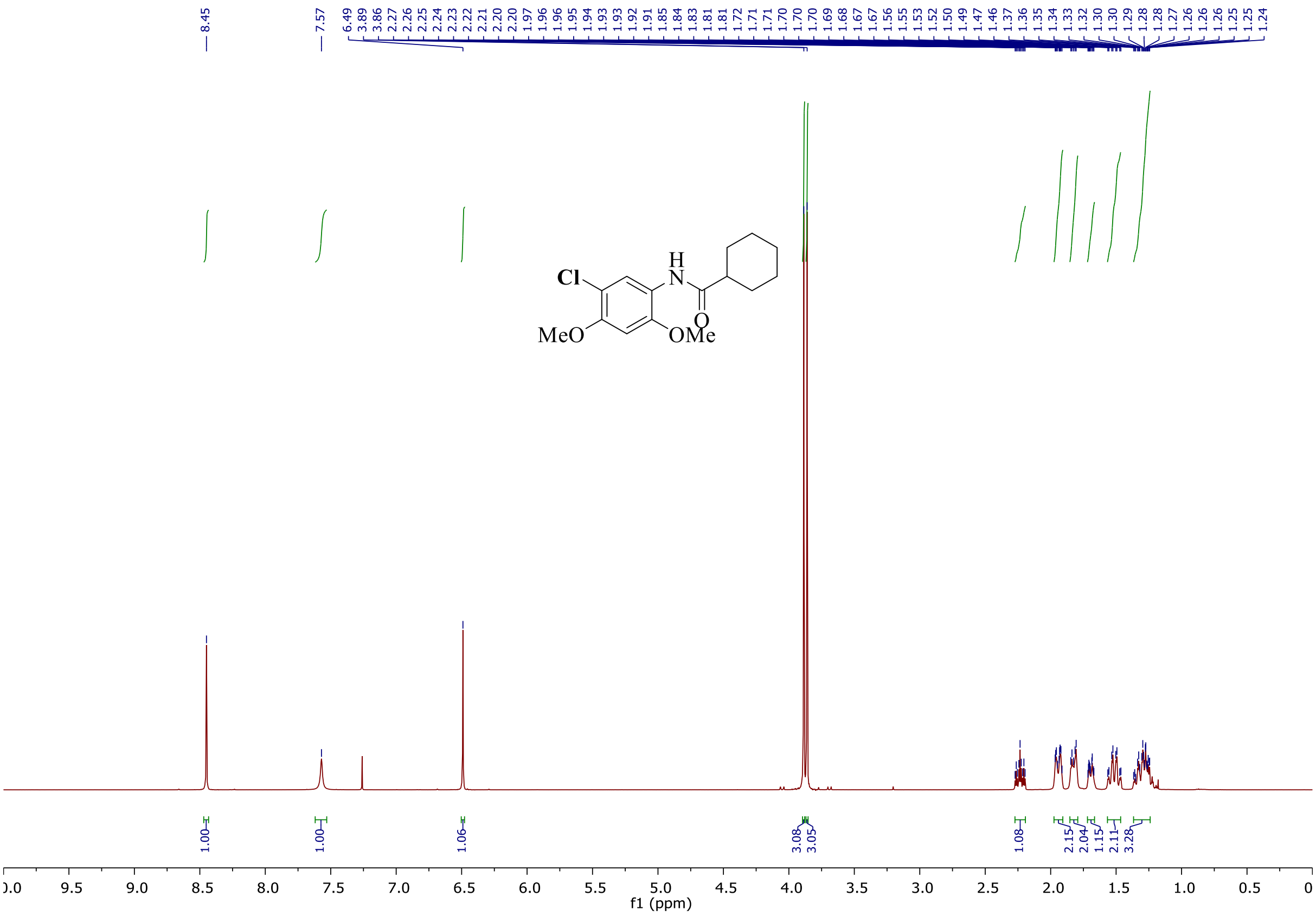

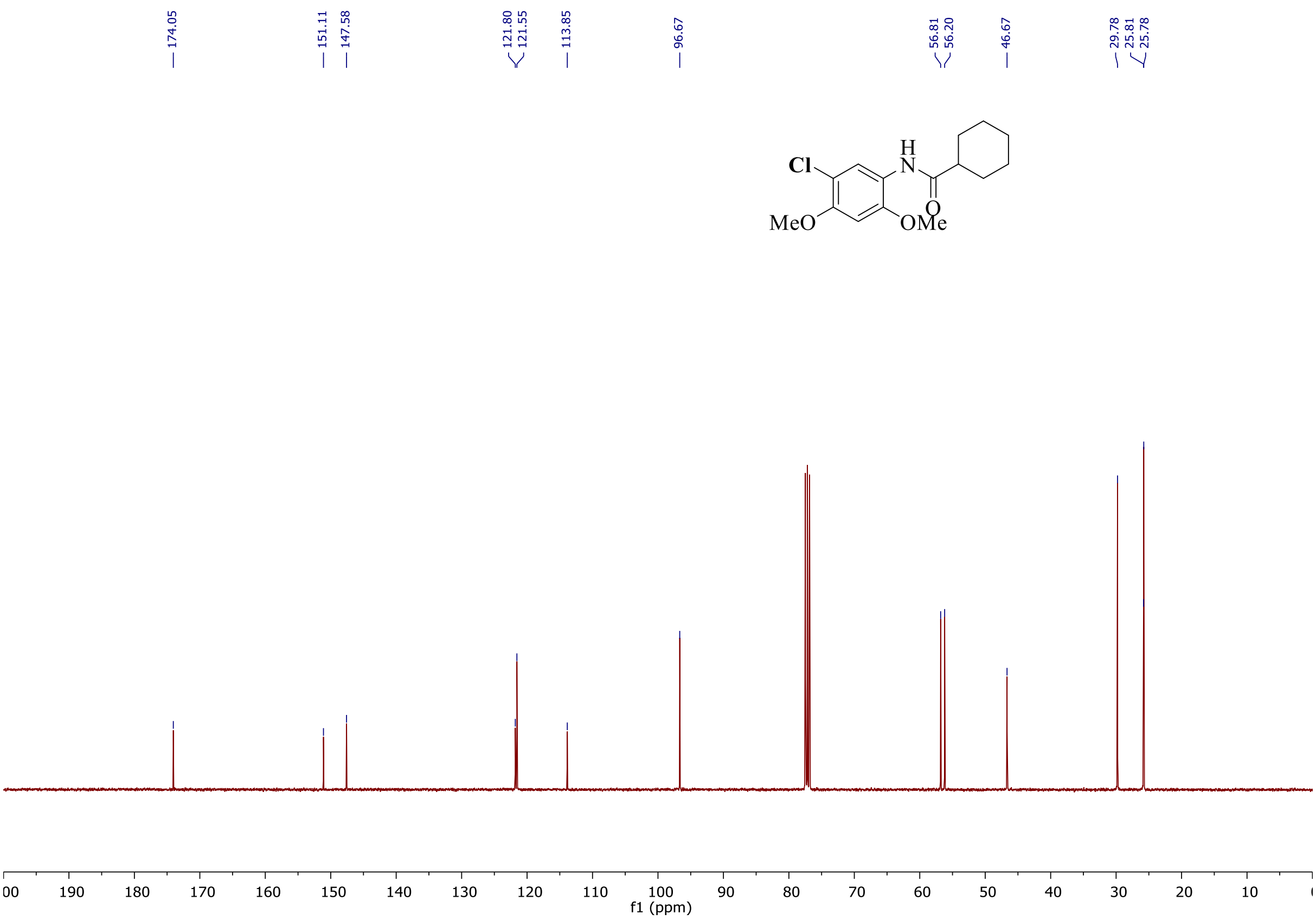

N-(1-chloronaphthalen-2-yl)cyclohexanecarboxamide: (8)

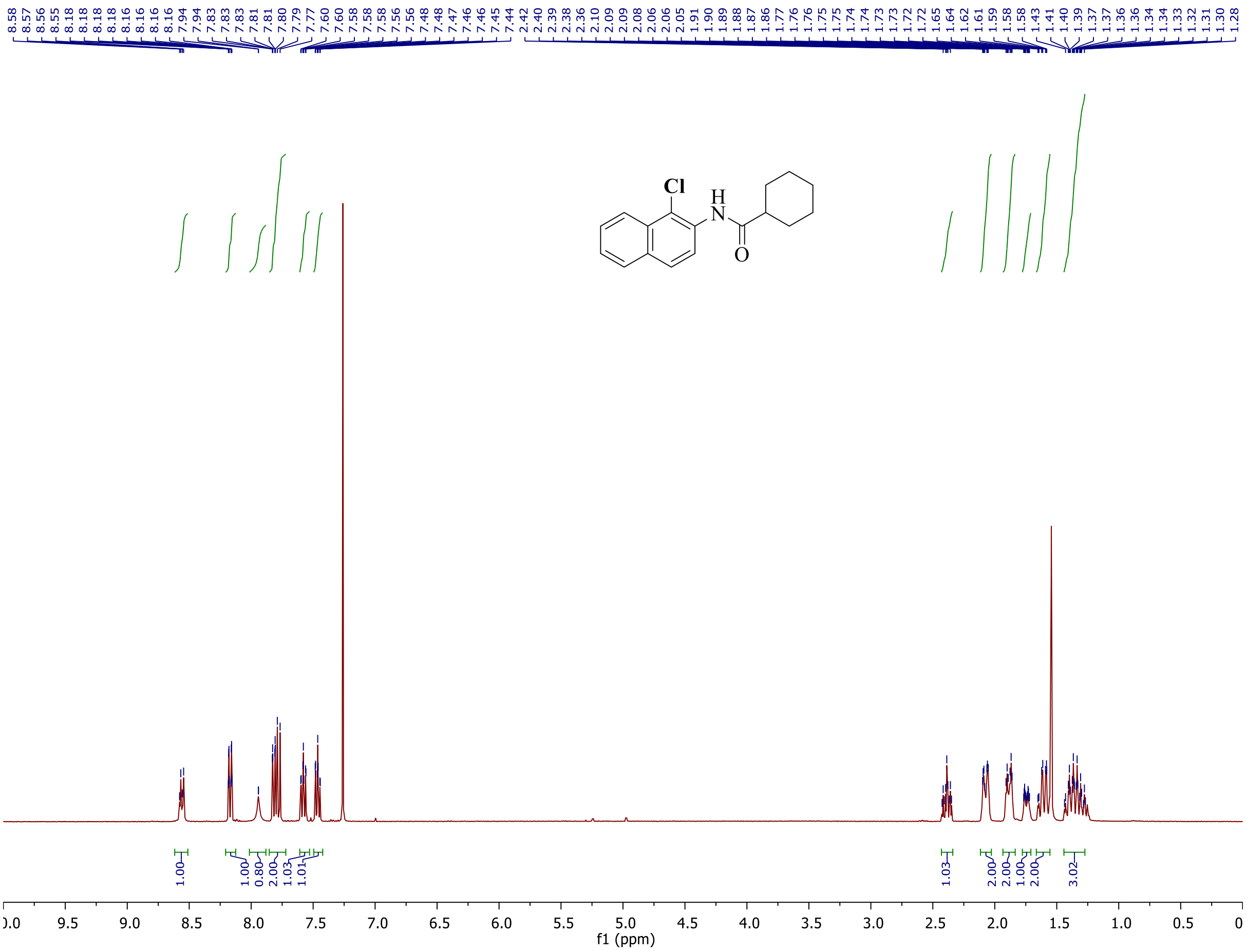

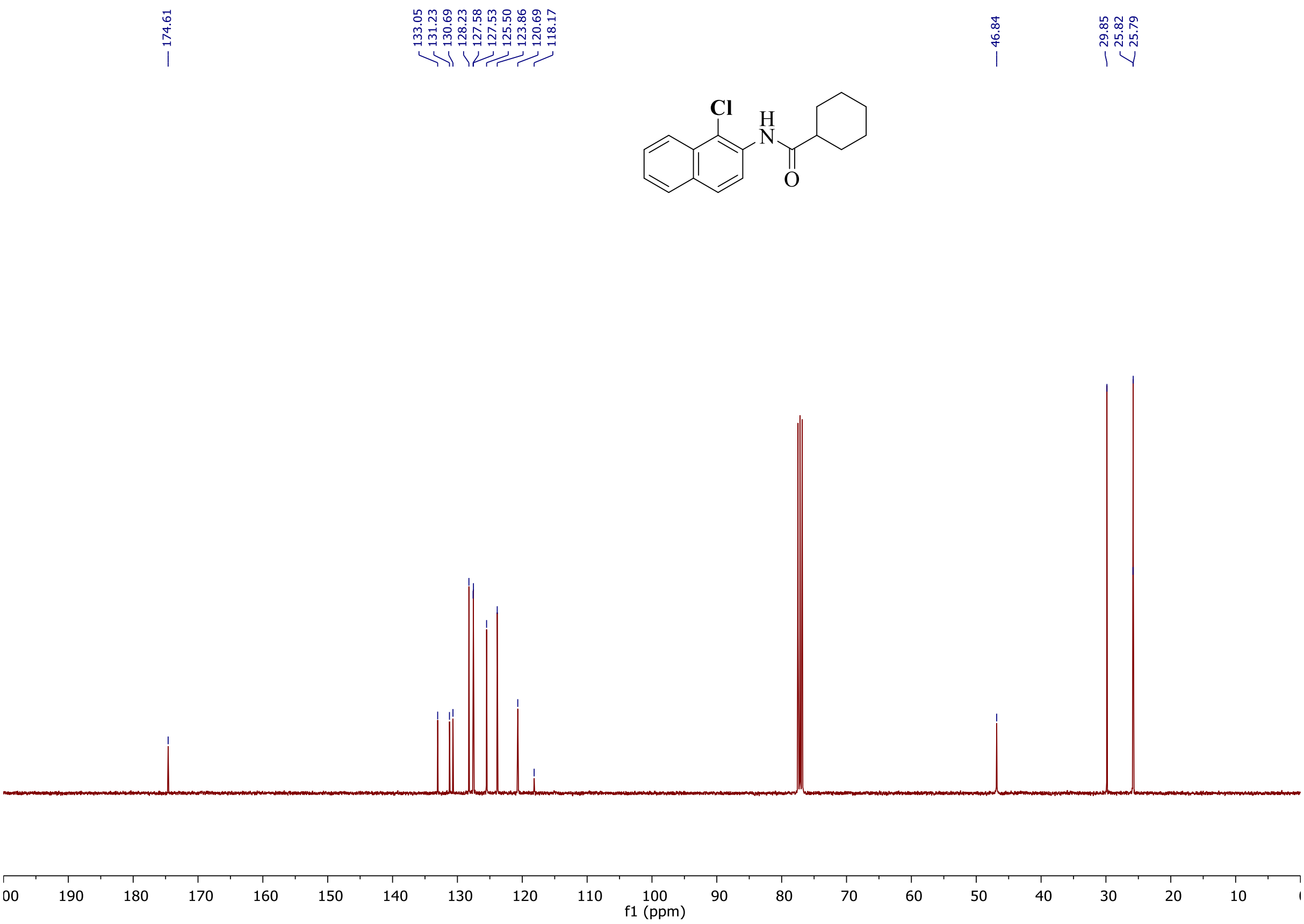

***N*-(4-chloro-2-oxo-2H-chromen-3-yl)cyclohexanecarboxamide: (9)**

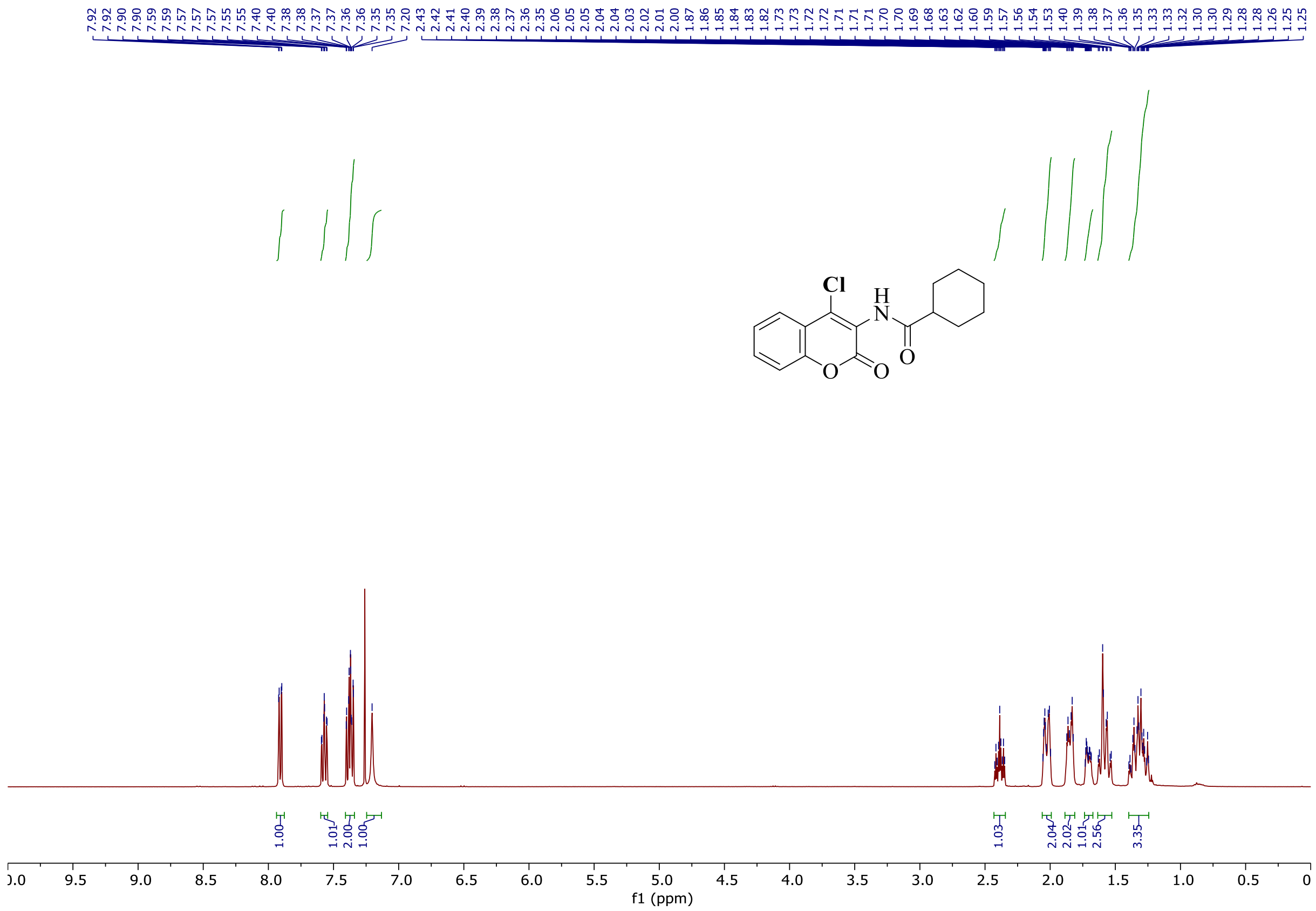

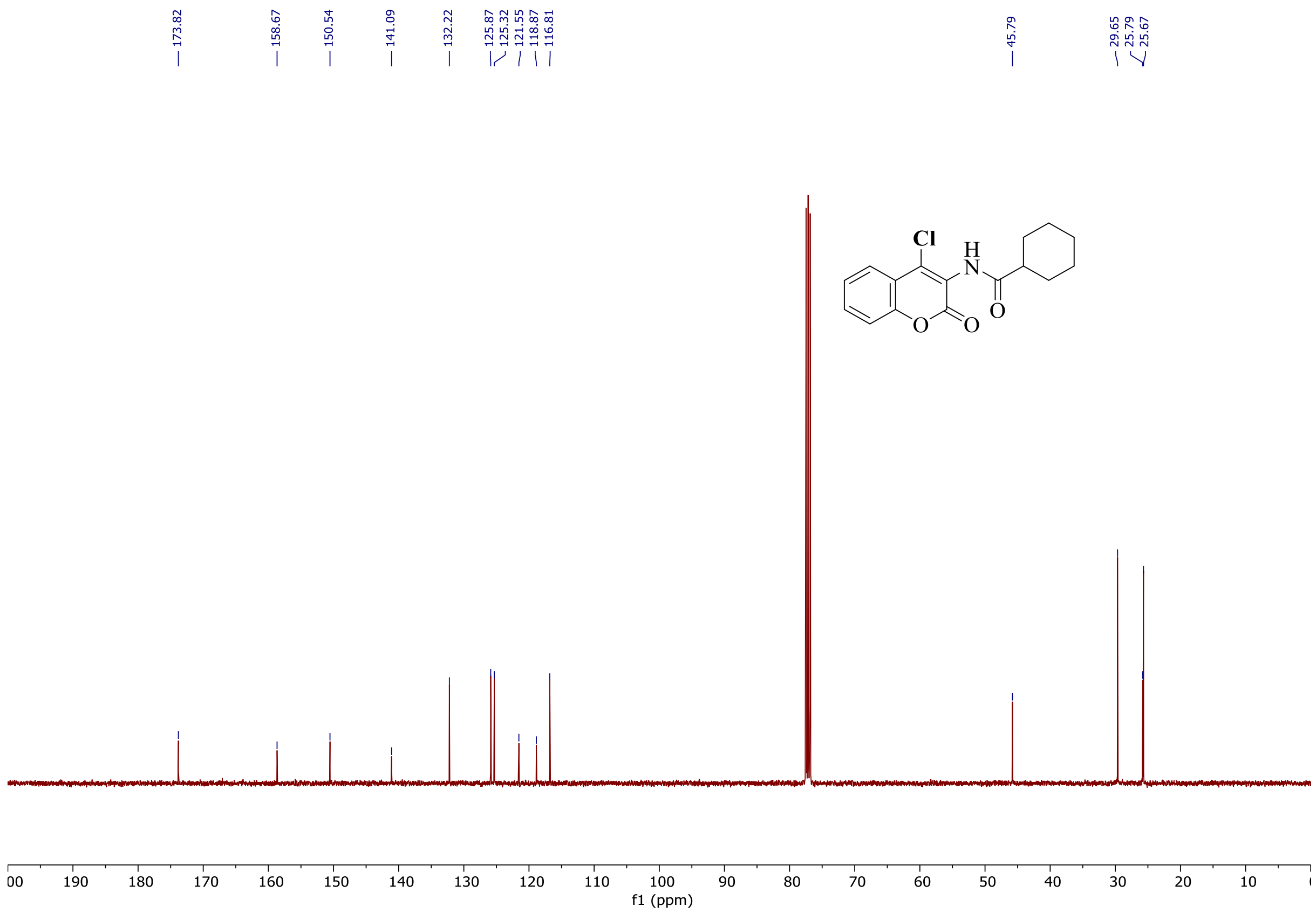

N-(4-chlorophenyl)-2-(1,3-dioxoisoindolin-2-yl)acetamide: (10)

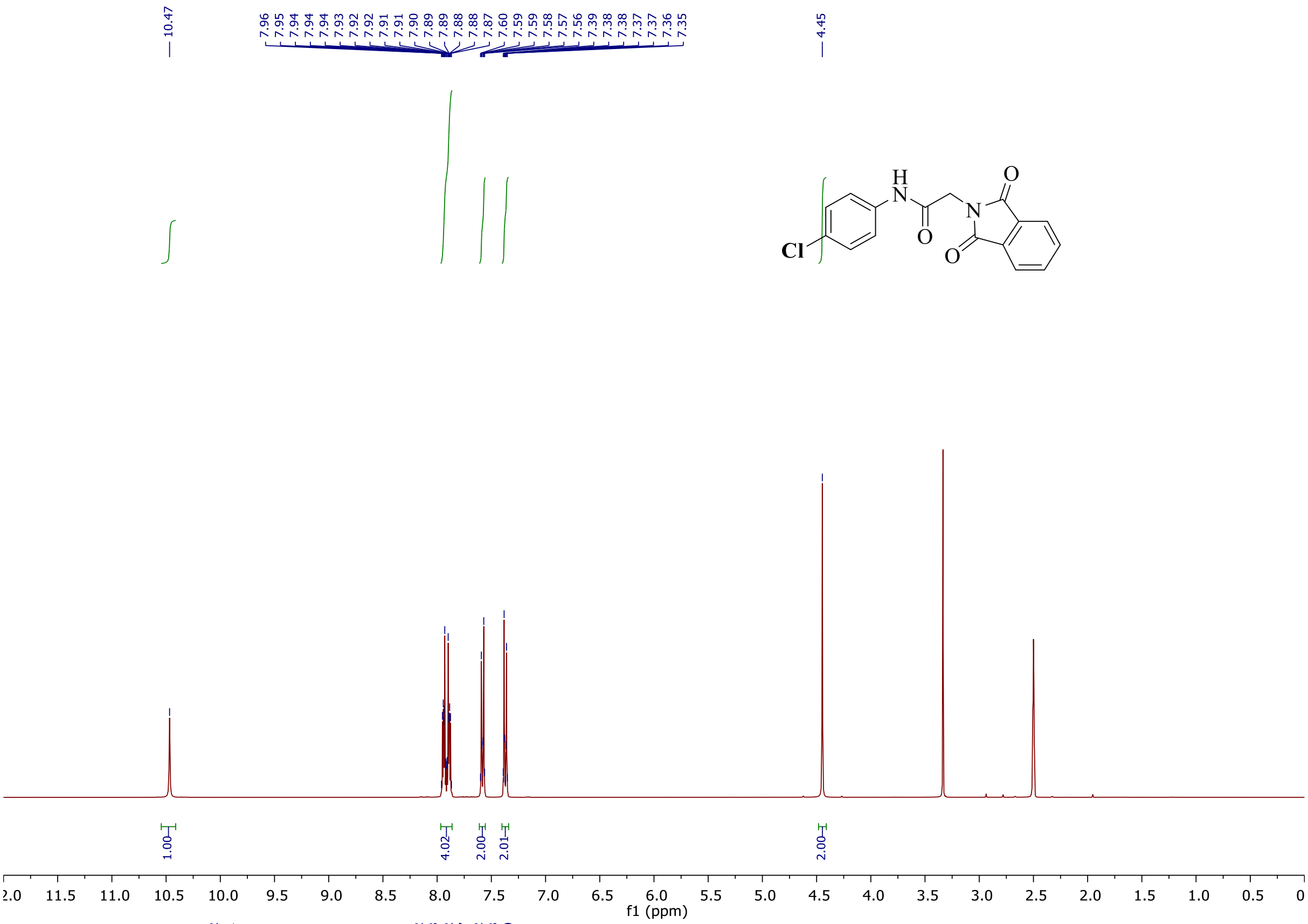

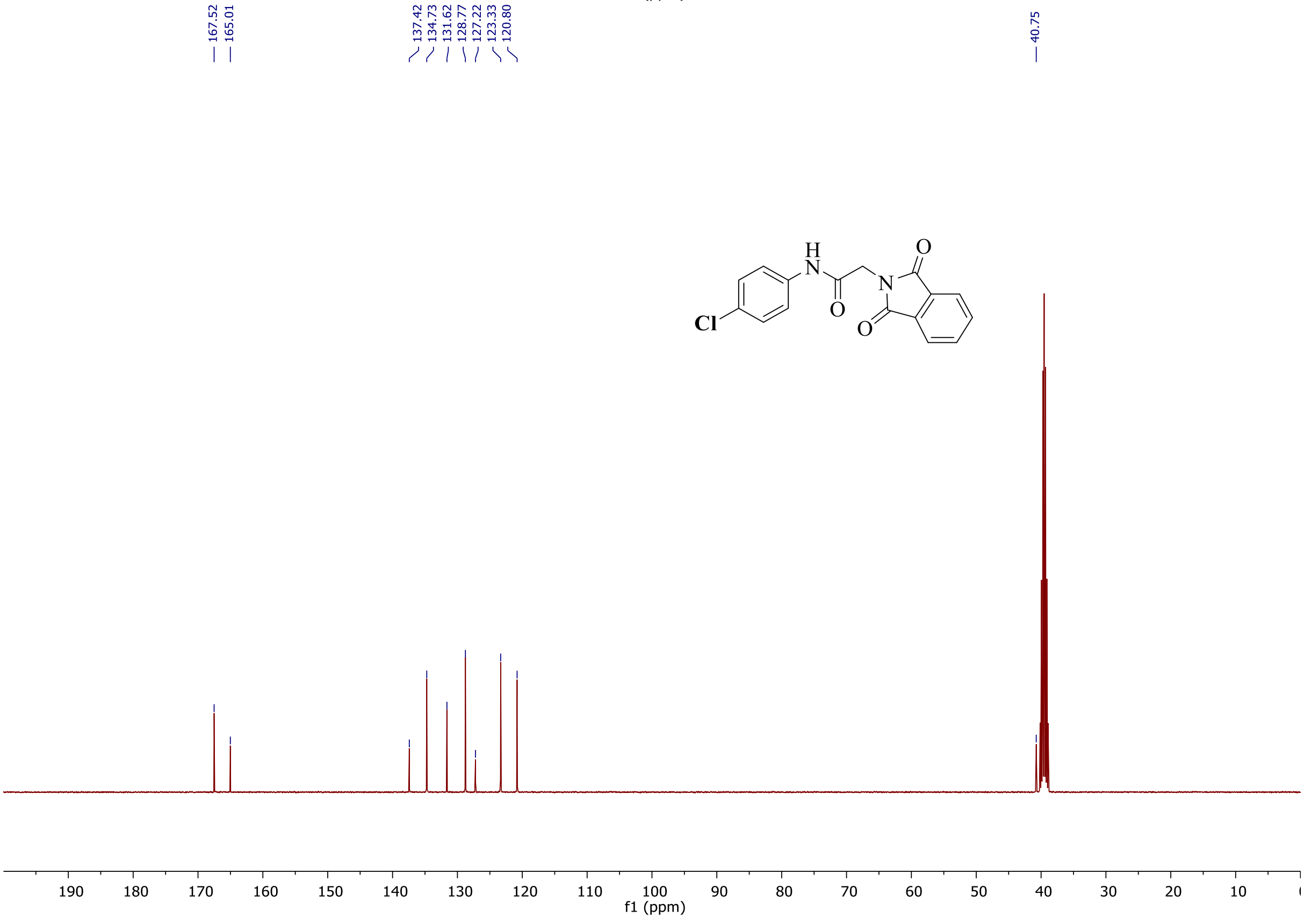

***N*-(4-chlorophenyl)quinoline-2-carboxamide: (11)**

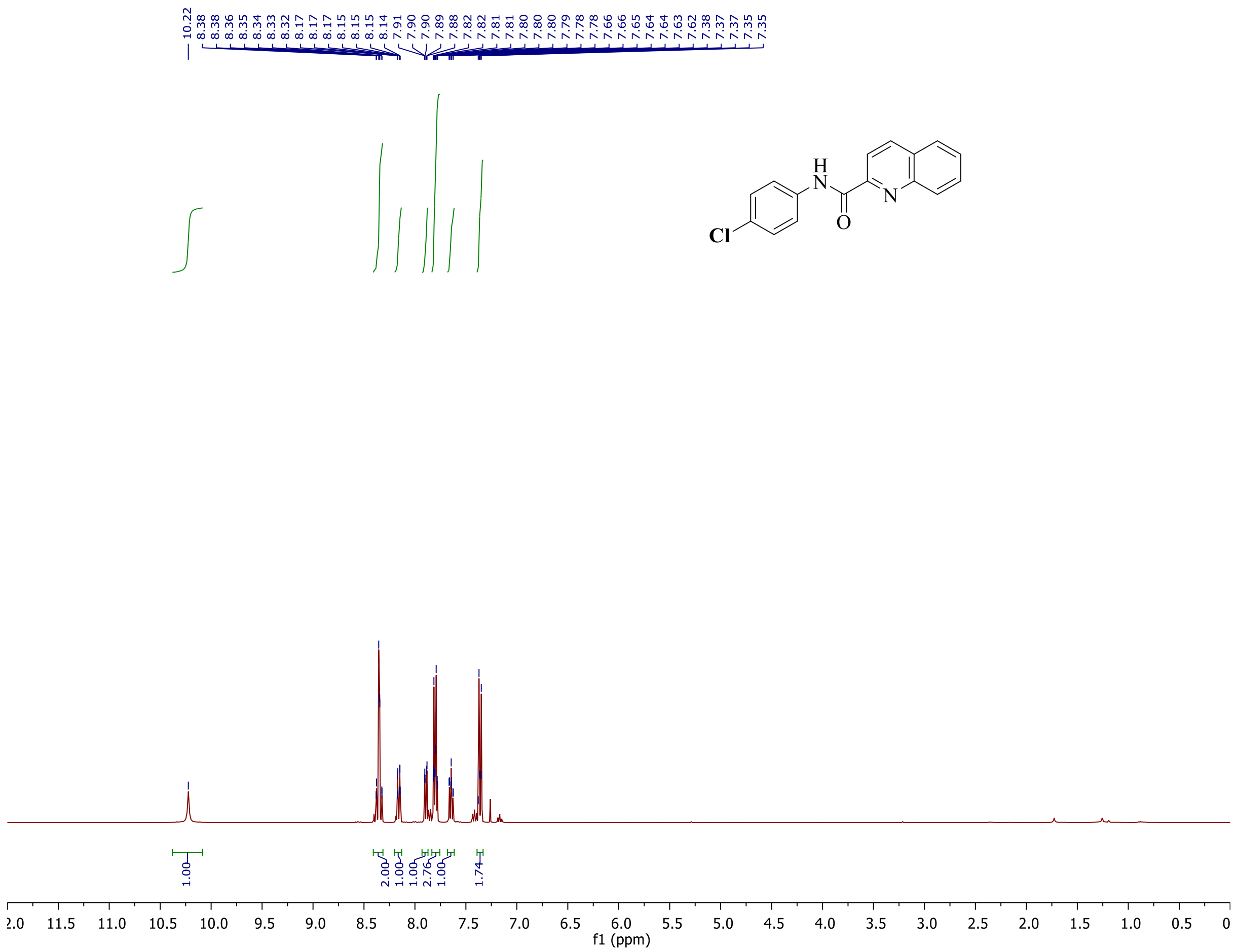

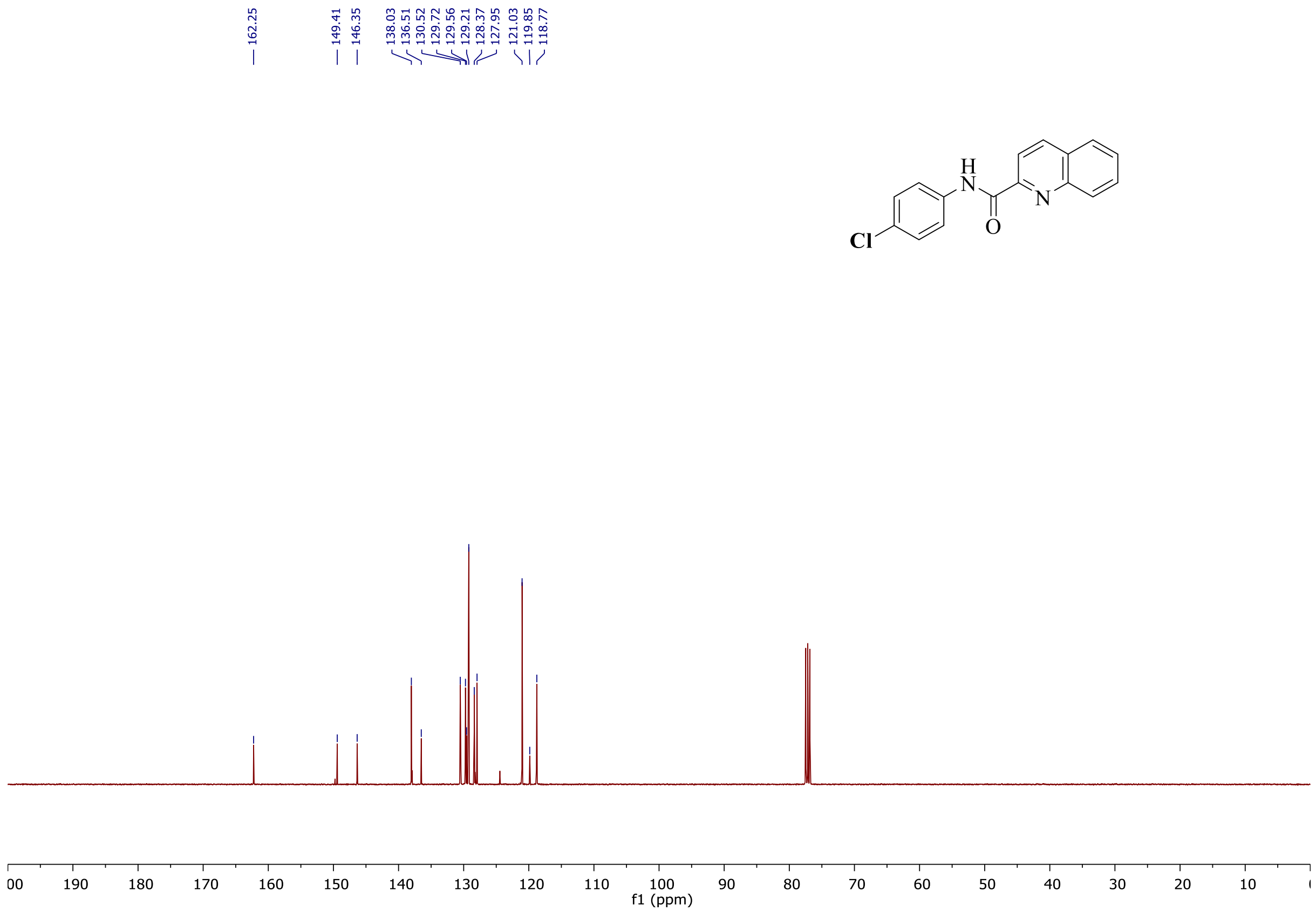

N-(4-chlorophenyl)-3-methylbenzo[b]thiophene-2-carboxamide: (12)

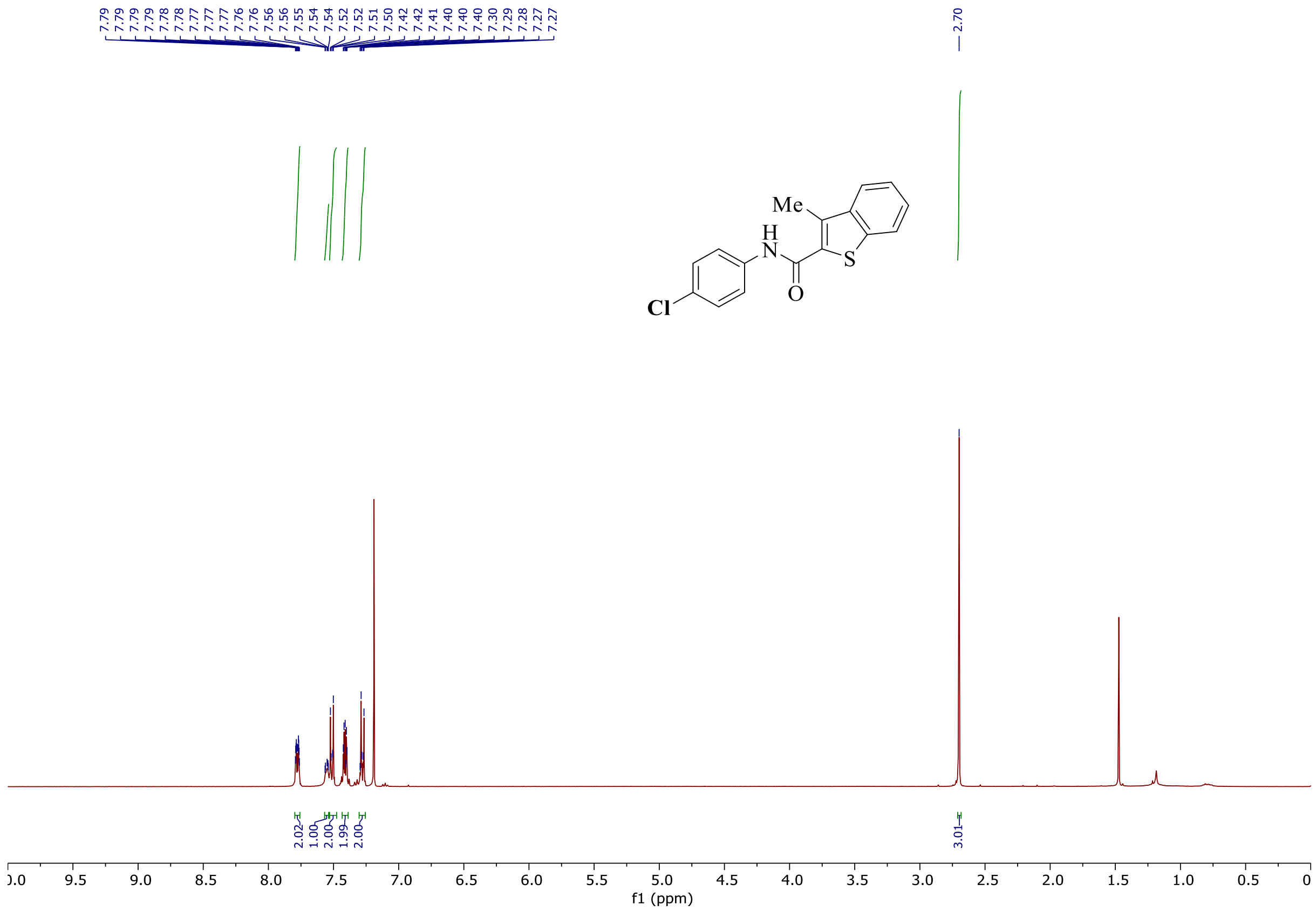

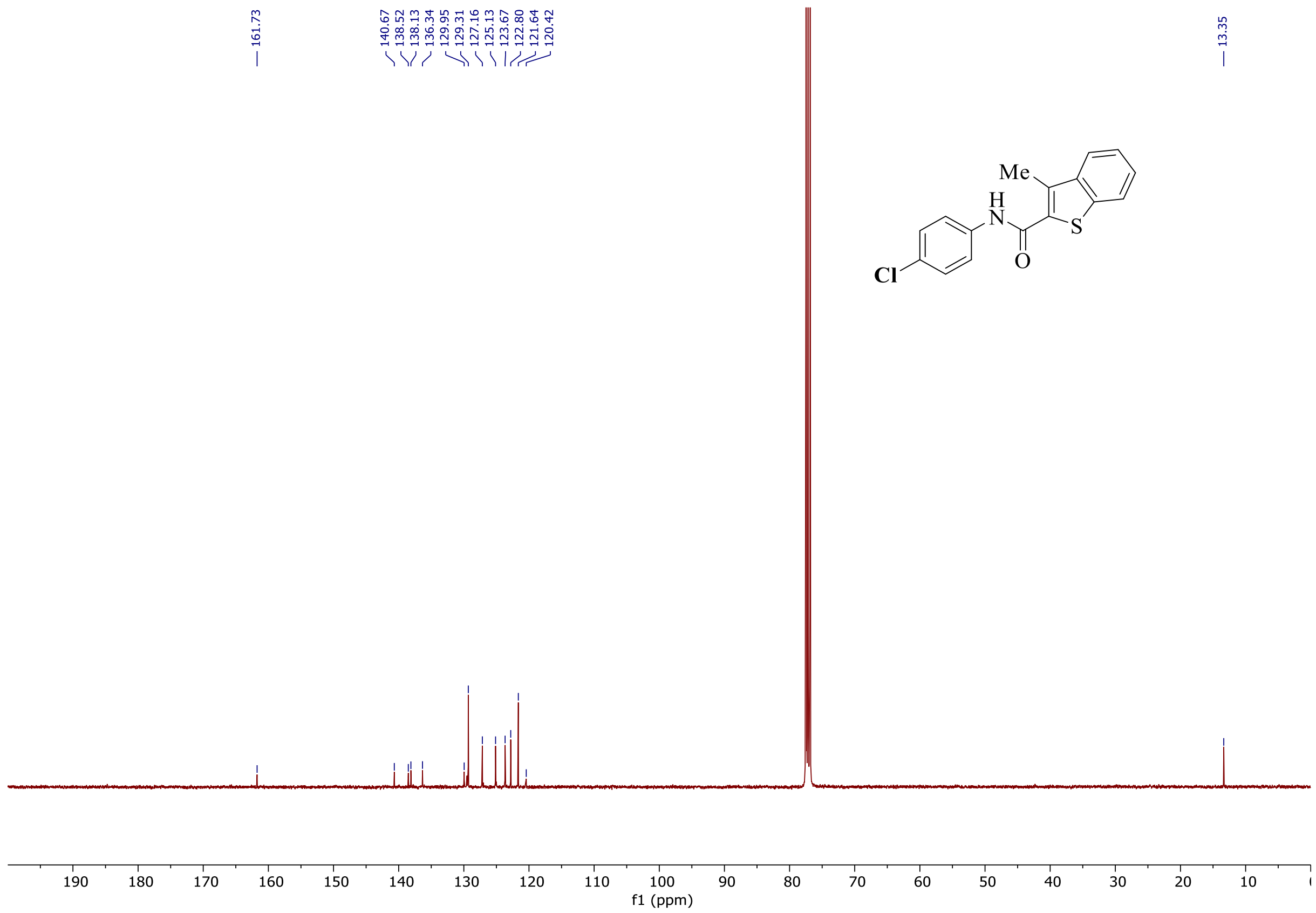

_N_-(4-chlorophenyl)-2-methyl-2-phenylpropanamide: (13)

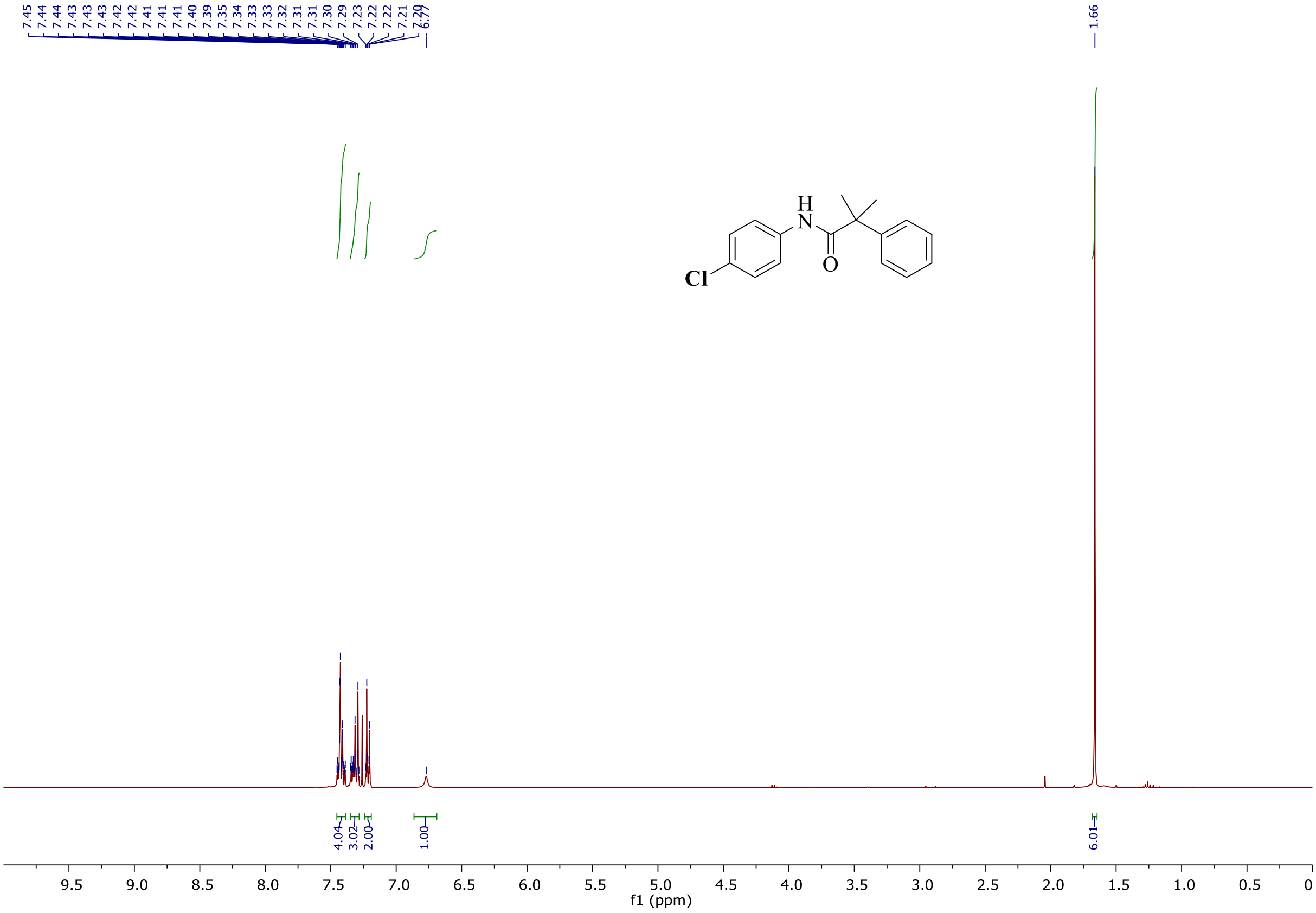

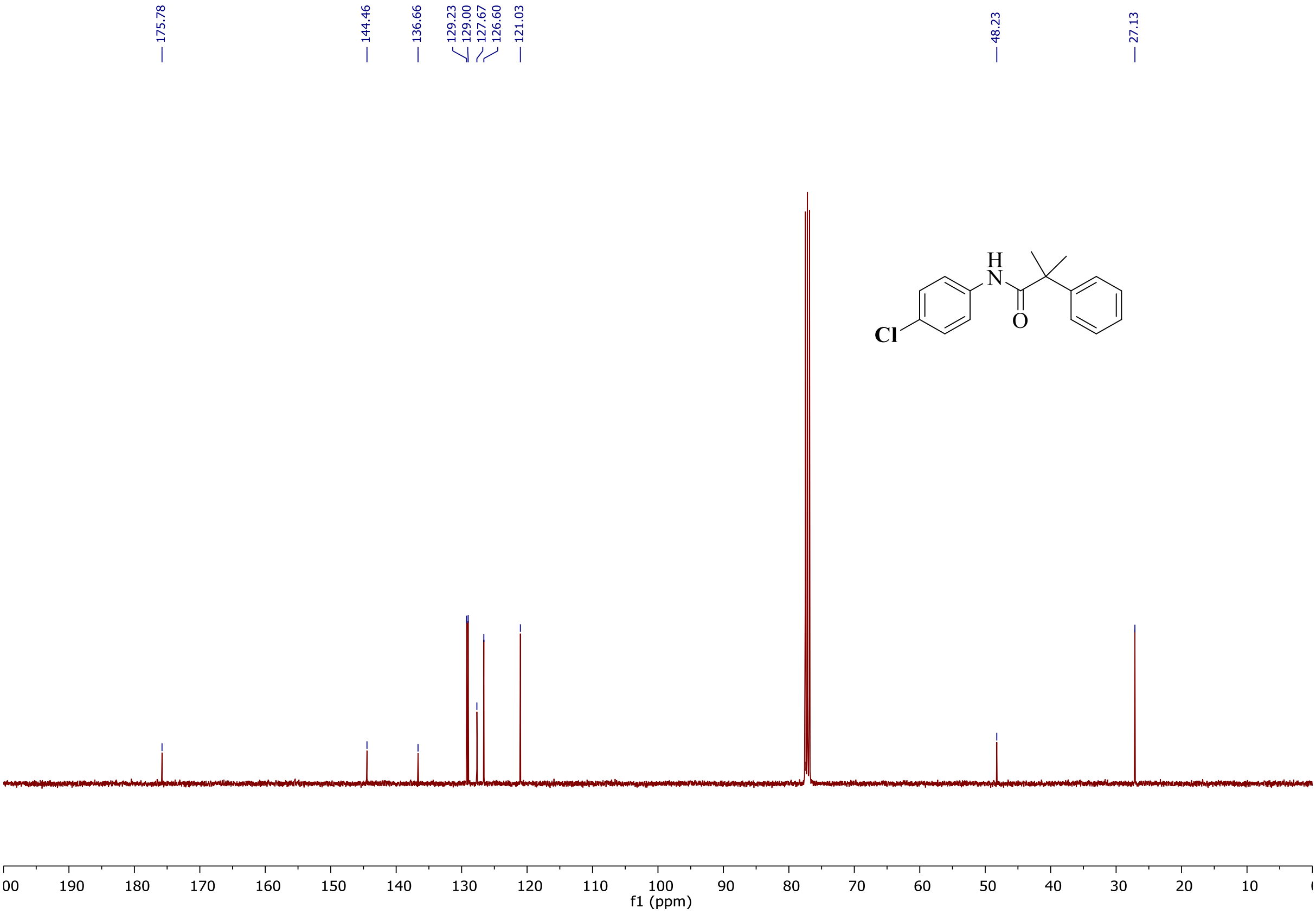

N-(1-chloronaphthalen-2-yl)-2-oxo-2-phenylacetamide: (14)

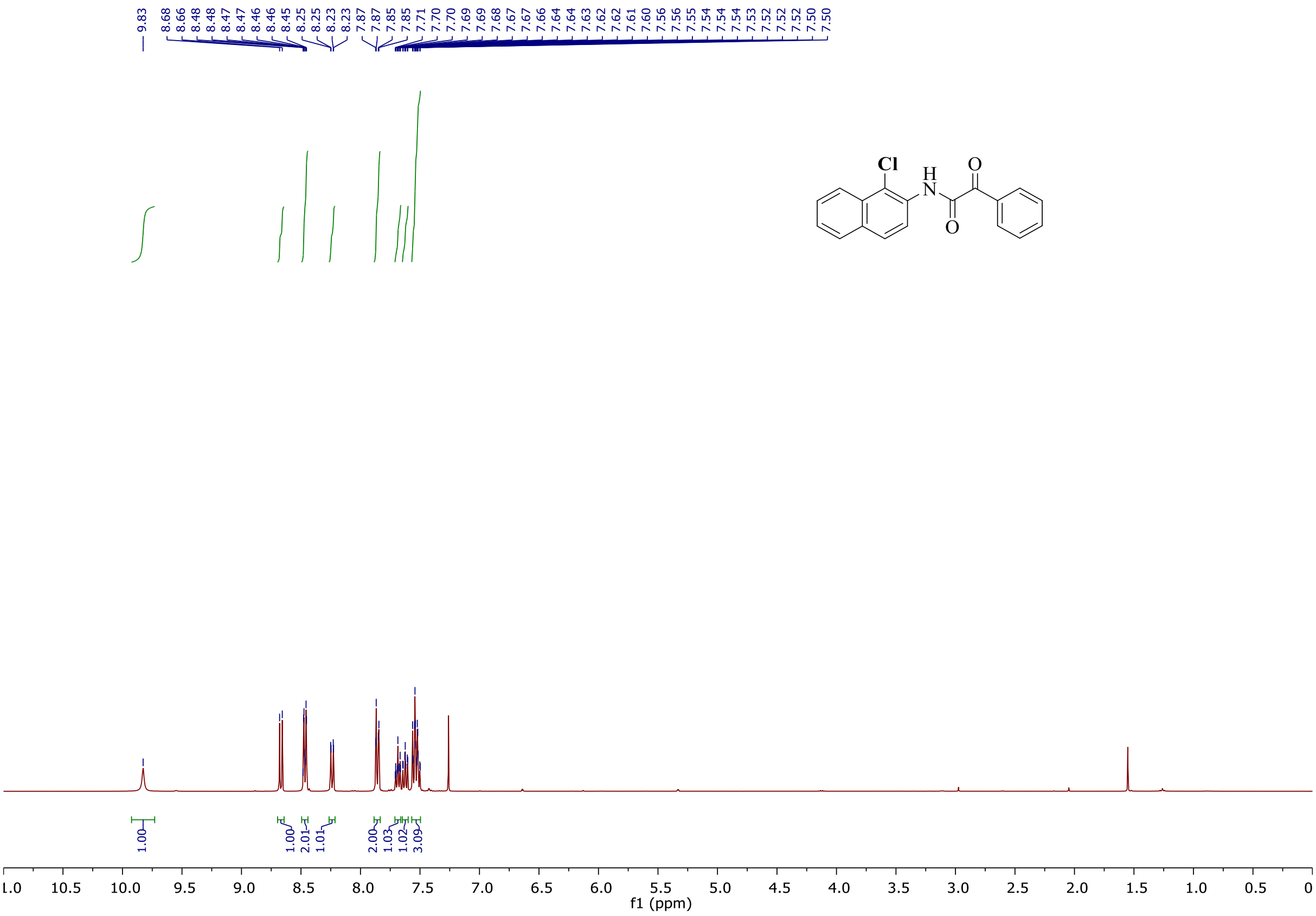

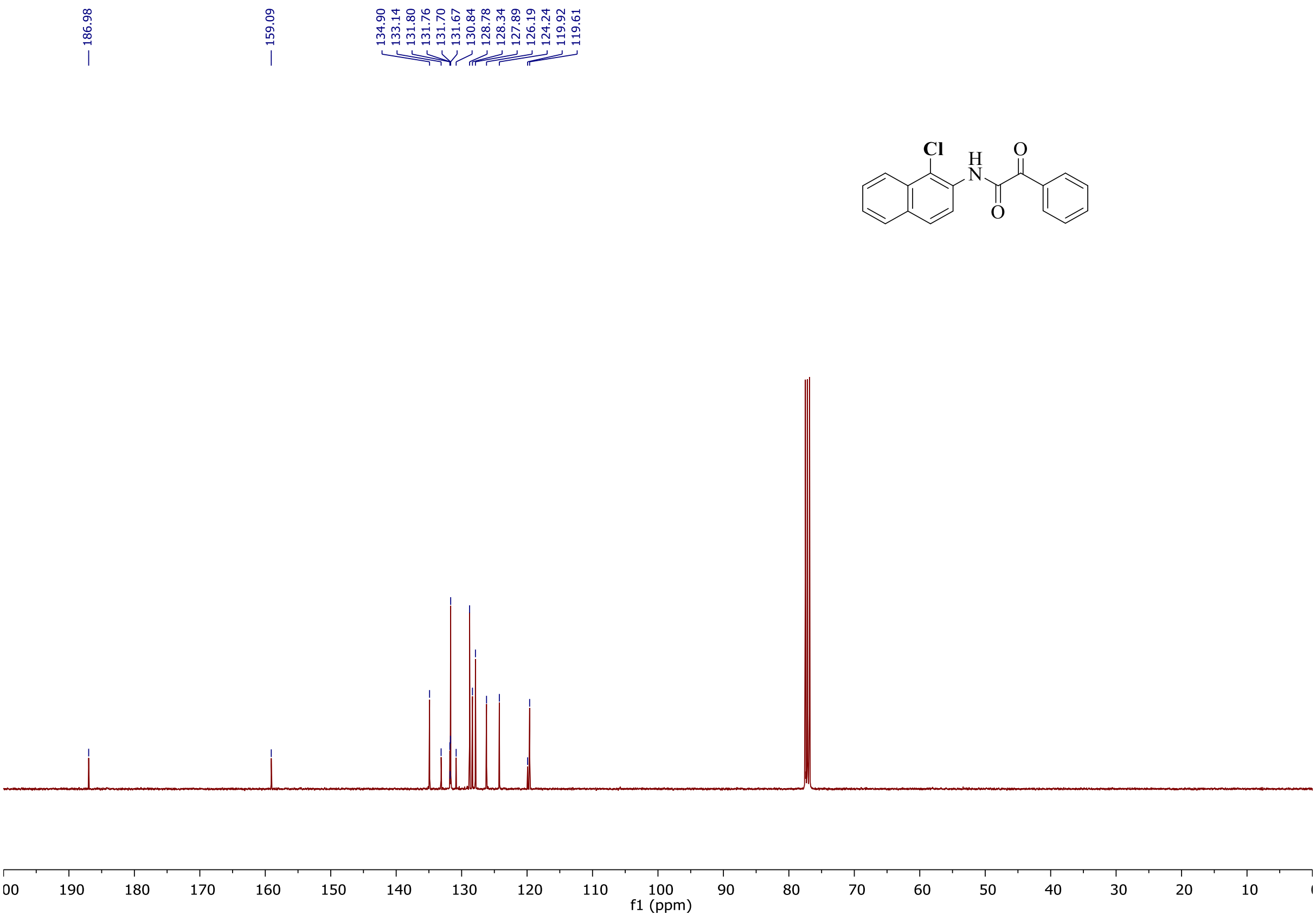

_N_-(4-bromophenyl)cyclohexanecarboxamide: (15)

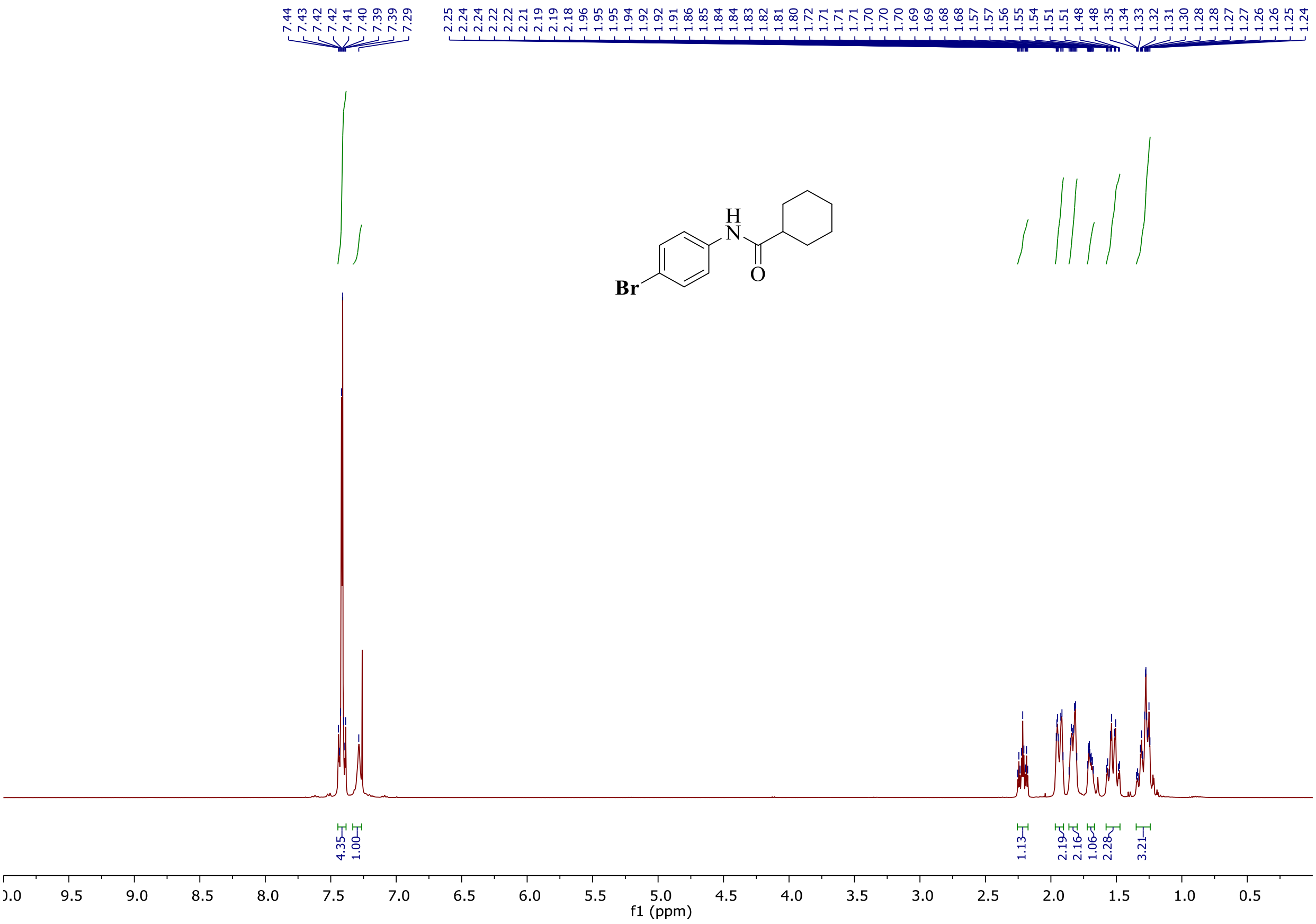

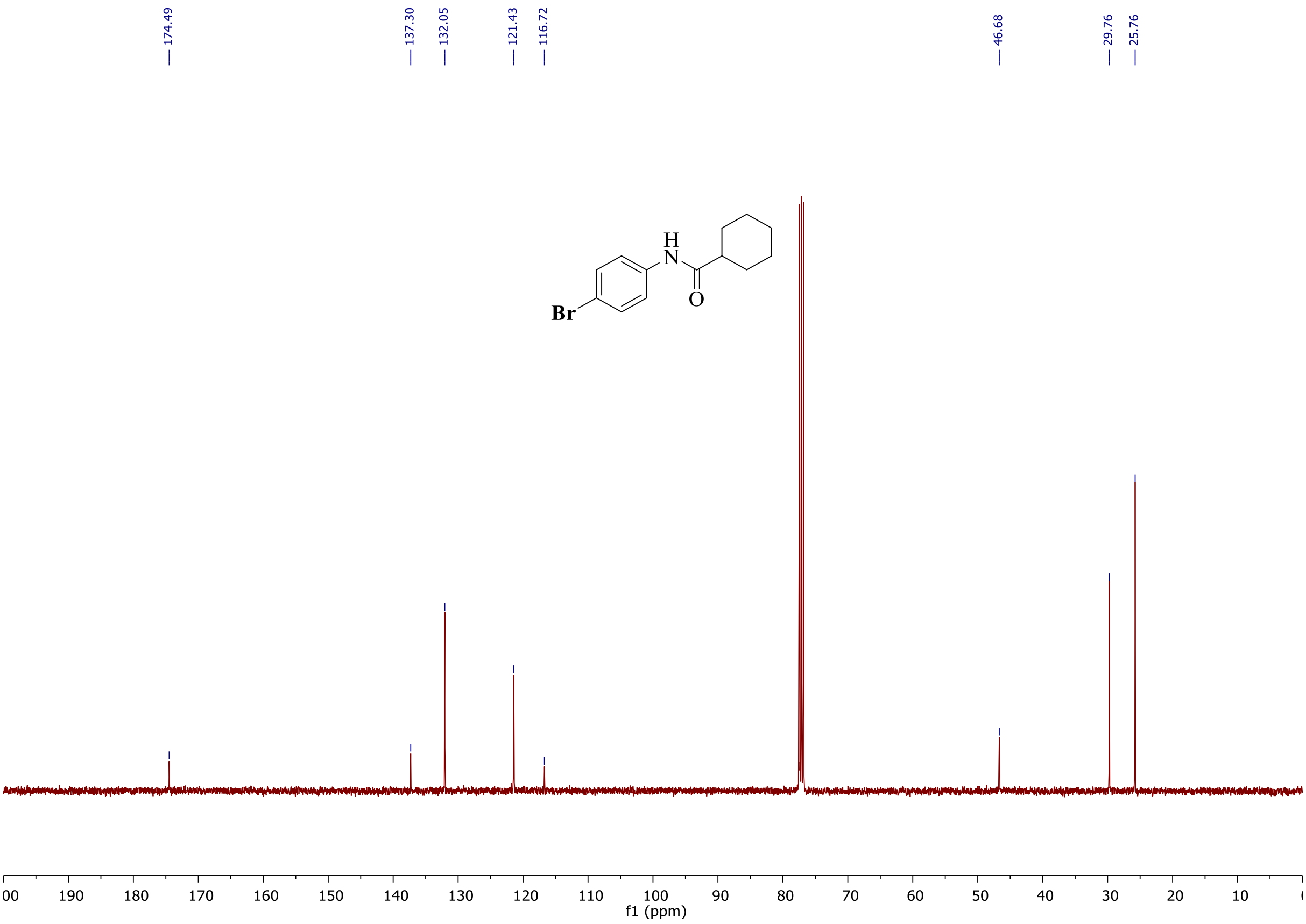

N-(4-bromo-3-methylphenyl)cyclohexanecarboxamide: (16)

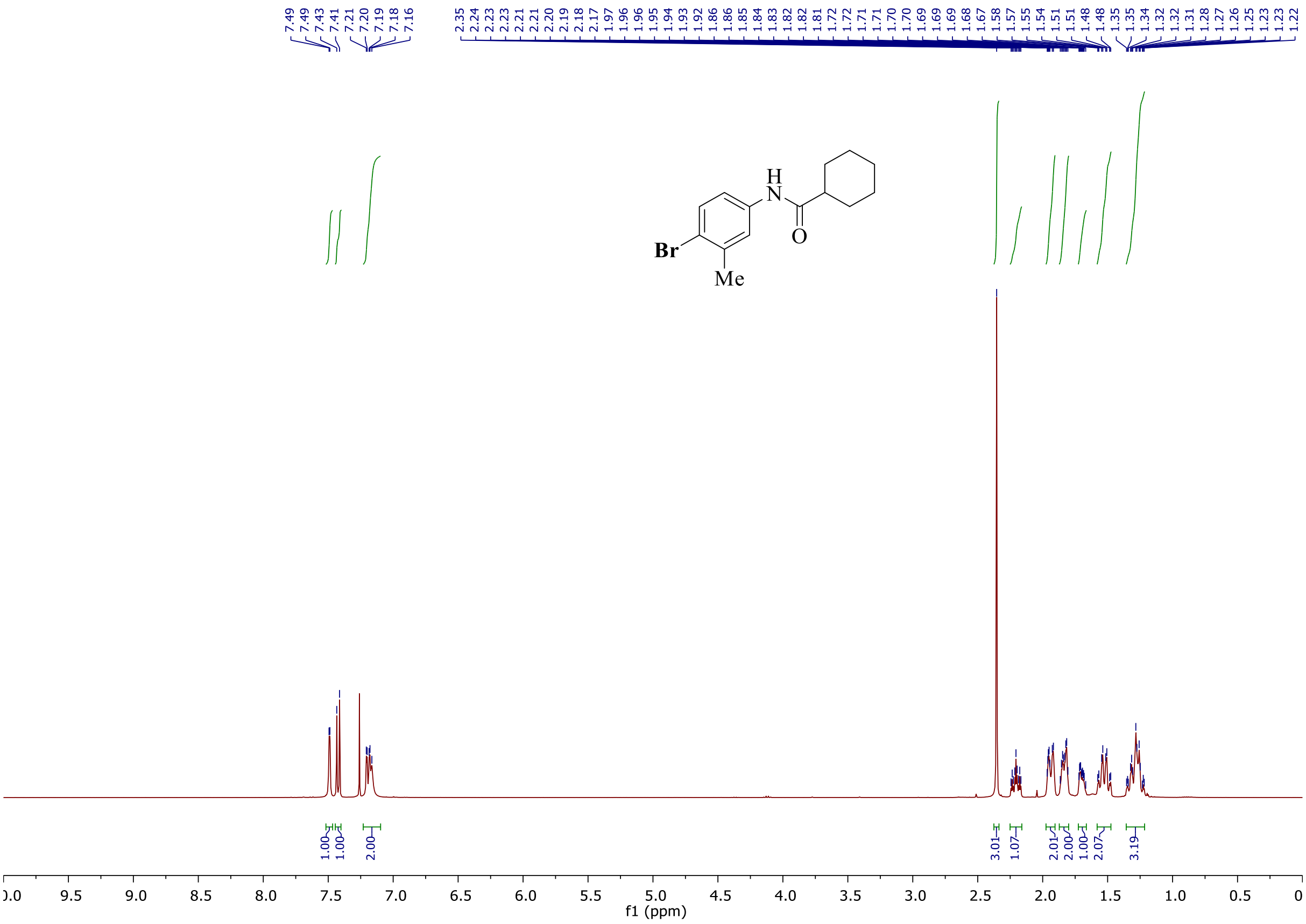

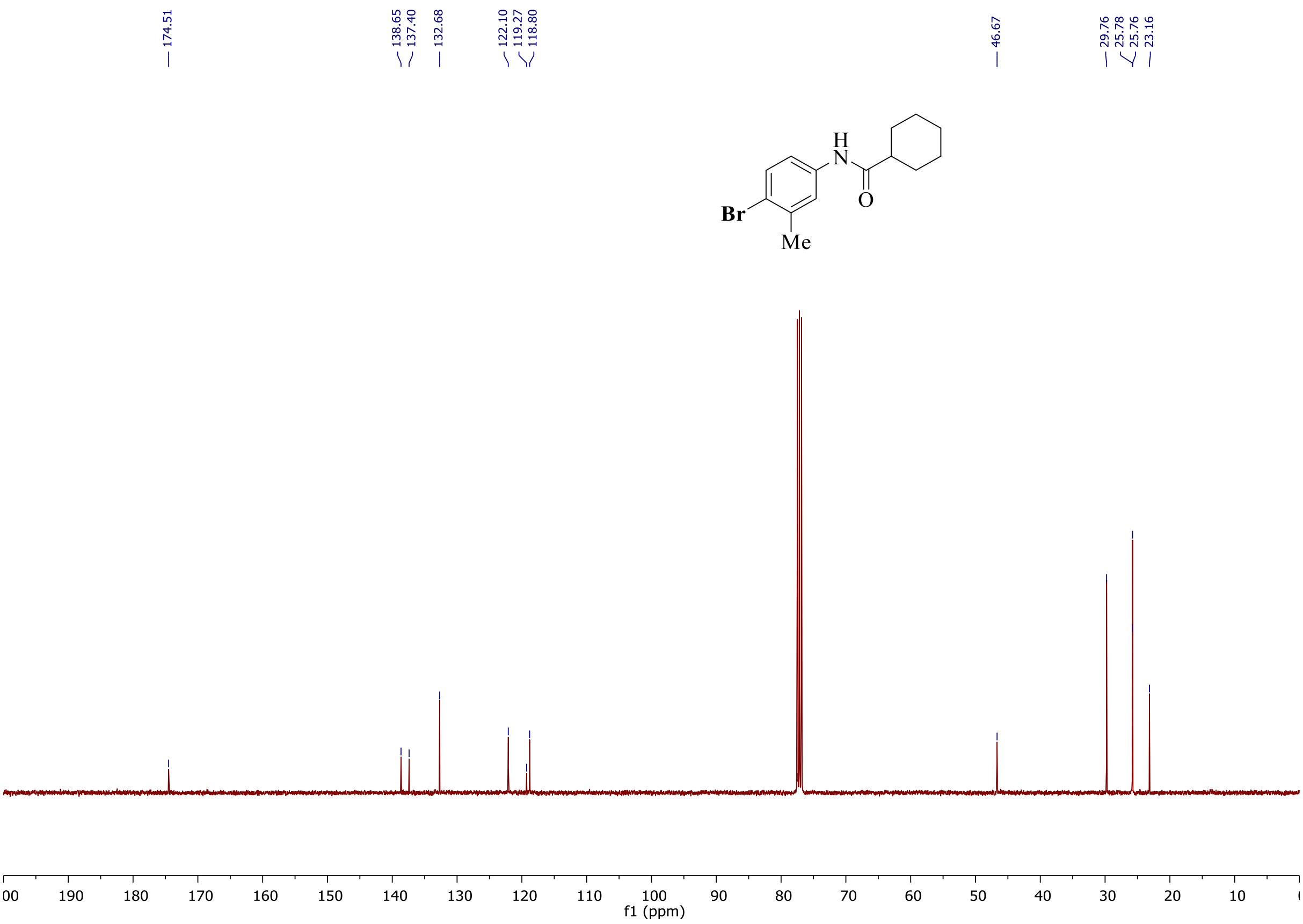

_N_-(1-bromonaphthalen-2-yl)cyclohexanecarboxamide: (17)

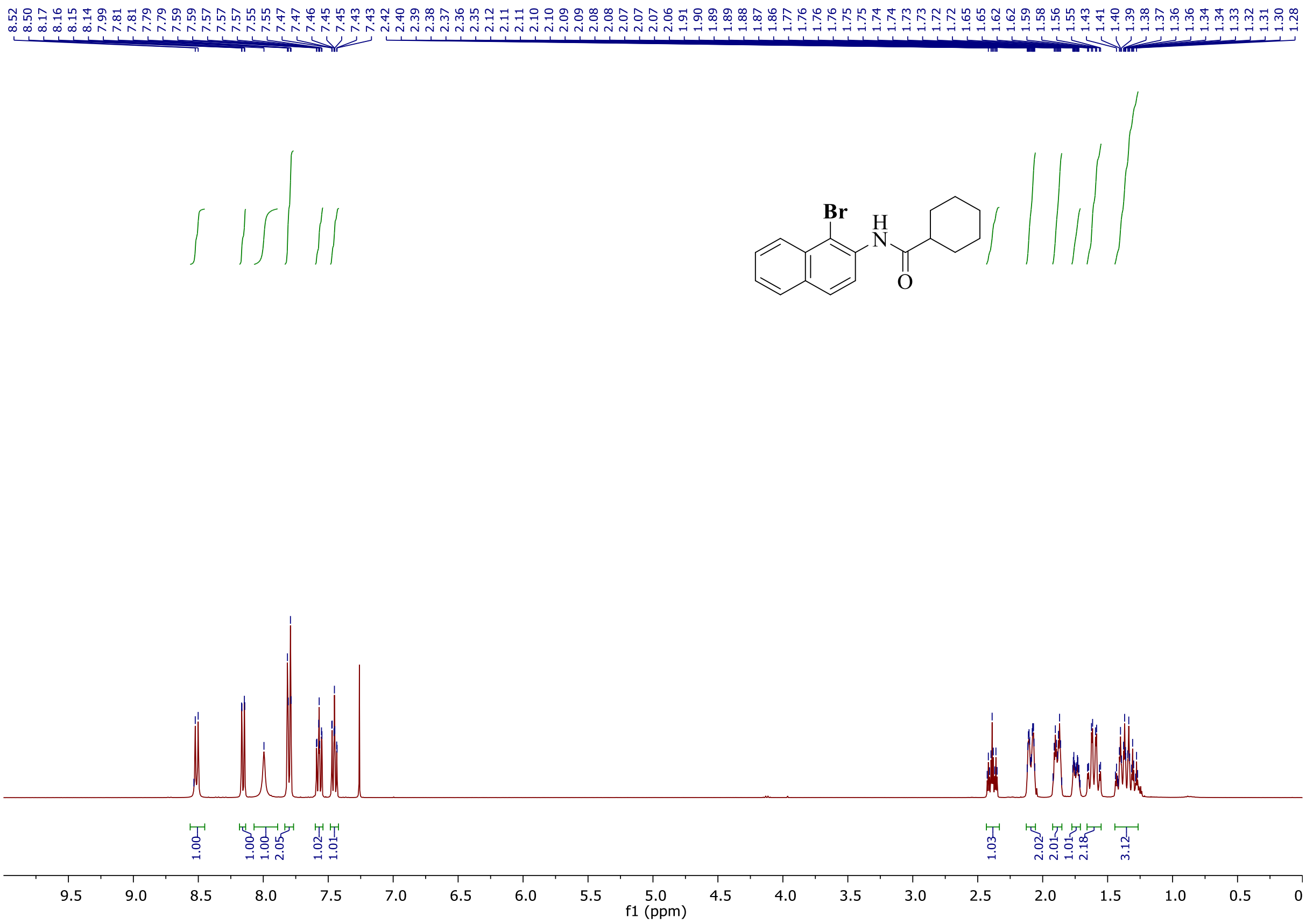

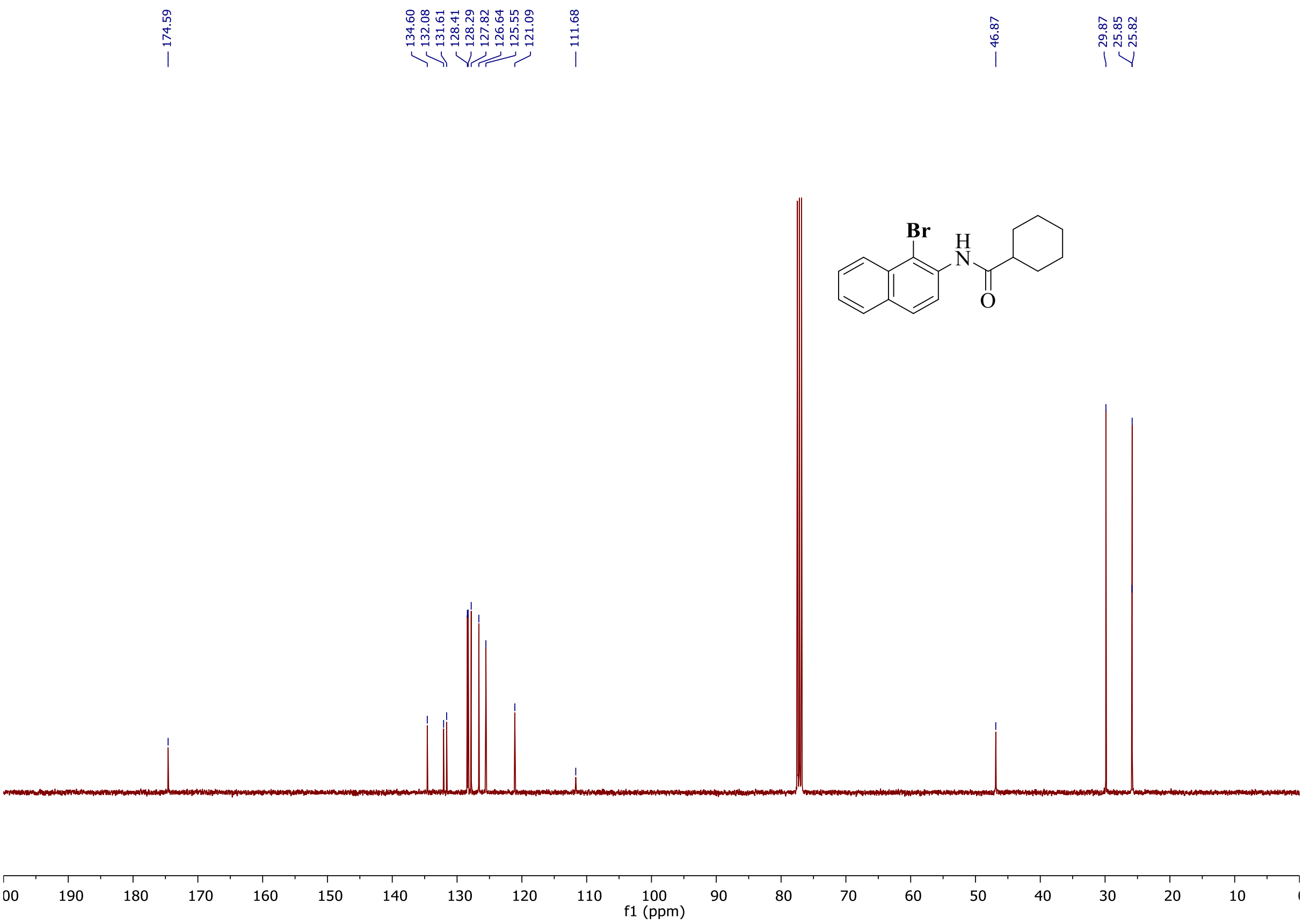

2-(4-(4-chlorobenzoyl)phenoxy)-*N*-(4-chlorophenyl)-2-methylpropanamide: (18)

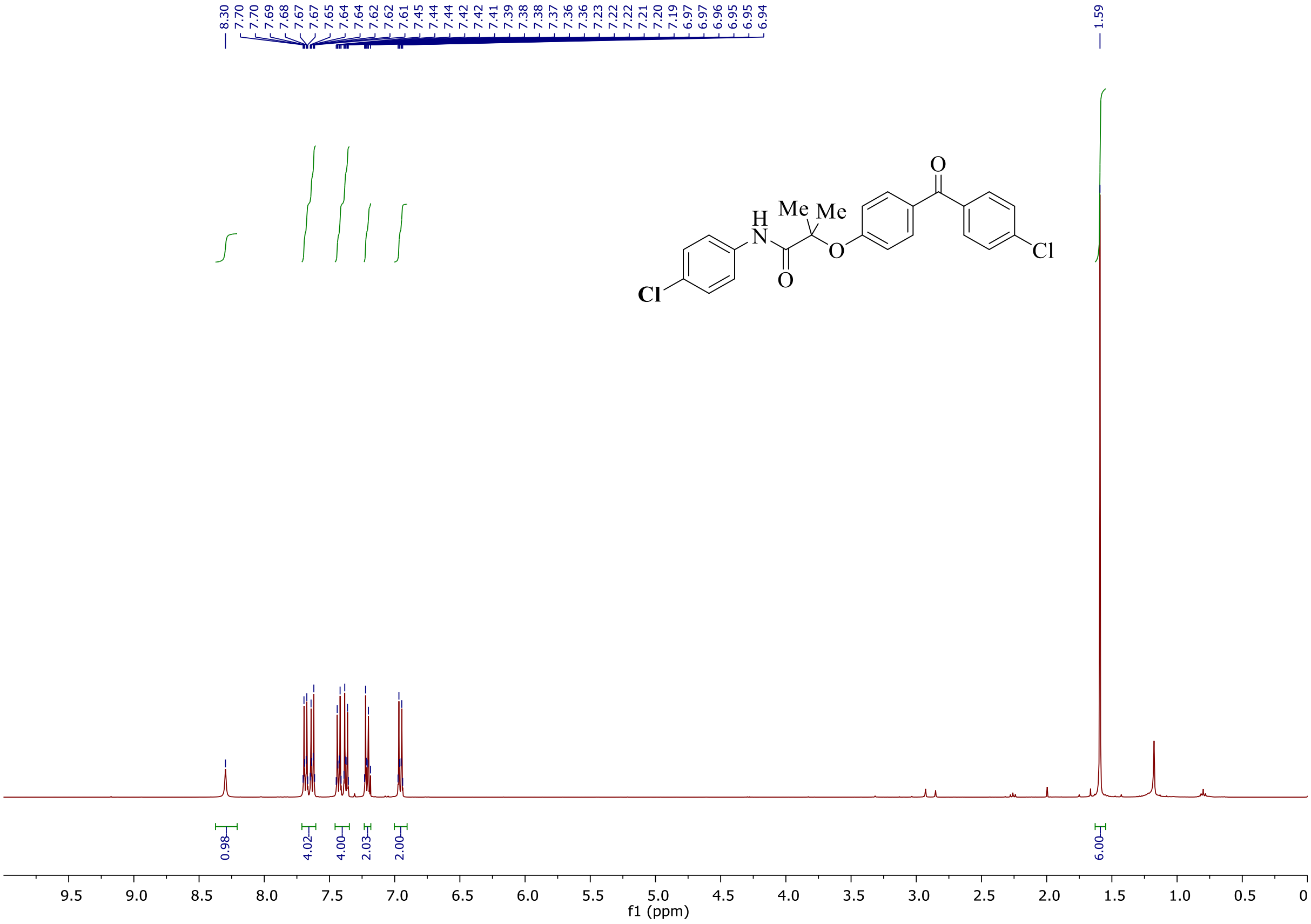

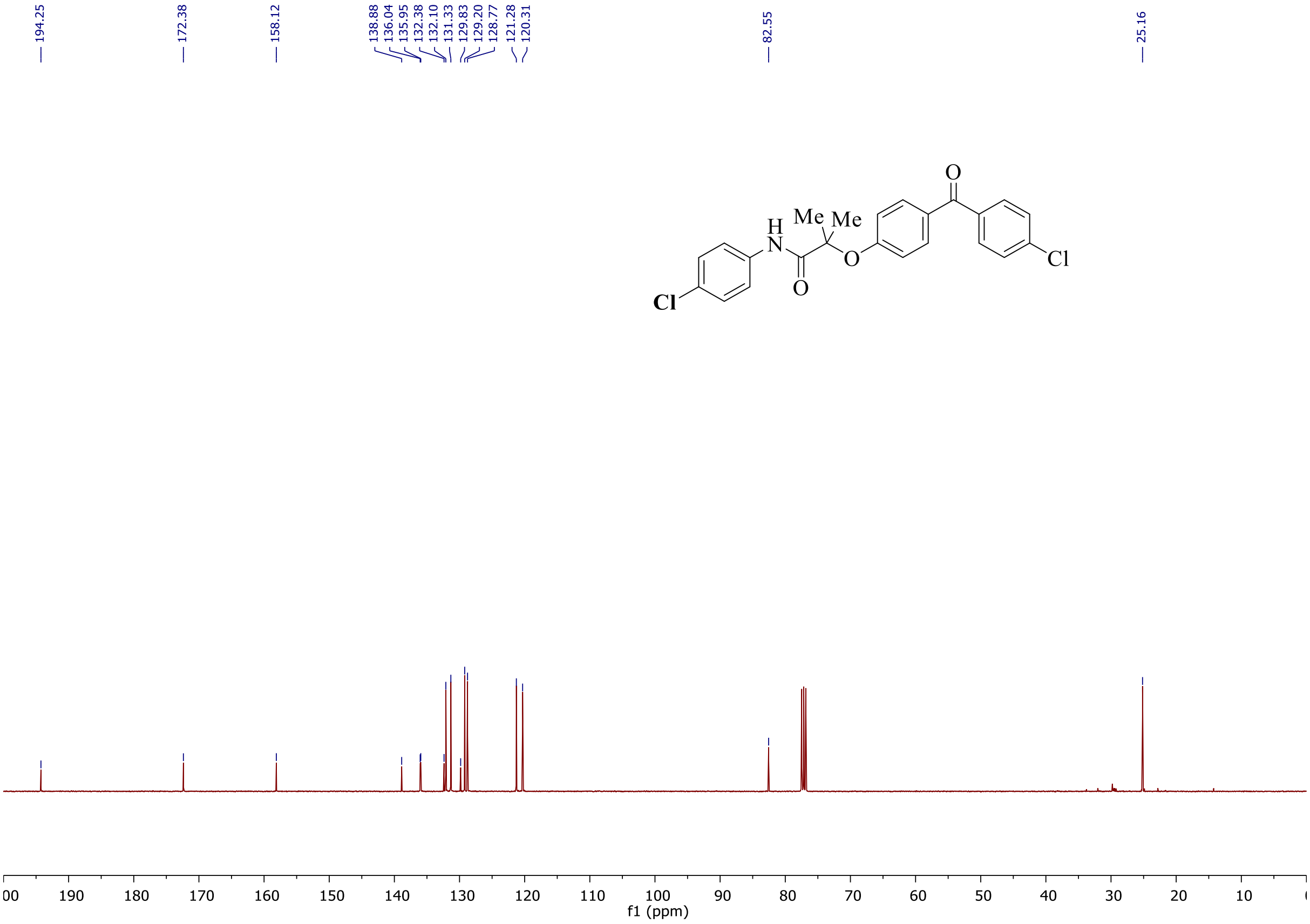

***N*-(4-chlorophenyl)-2-(4-(2,2-dichlorocyclopropyl)phenoxy)-2-methylpropanamide: (19)**

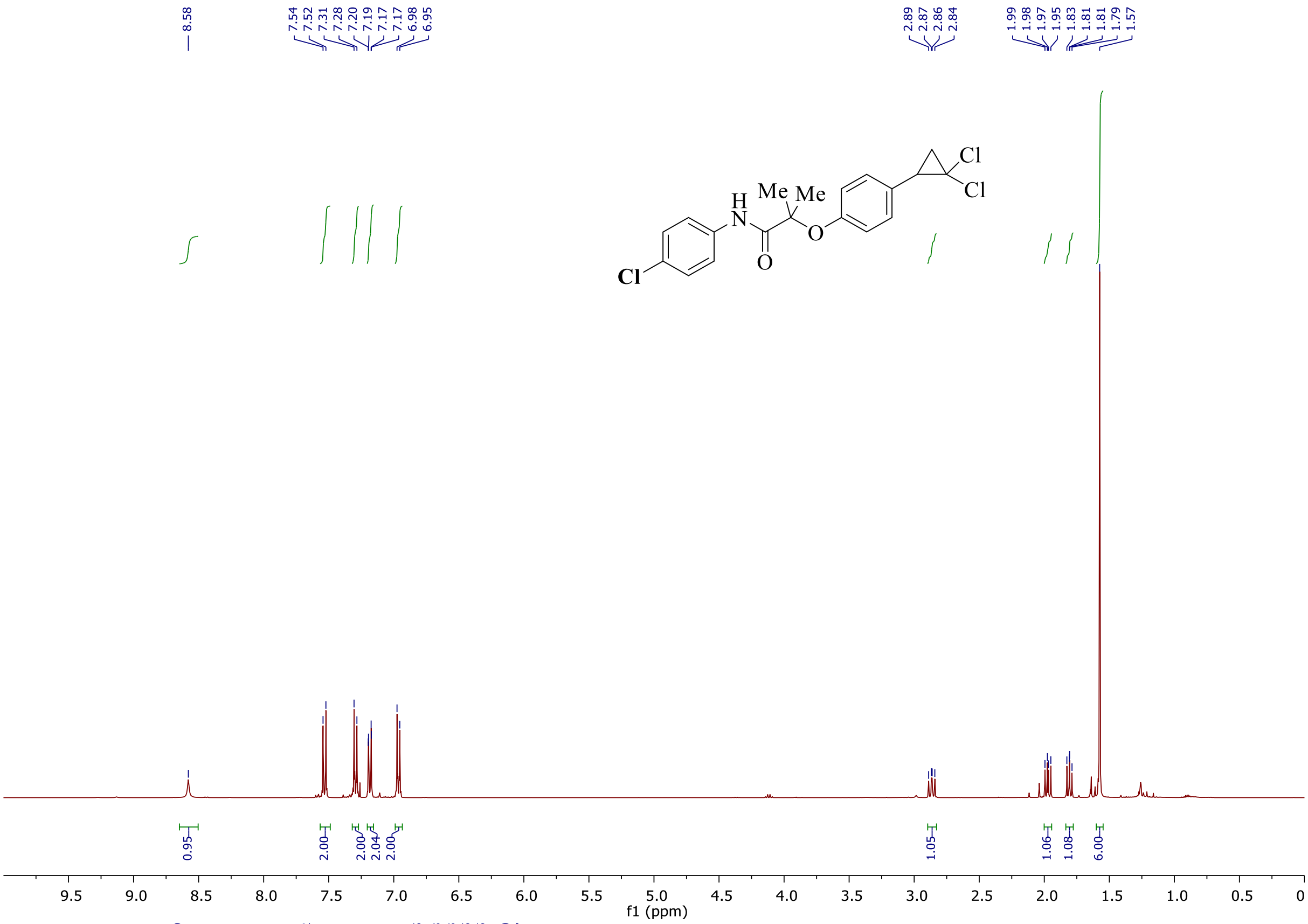

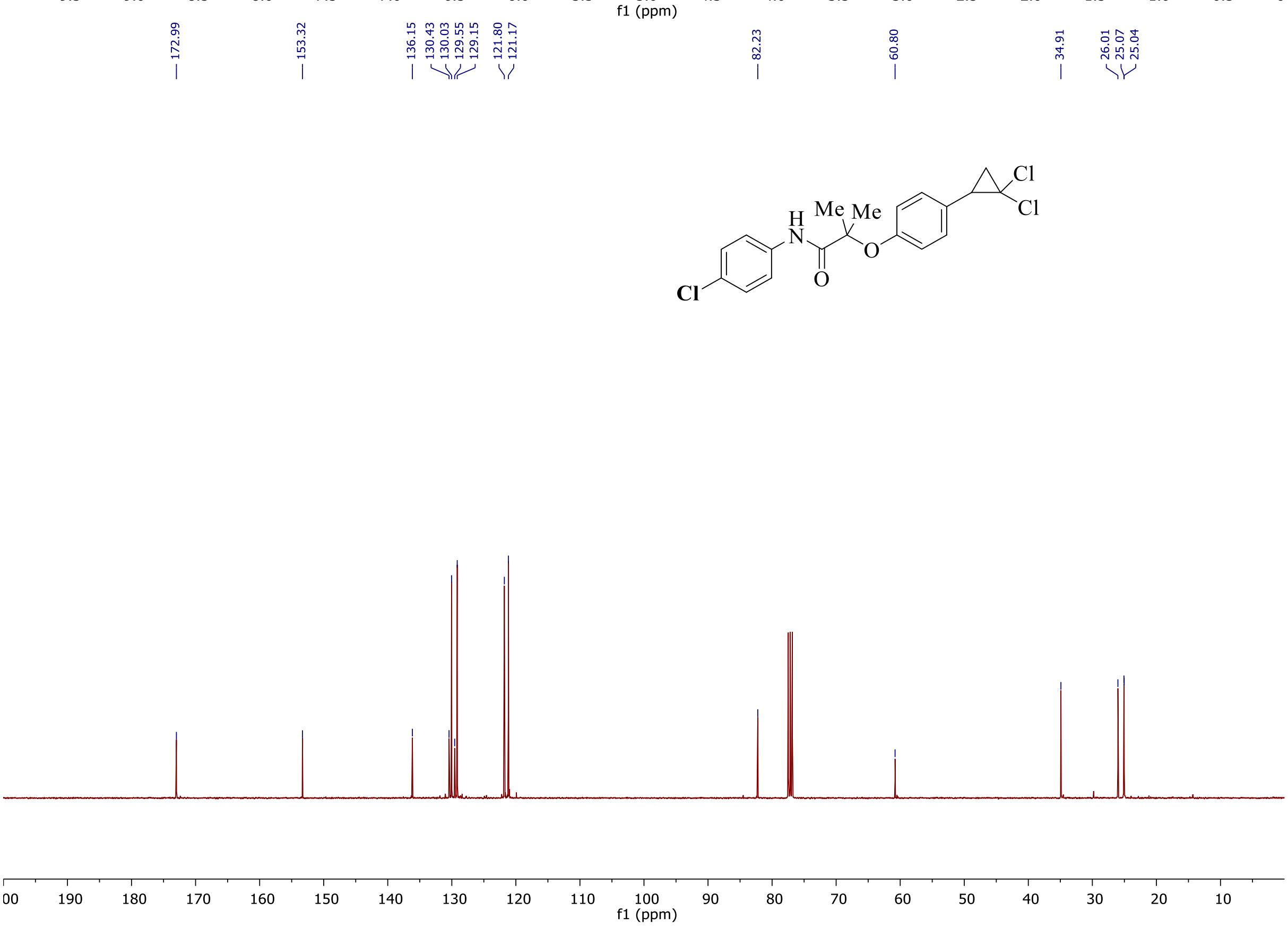

***N*-(4-chlorophenyl)-5-(2,5-dimethylphenoxy)-2,2-dimethylpentanamide: (20)**

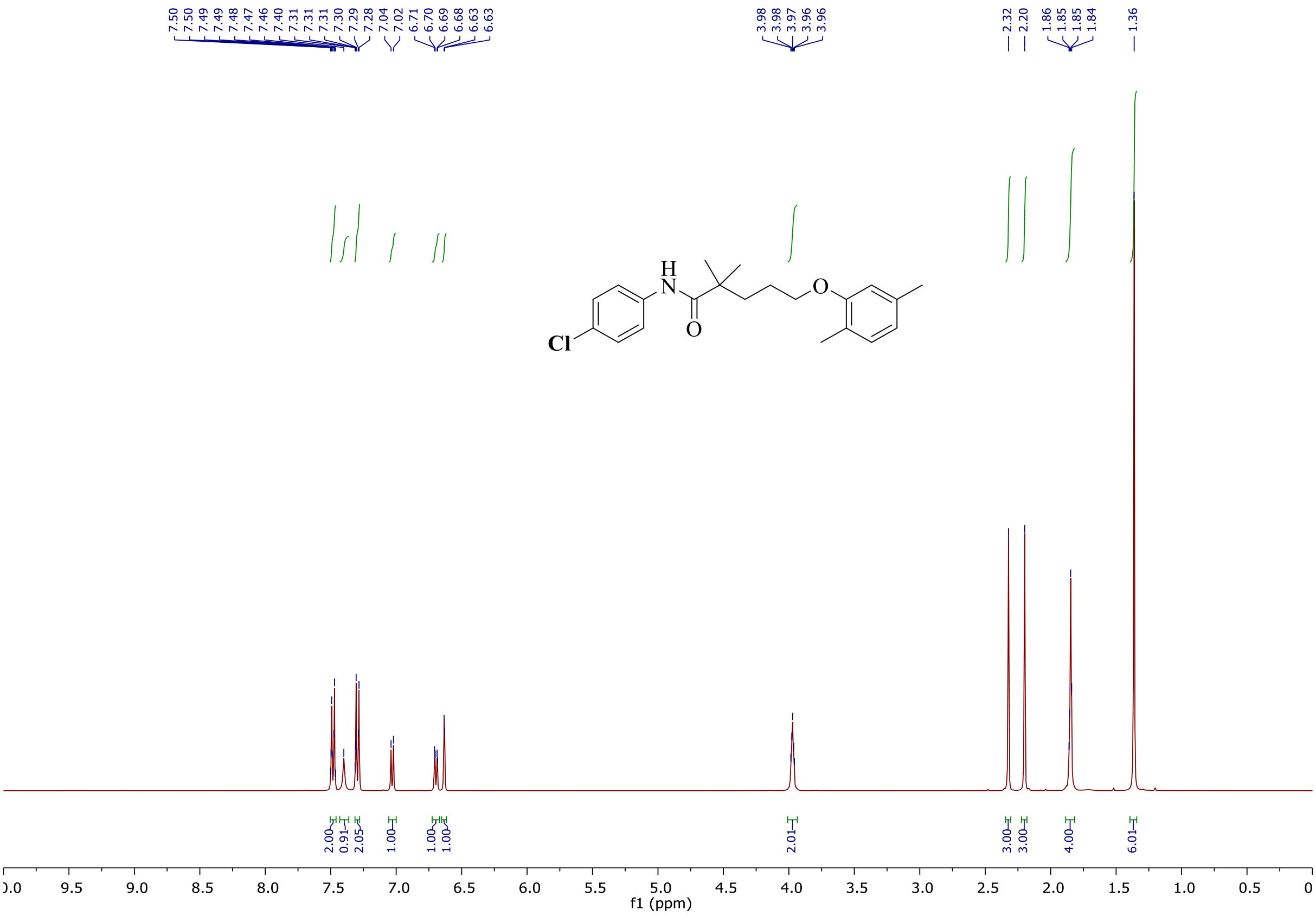

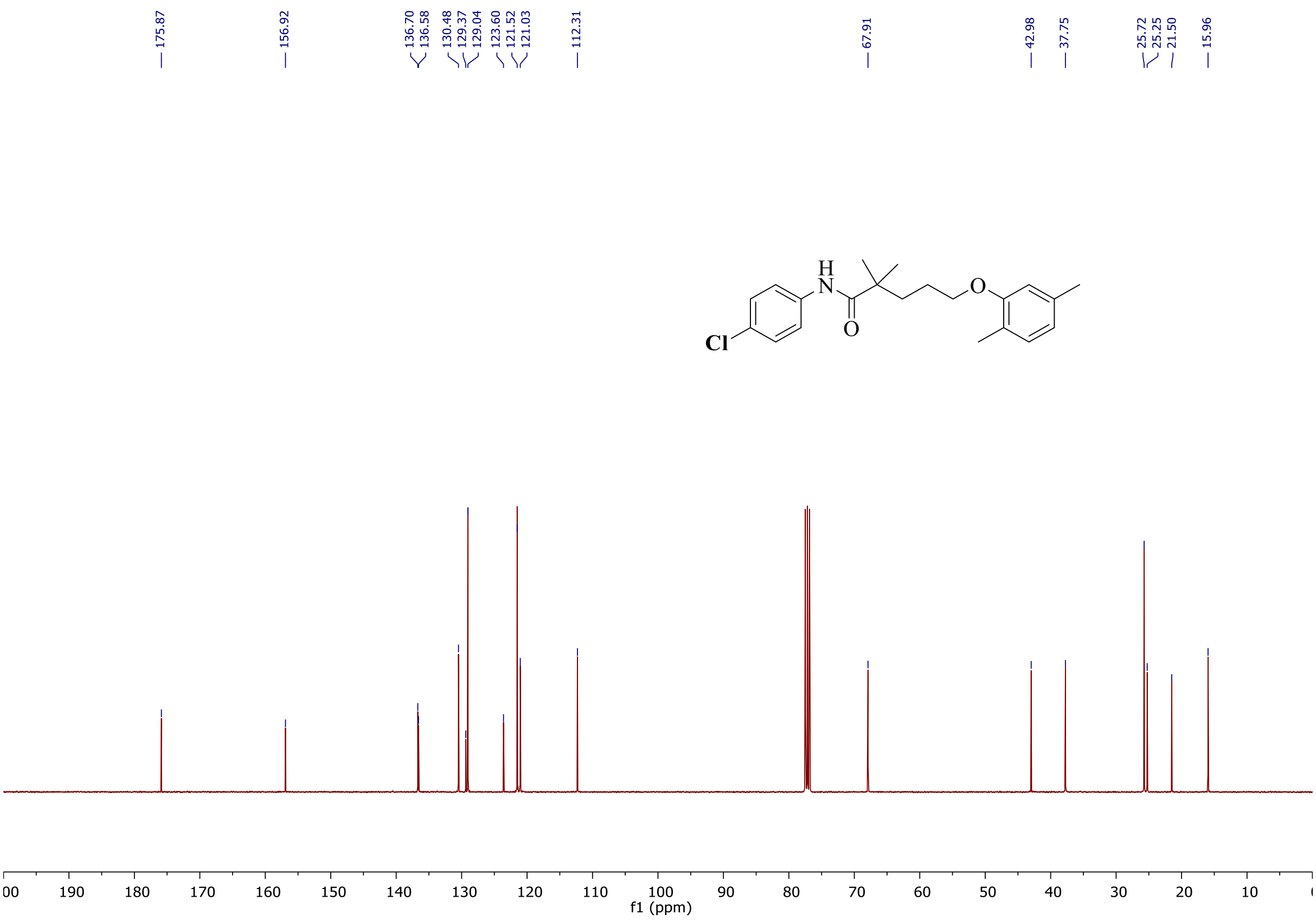

(*R*)-*N*-(4-chlorophenyl)-2-(7-methoxynaphthalen-2-yl)propanamide: (21)

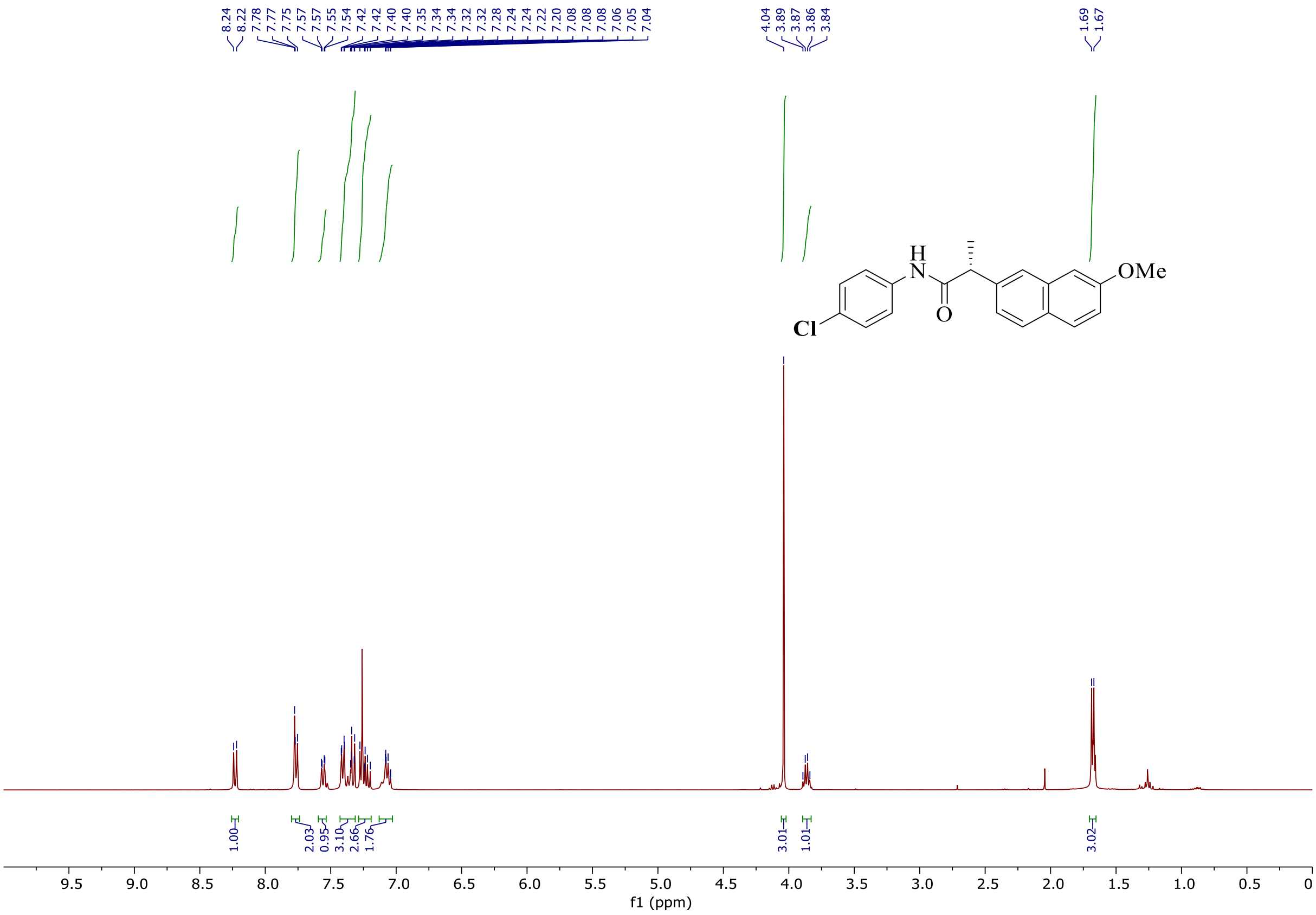

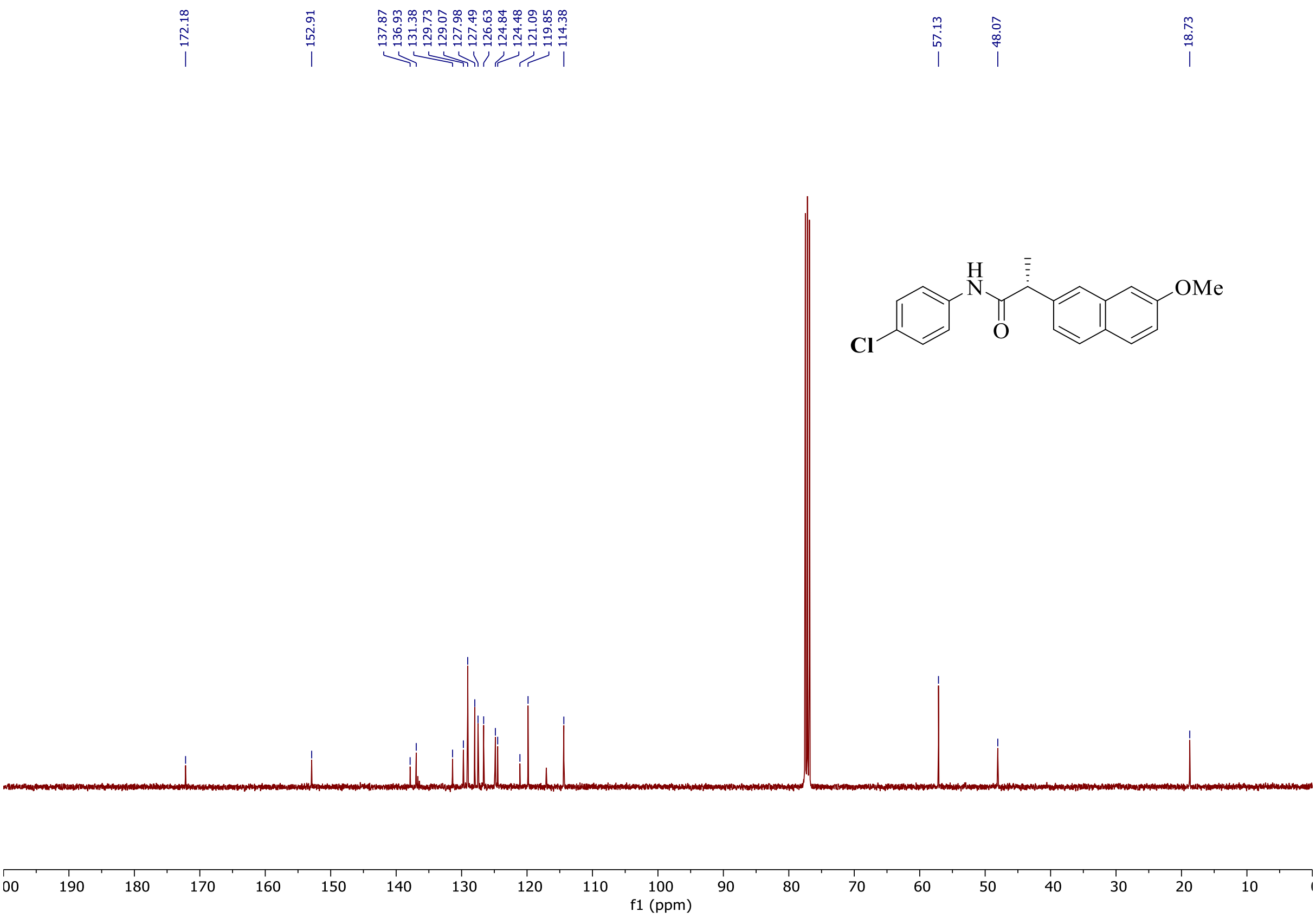

2-(3-benzoylphenyl)-*N*-(4-chlorophenyl)propanamide: (22)

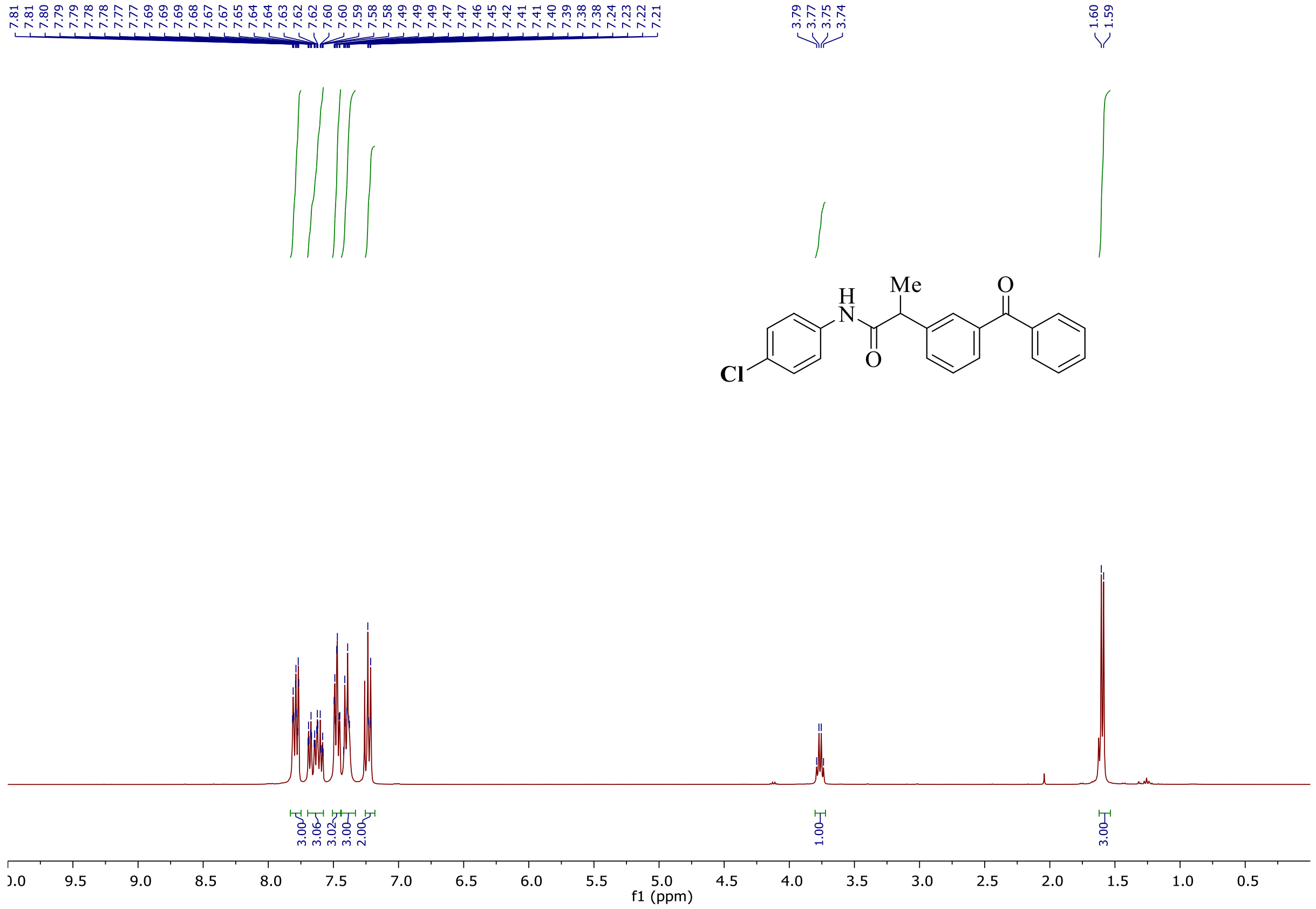

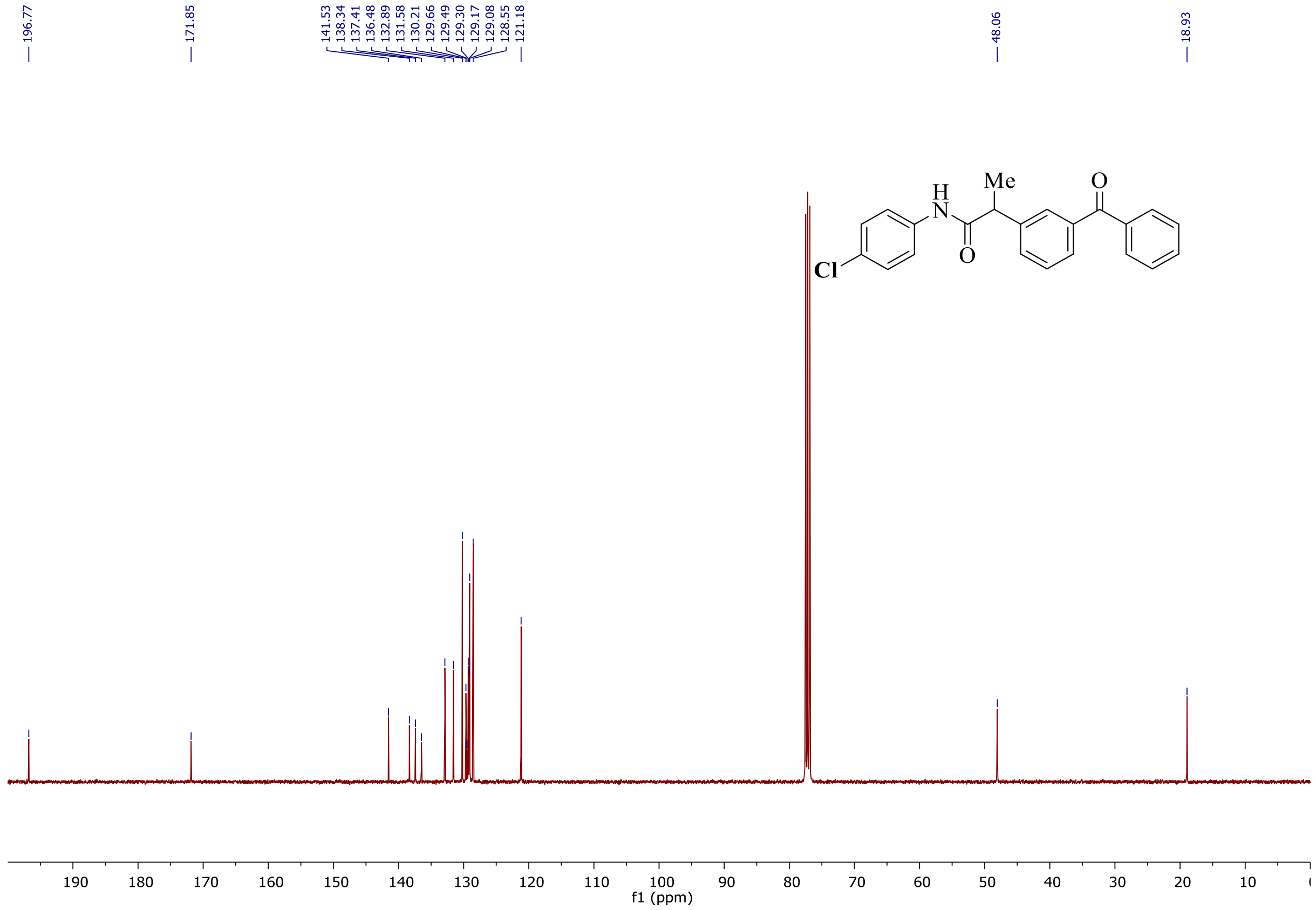

_N_-(4-chlorophenyl)-2-(4-isobutylphenyl)propanamide: (23)

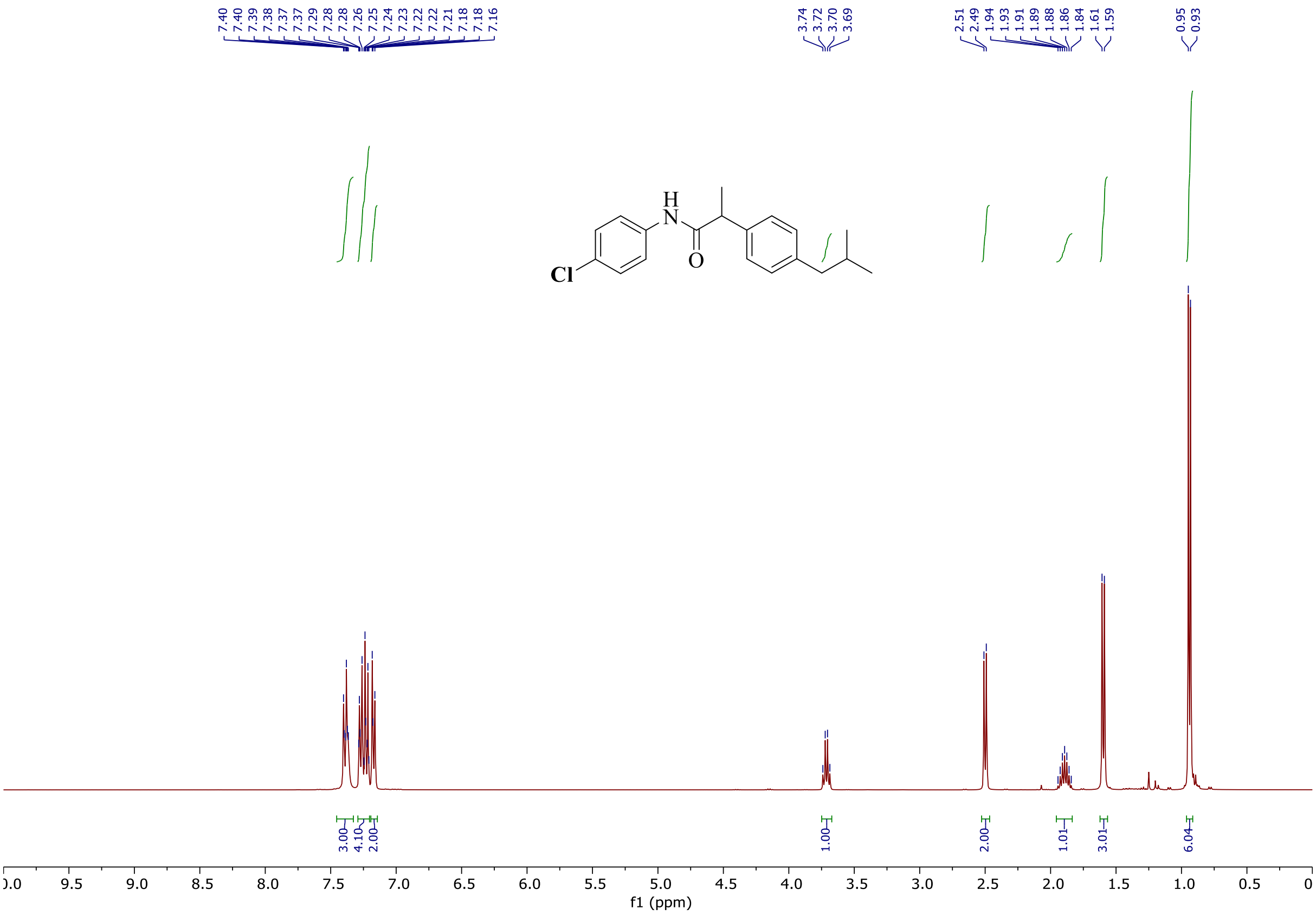

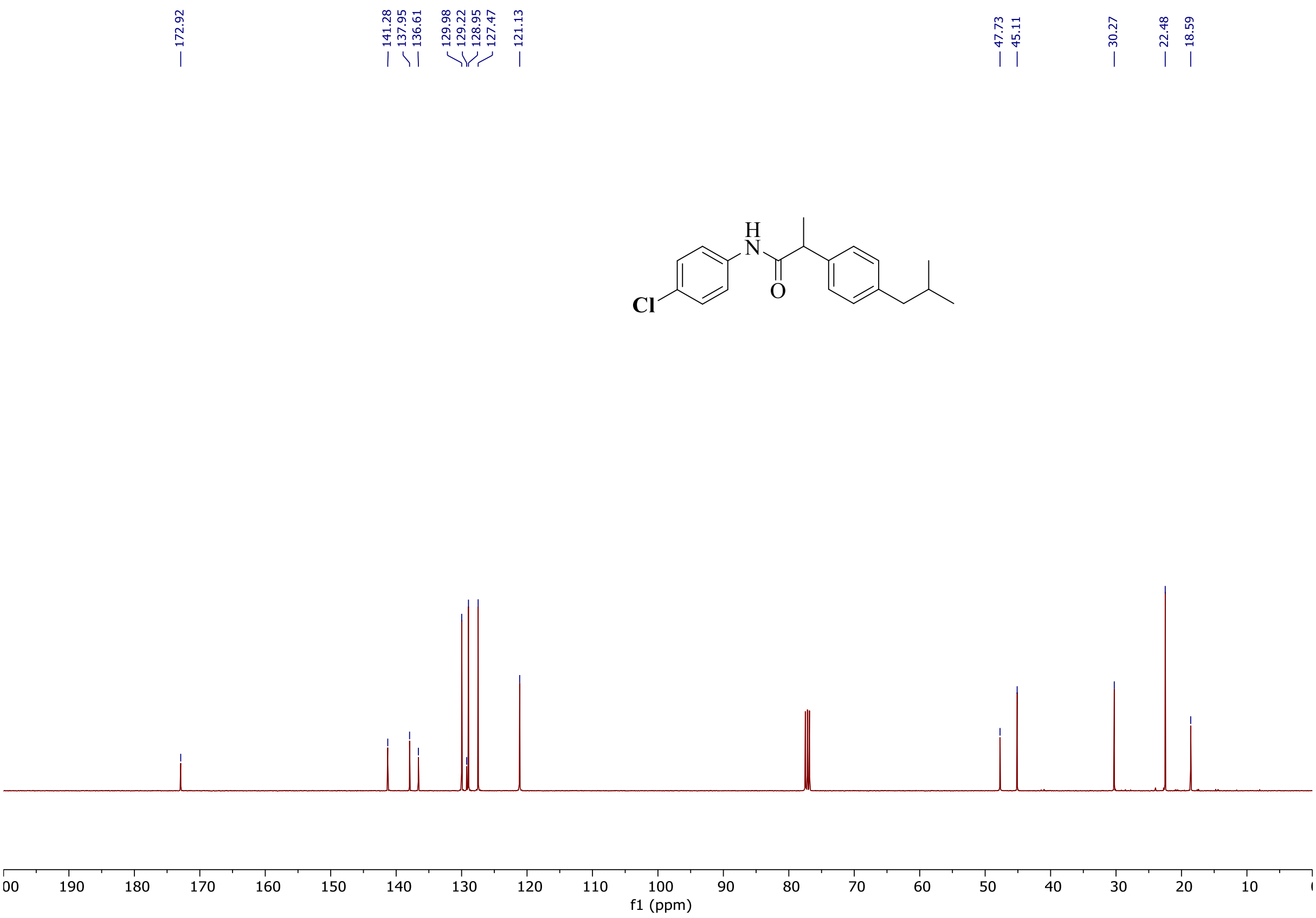

N-(1-chloronaphthalen-2-yl)-2-(2-fluoro-[1,1'-biphenyl]-4-yl)propanamide: (24)

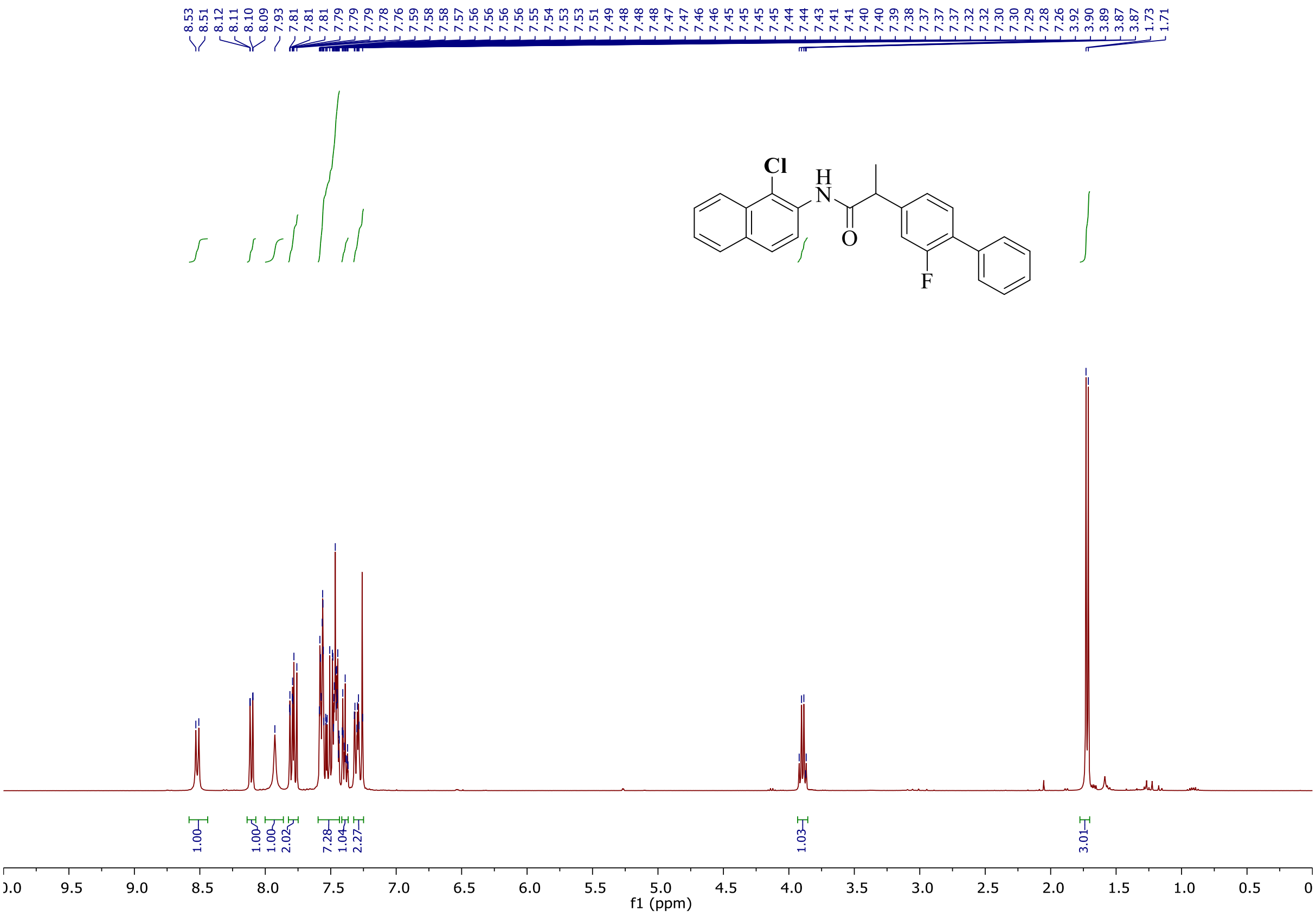

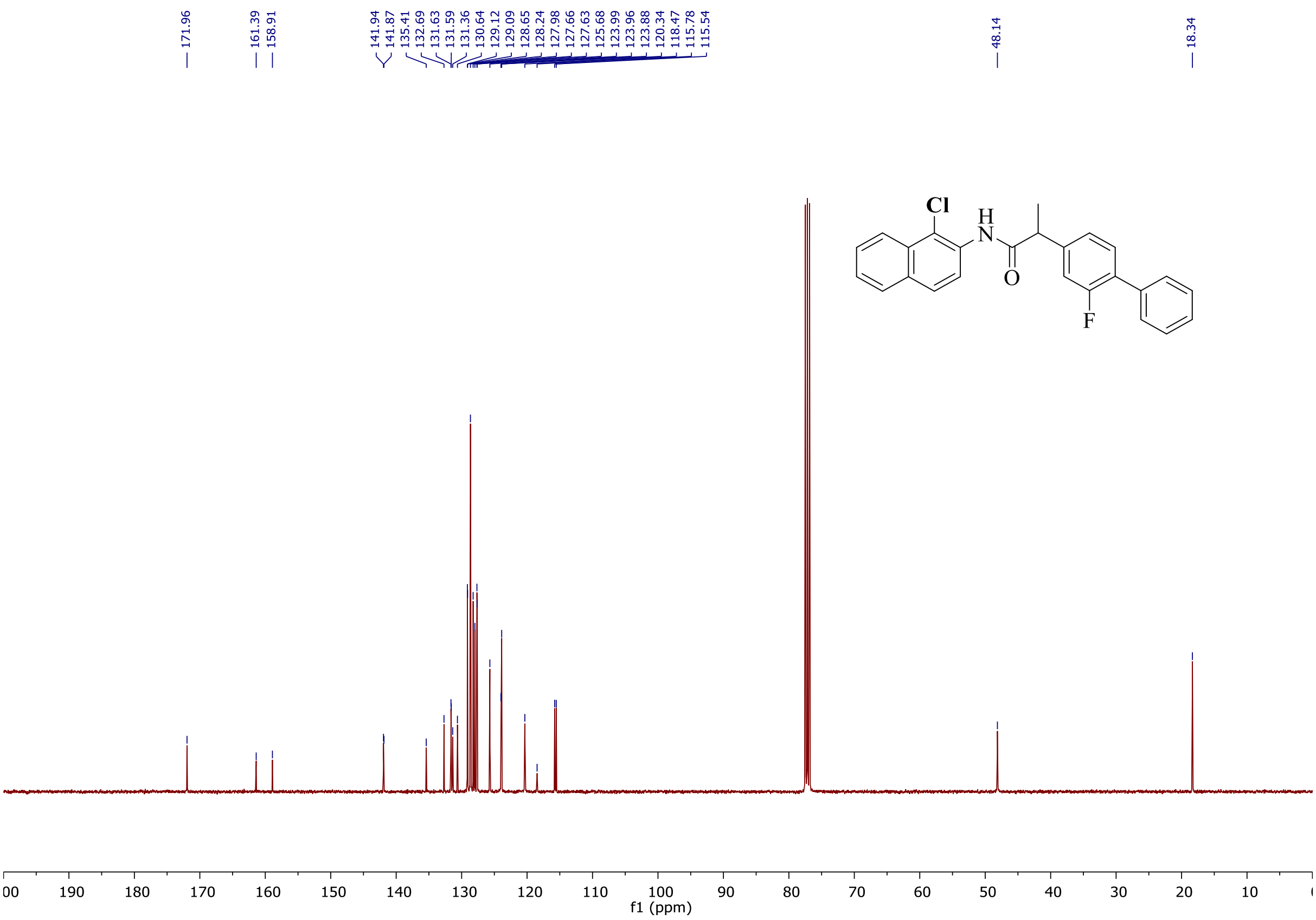

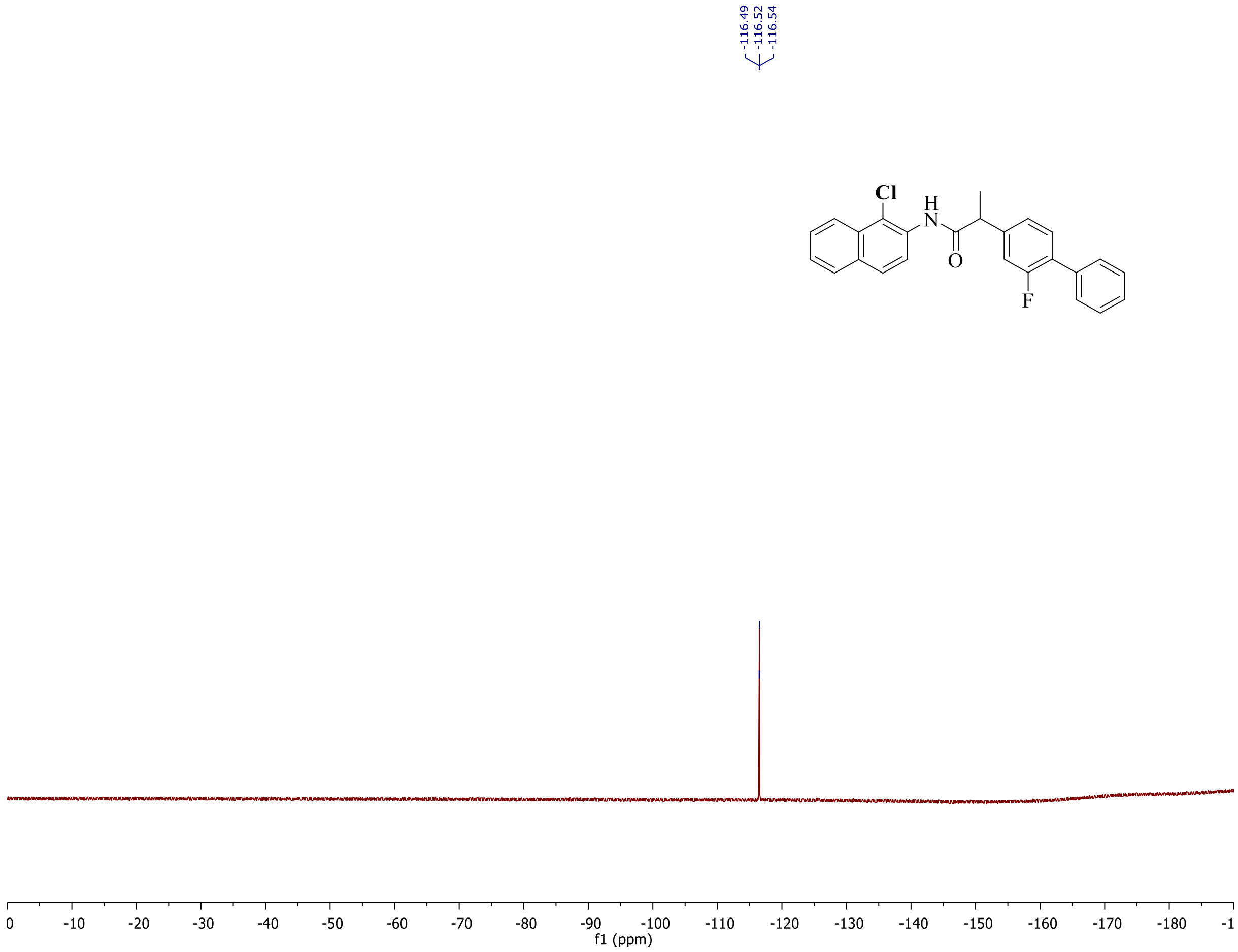
-116.49
-116.52
-116.54
Cl
H
N
O
F
0 -10 -20 -30 -40 -50 -60 -70 -80 -90 -100 -110 -120 -130 -140 -150 -160 -170 -180 -1
f1 (ppm)

2-((4-chlorophenyl)carbamoyl)phenyl acetate: (25)

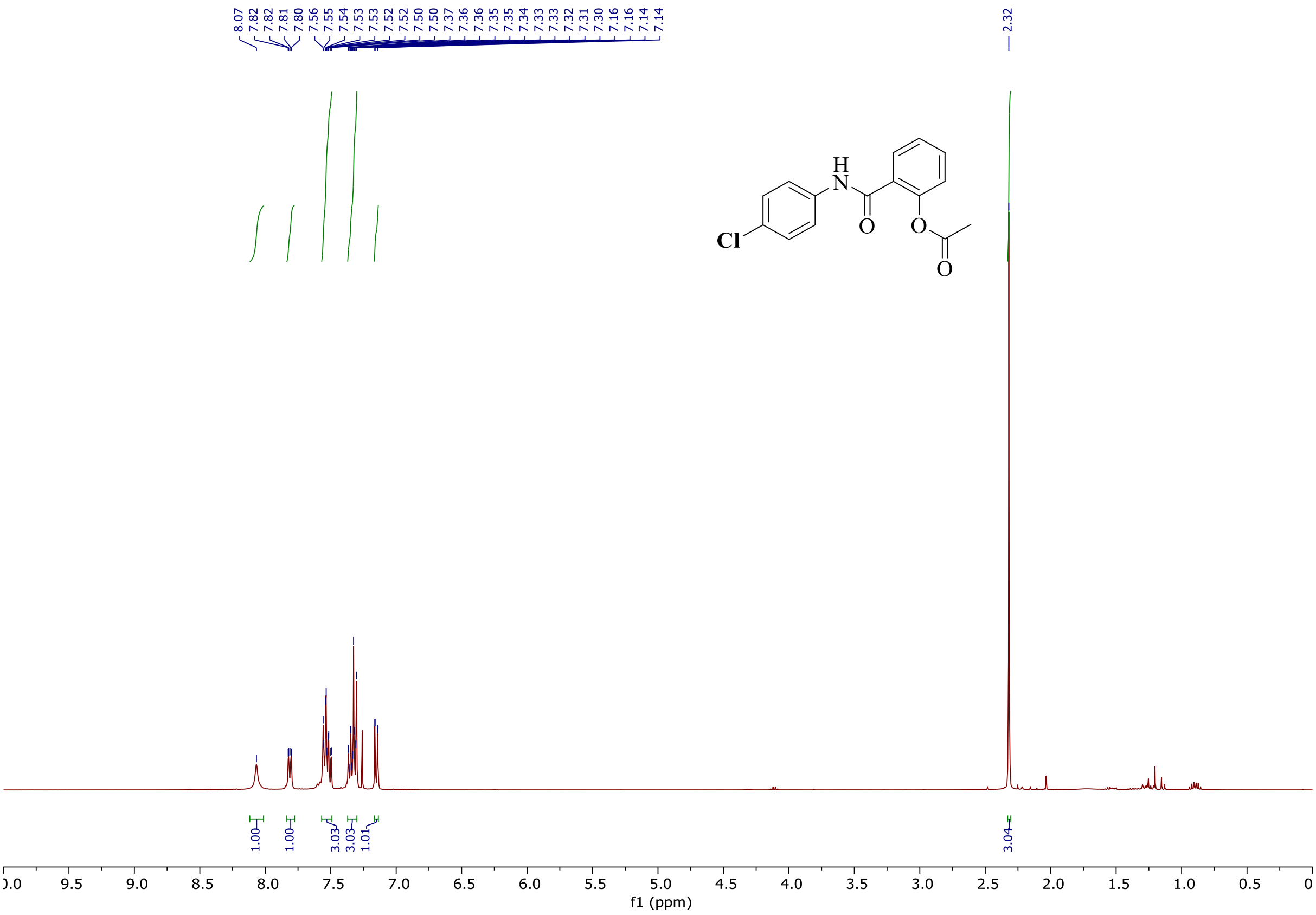

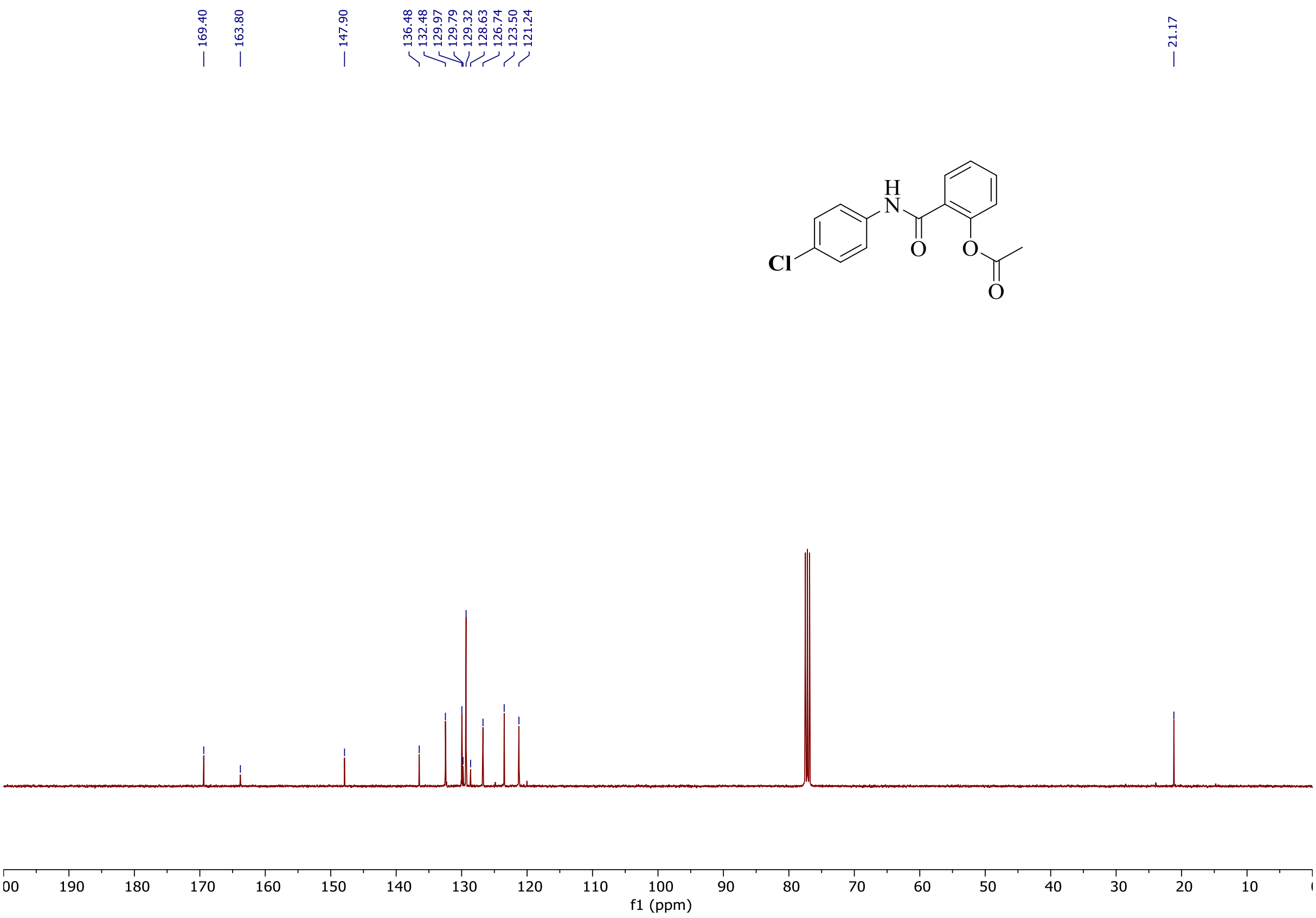

6-(3-((3r,5r,7r)-adamantan-1-yl)-4-methoxyphenyl)-N-(4-chlorophenyl)-2-naphthamide: (26)

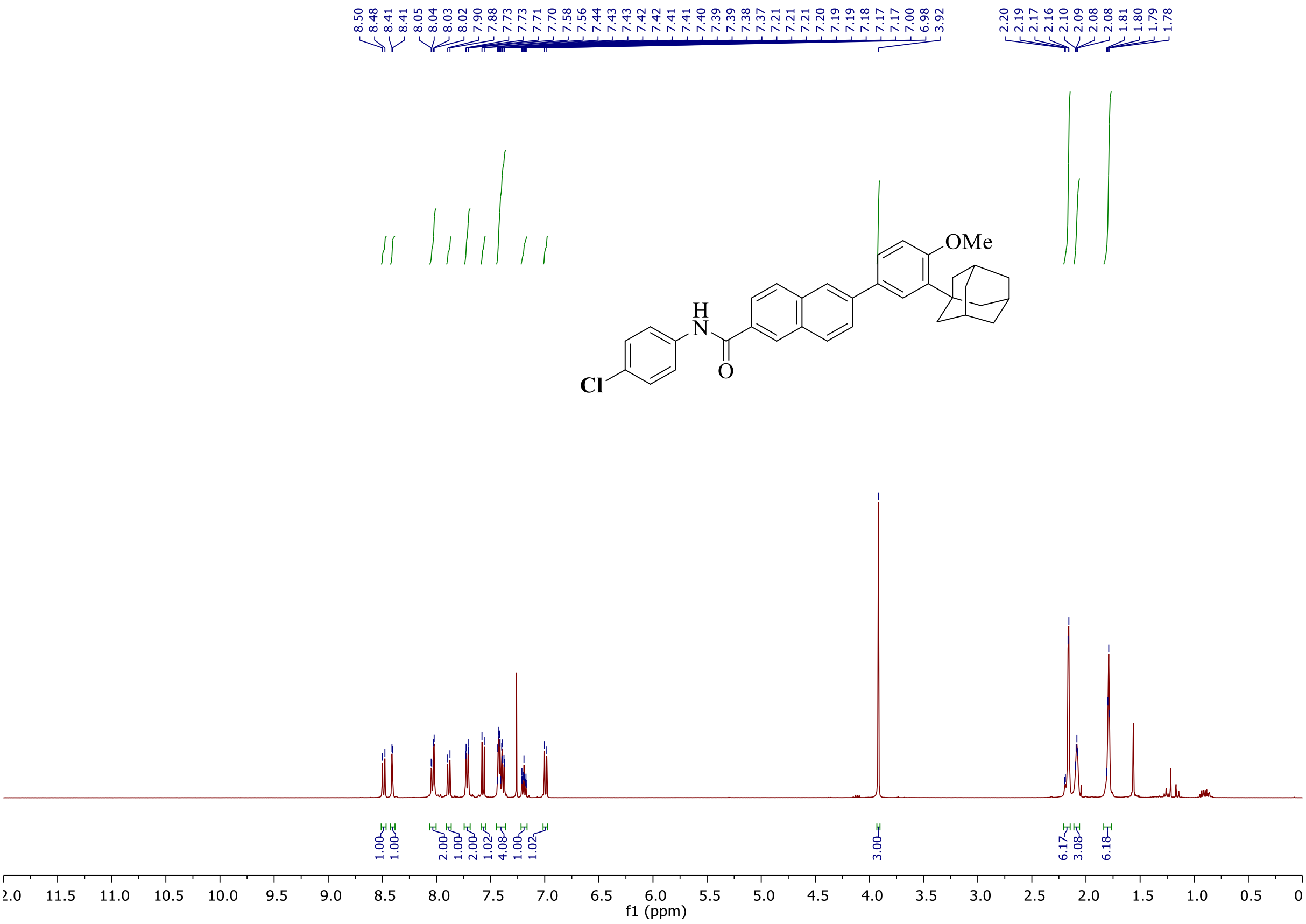

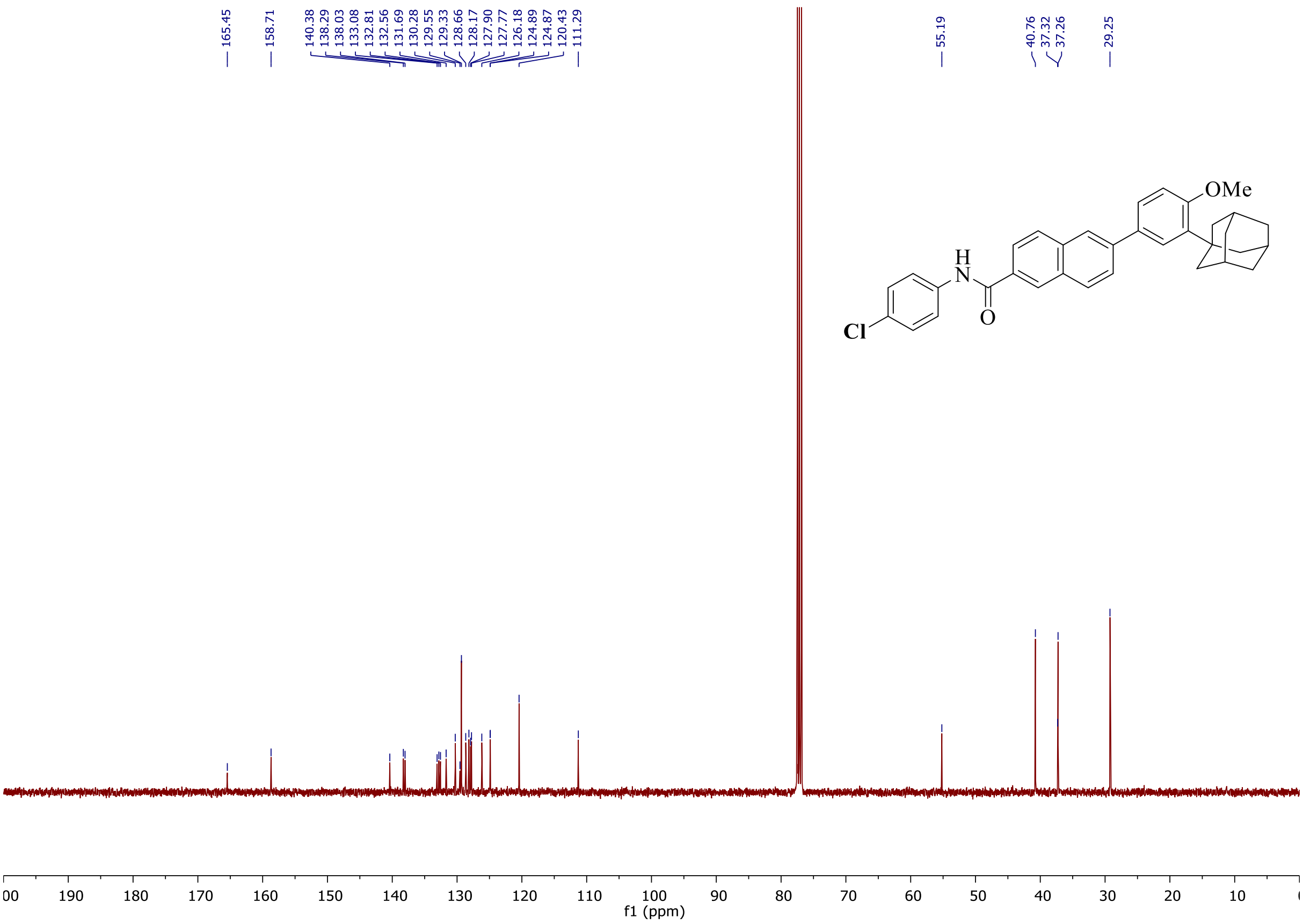

***N*-(4-chlorophenyl)-3-(cyclopropylmethoxy)-4-(difluoromethoxy)benzamide: (27)**

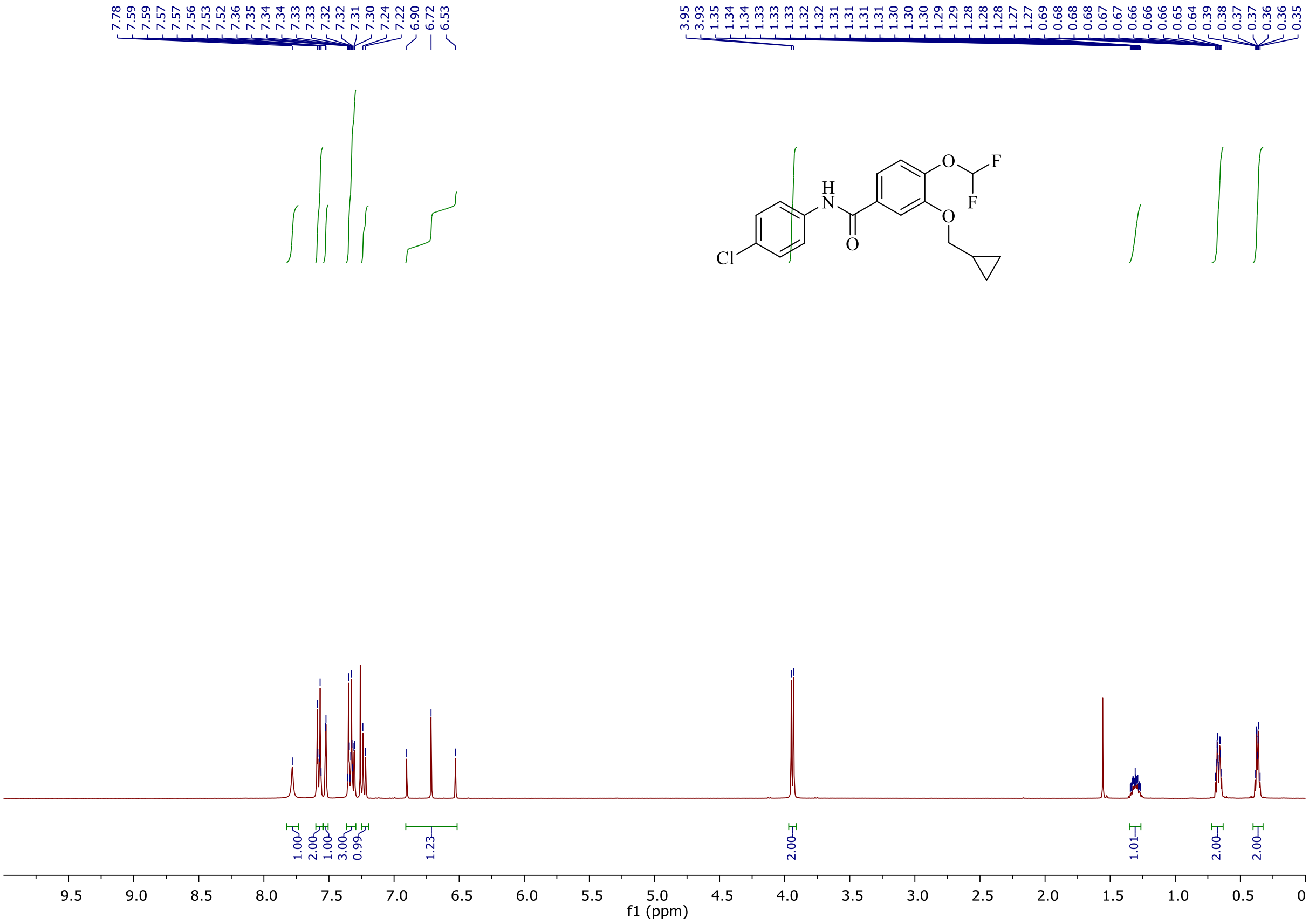

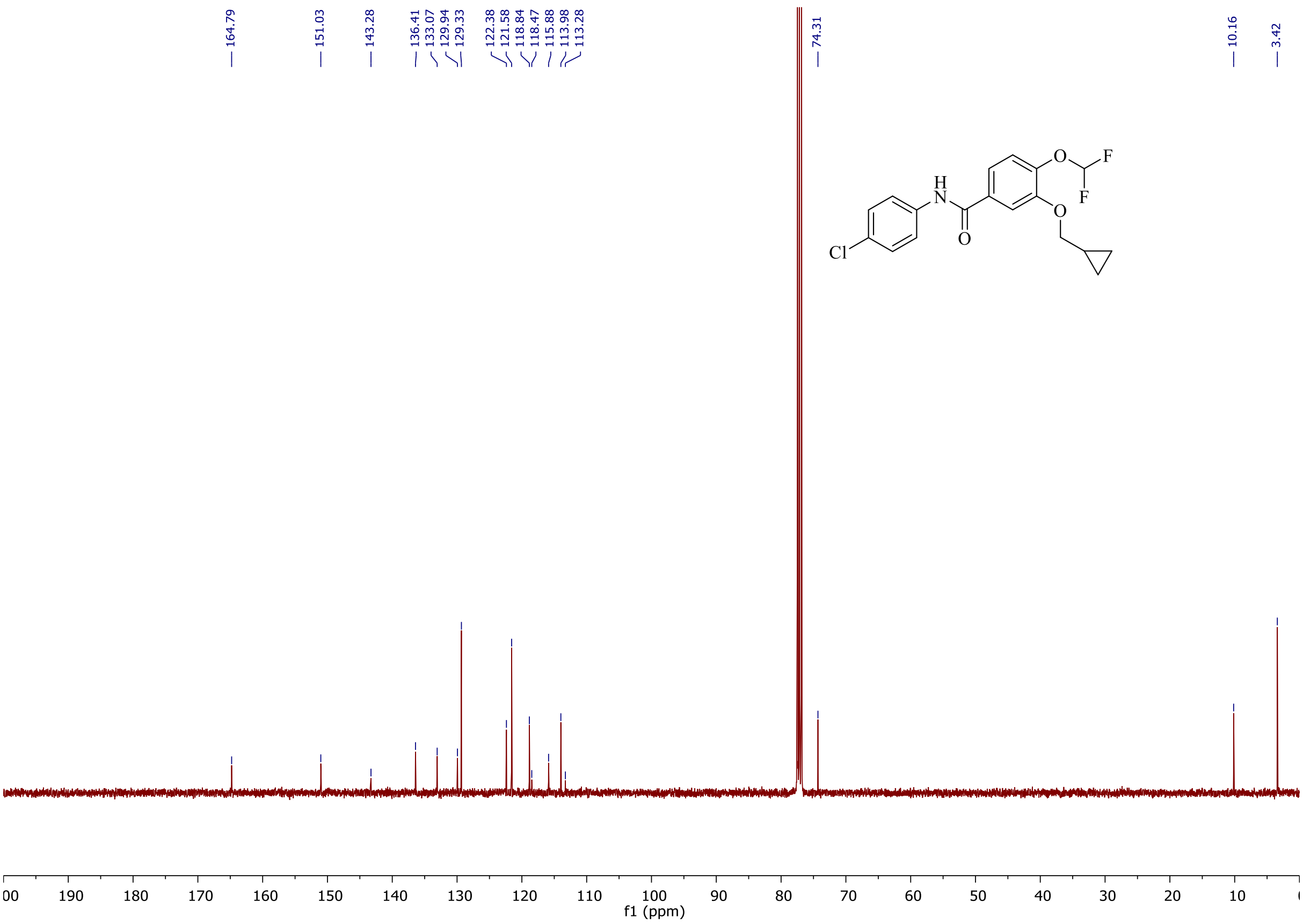

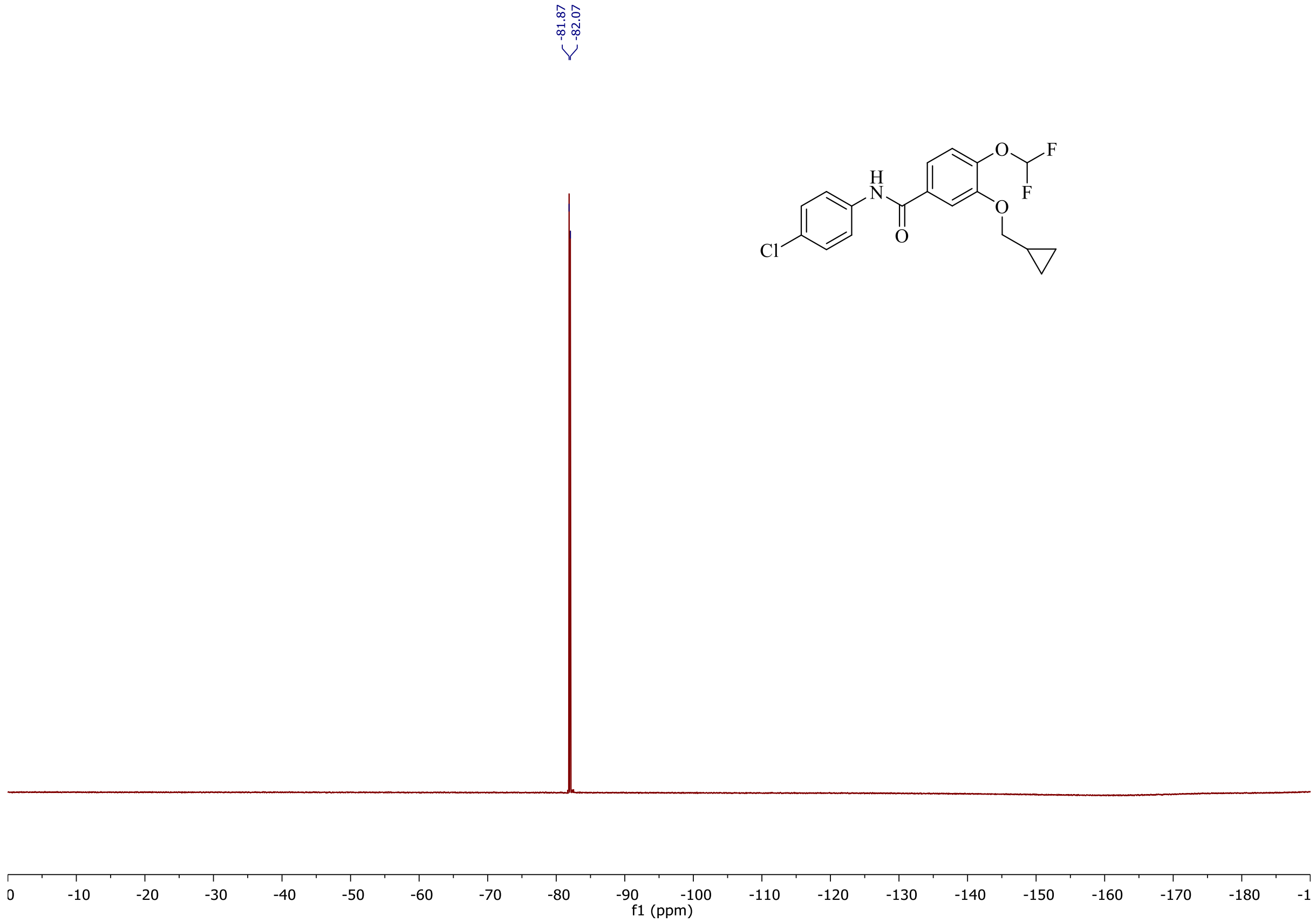
-81.87
-82.07
H
N
O
O
F
F
O
Cl
0
-10
-20
-30
-40
-50
-60
-70
-80
-90
-100
-110
-120
-130
-140
-150
-160
-170
-180
f1 (ppm)

5-(4-Chloro-2,5-dimethylphenoxy)-N-(4-chlorophenyl)-2,2-dimethylpentanamide: (28)

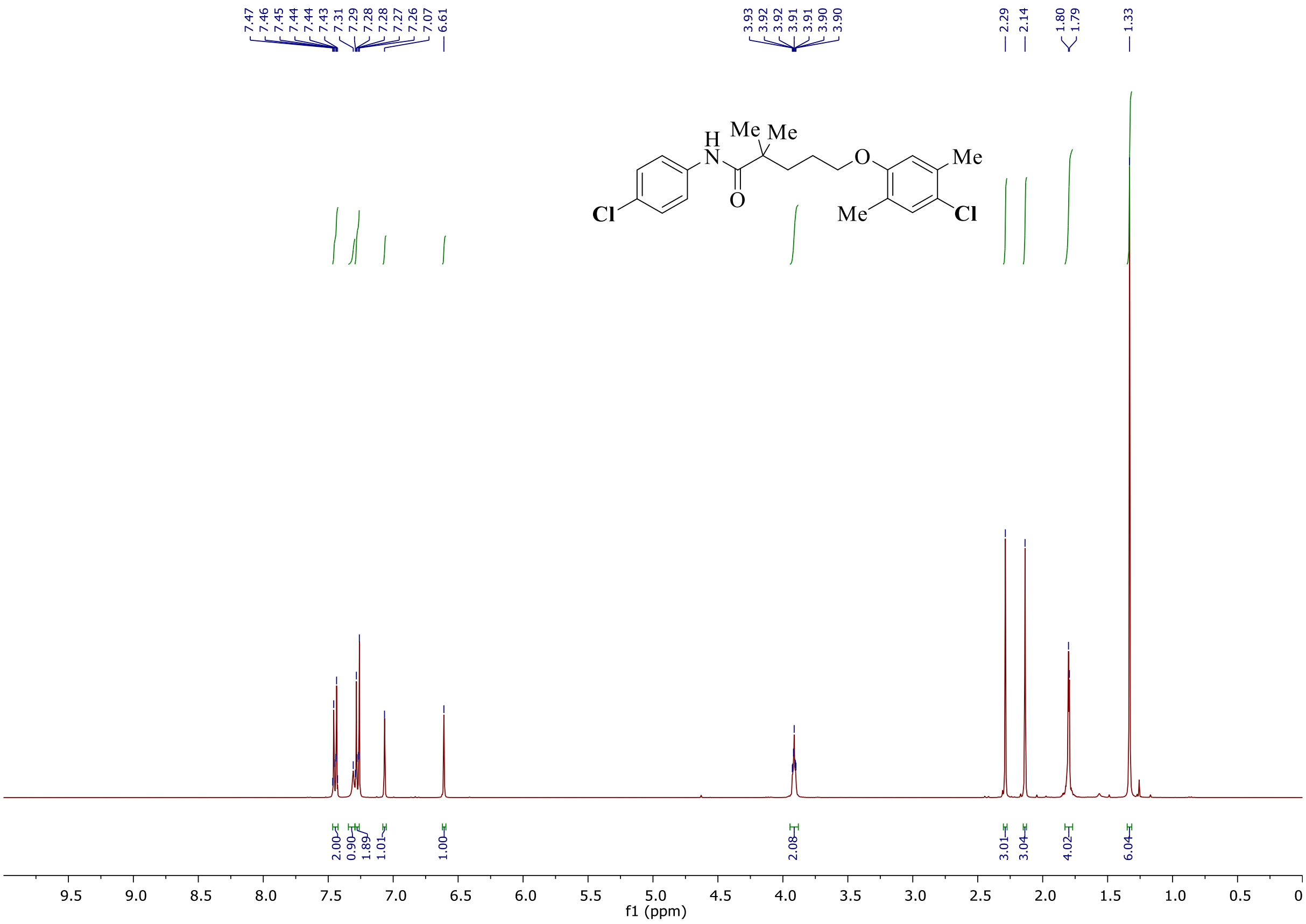

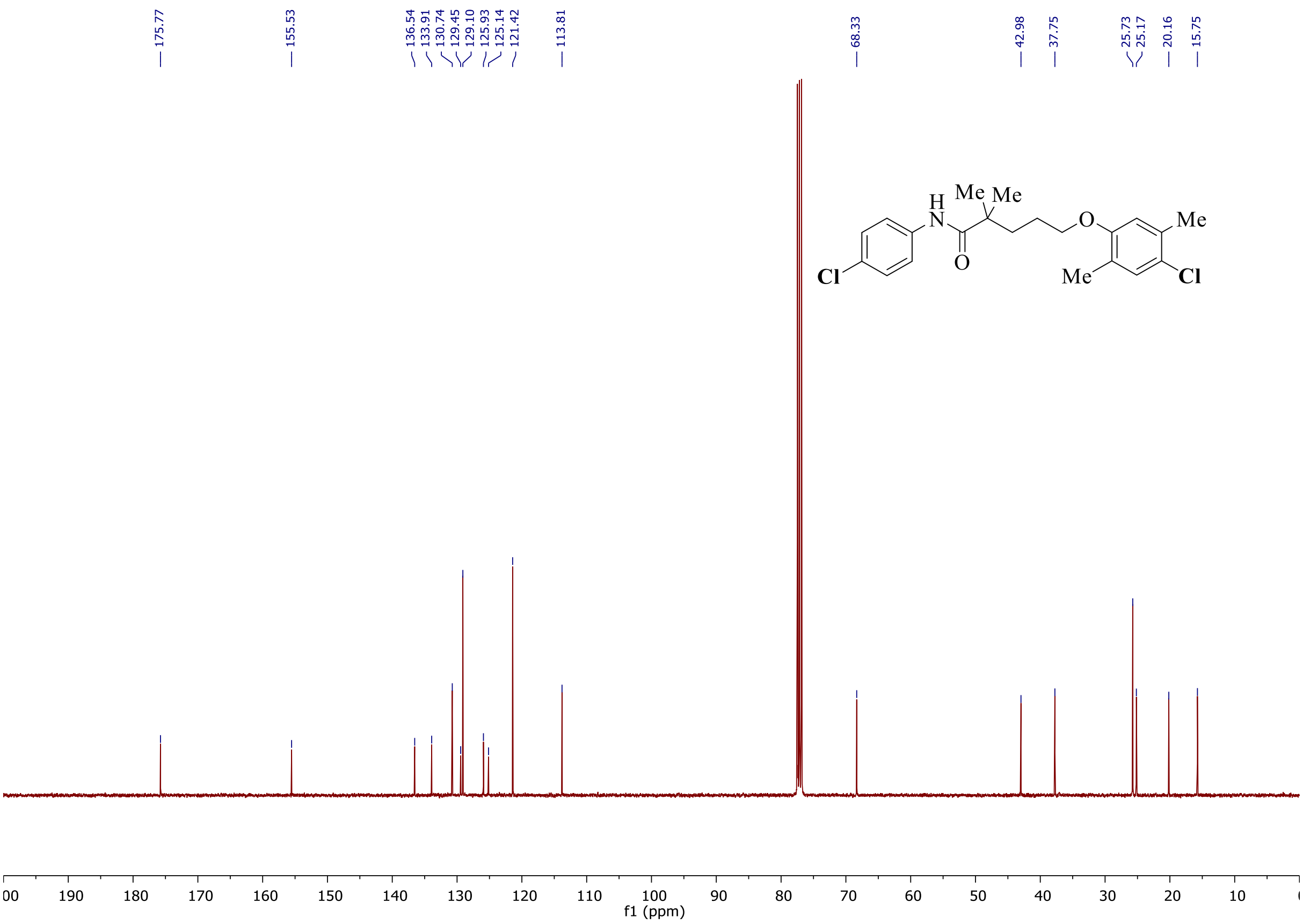

N-(2,6-dichloro-3,5-dimethoxyphenyl)-4-(N,N-dipropylsulfamoyl)benzamide: (29)

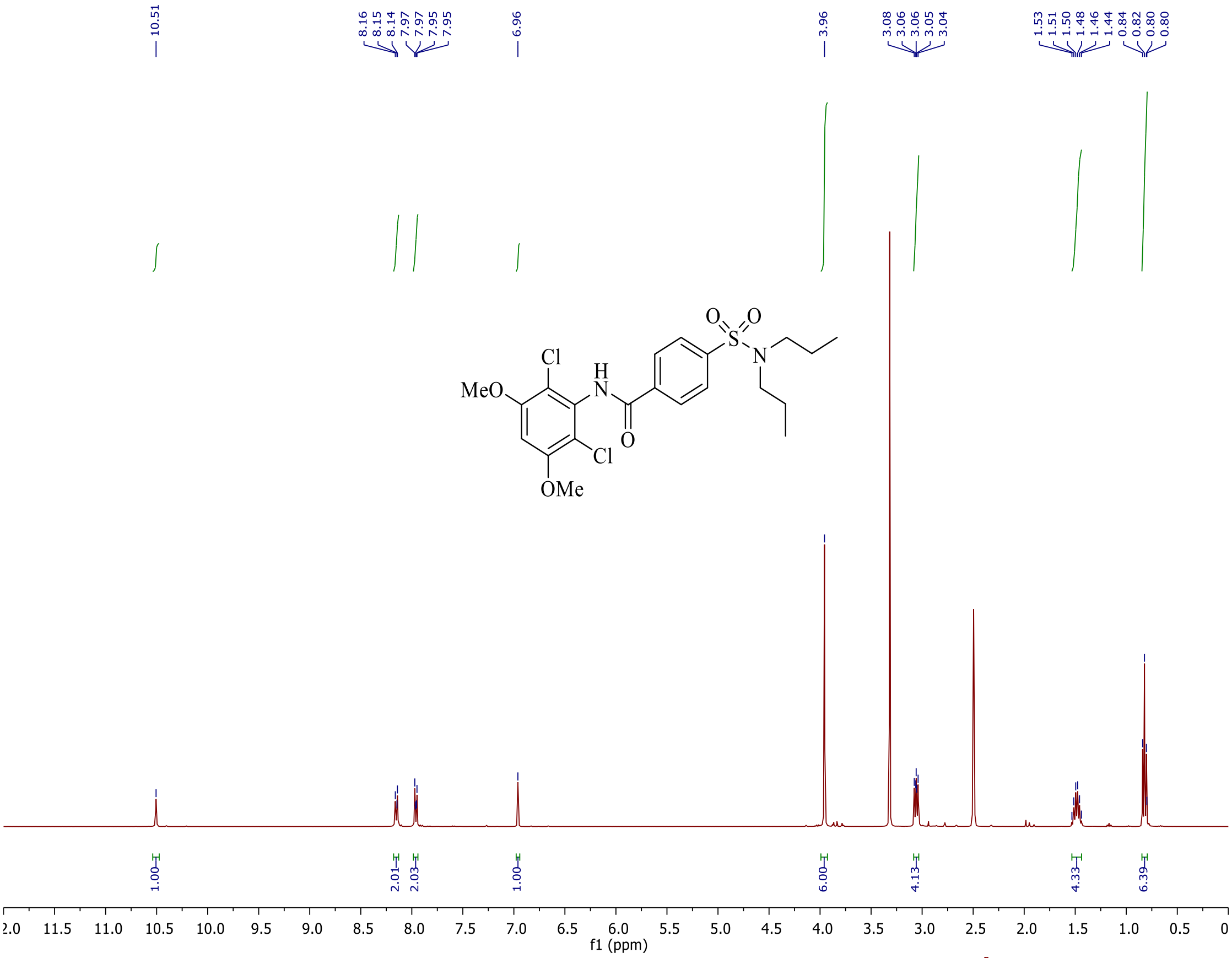

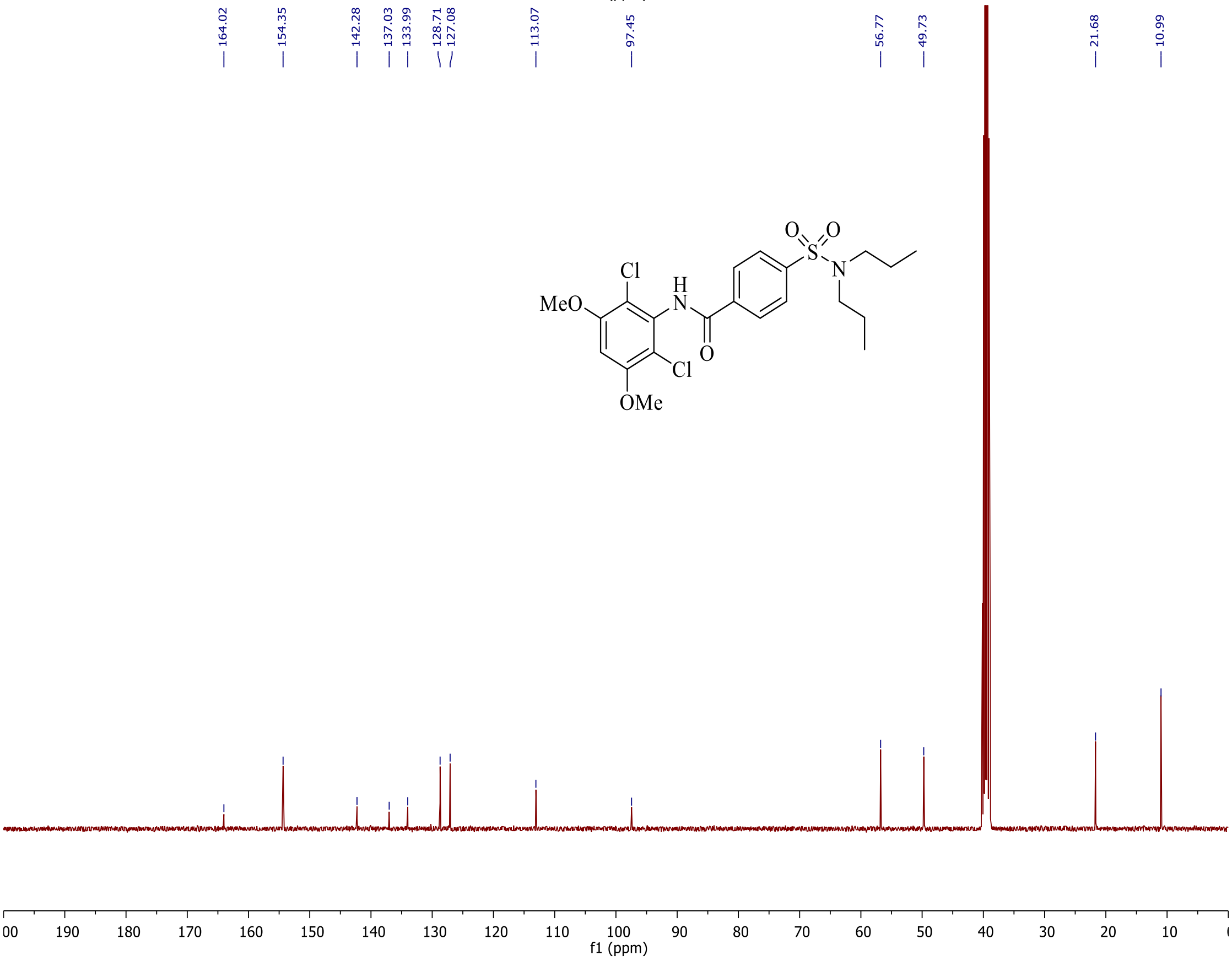

N-(1-bromonaphthalen-2-yl)-2-(2-fluoro-[1,1'-biphenyl]-4-yl)propanamide: (30)

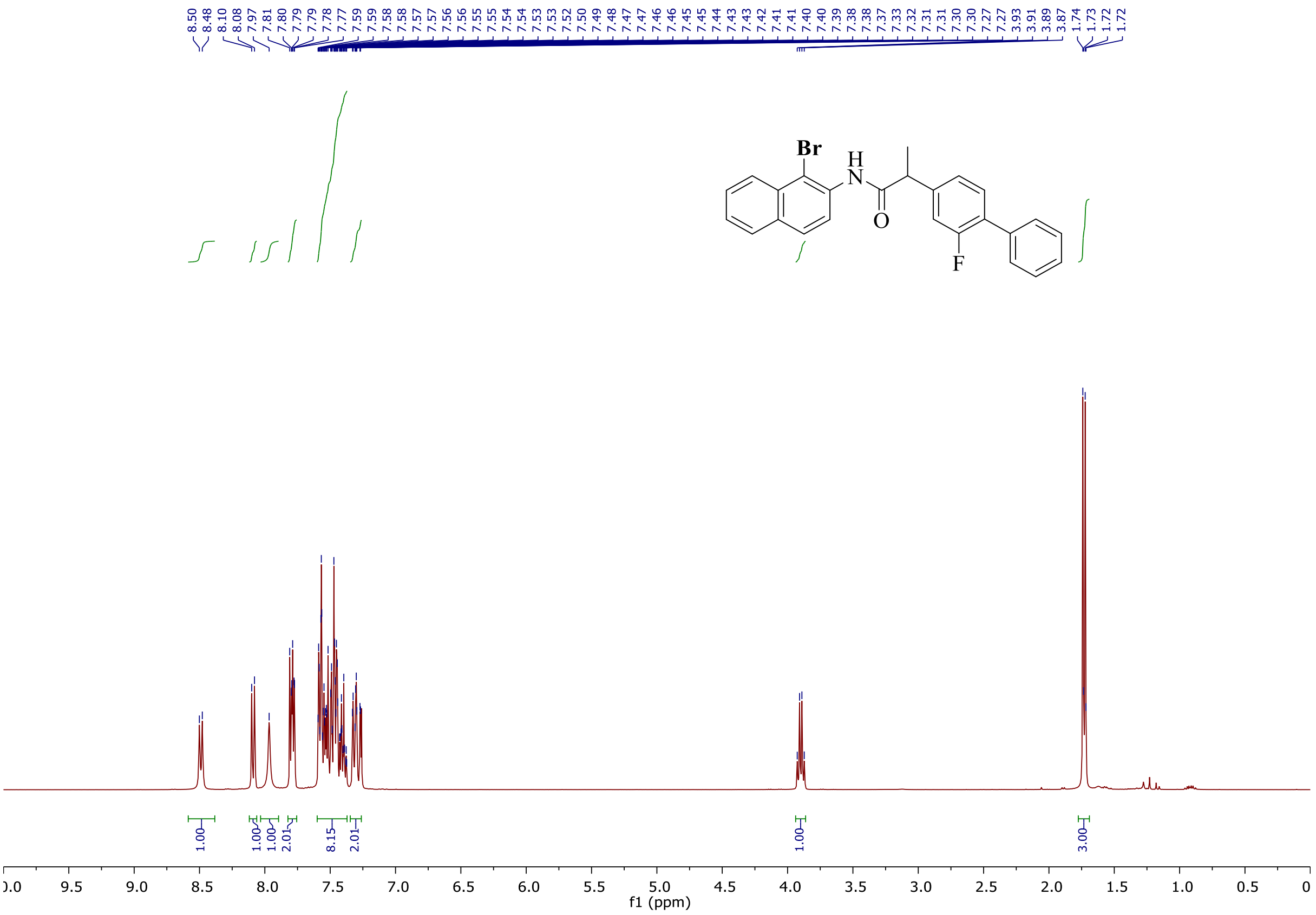

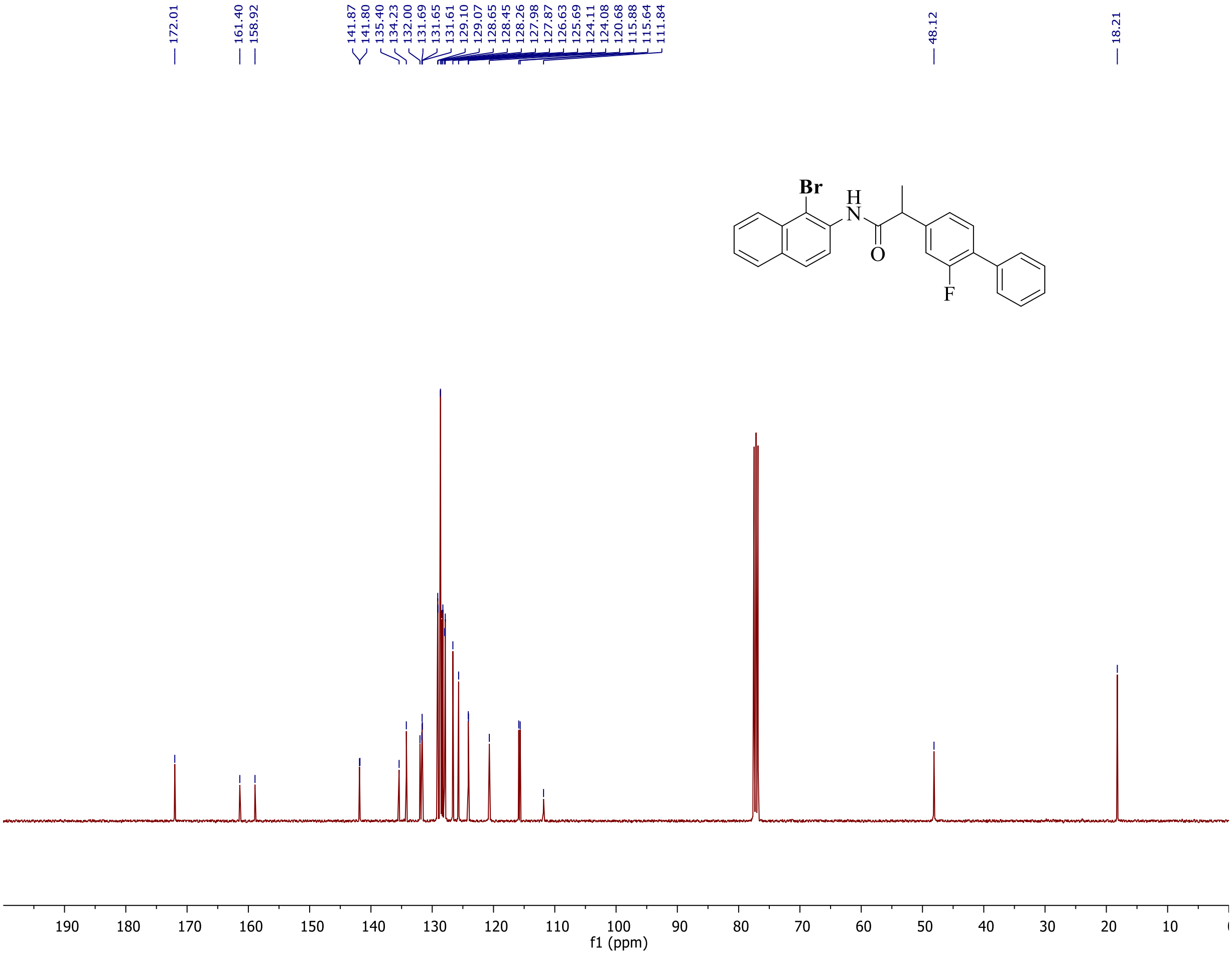

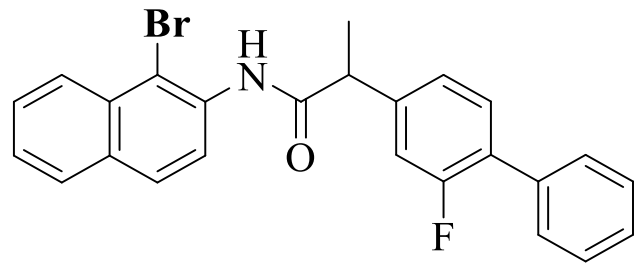
Br
H
N
O
F

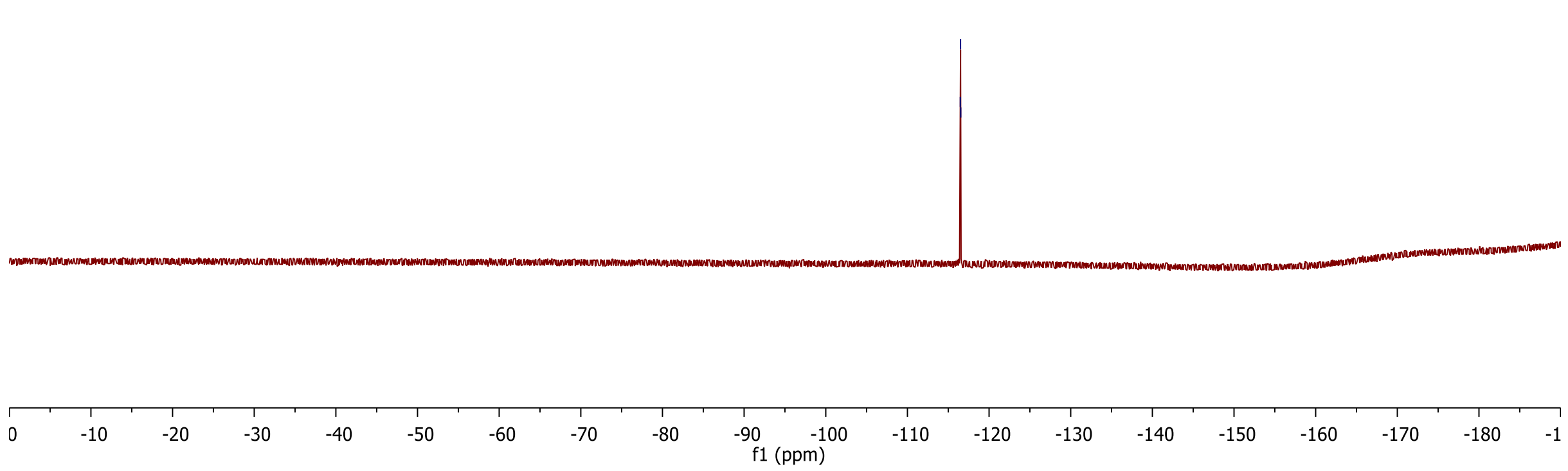
-116.48
-116.51
-116.53
0
-10
-20
-30
-40
-50
-60
-70
-80
-90
-100
-110
-120
-130
-140
-150
-160
-170
-180
f1 (ppm)

N-(4-bromophenyl)-3-(cyclopropylmethoxy)-4-(difluoromethoxy)benzamide: (31)

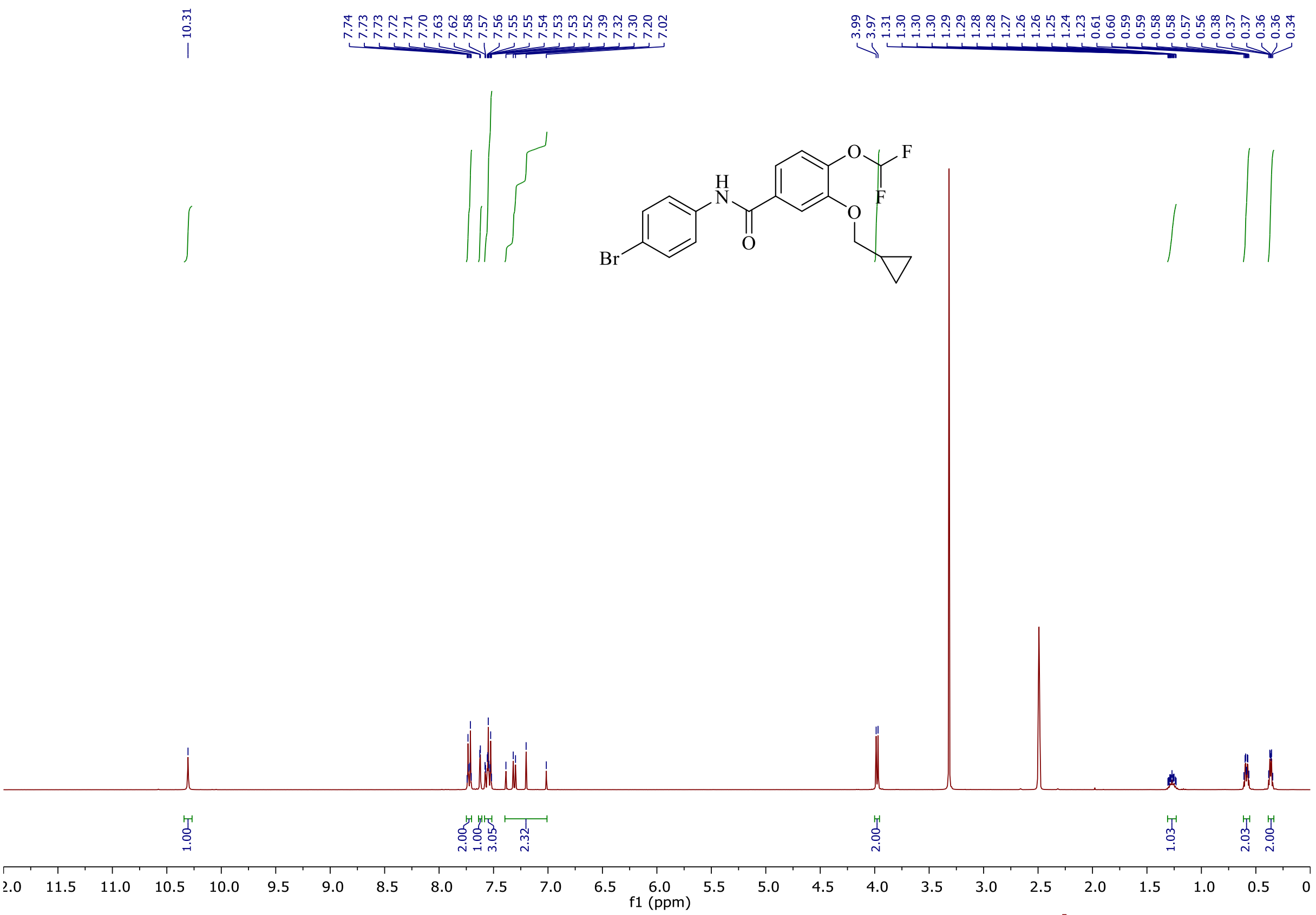

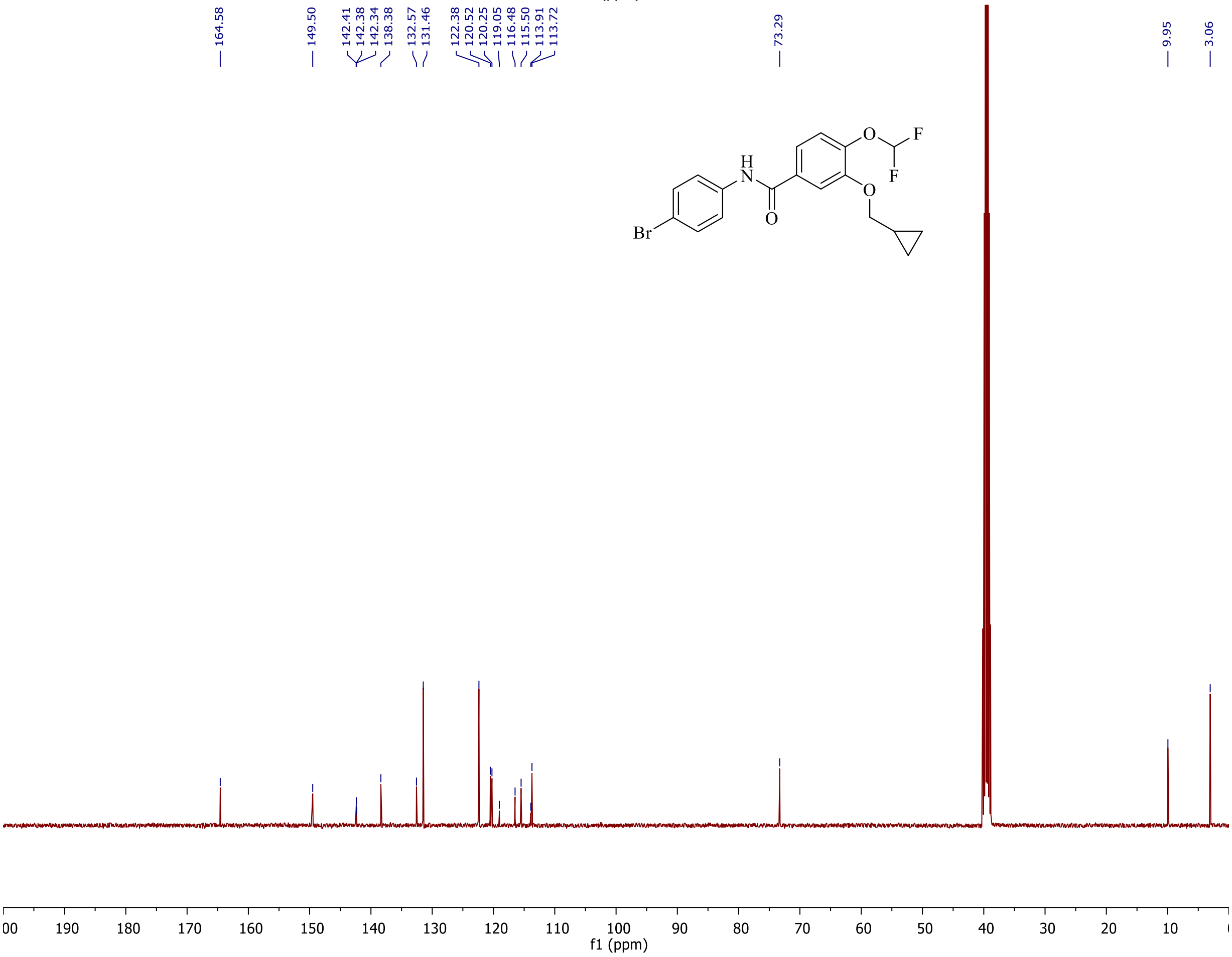

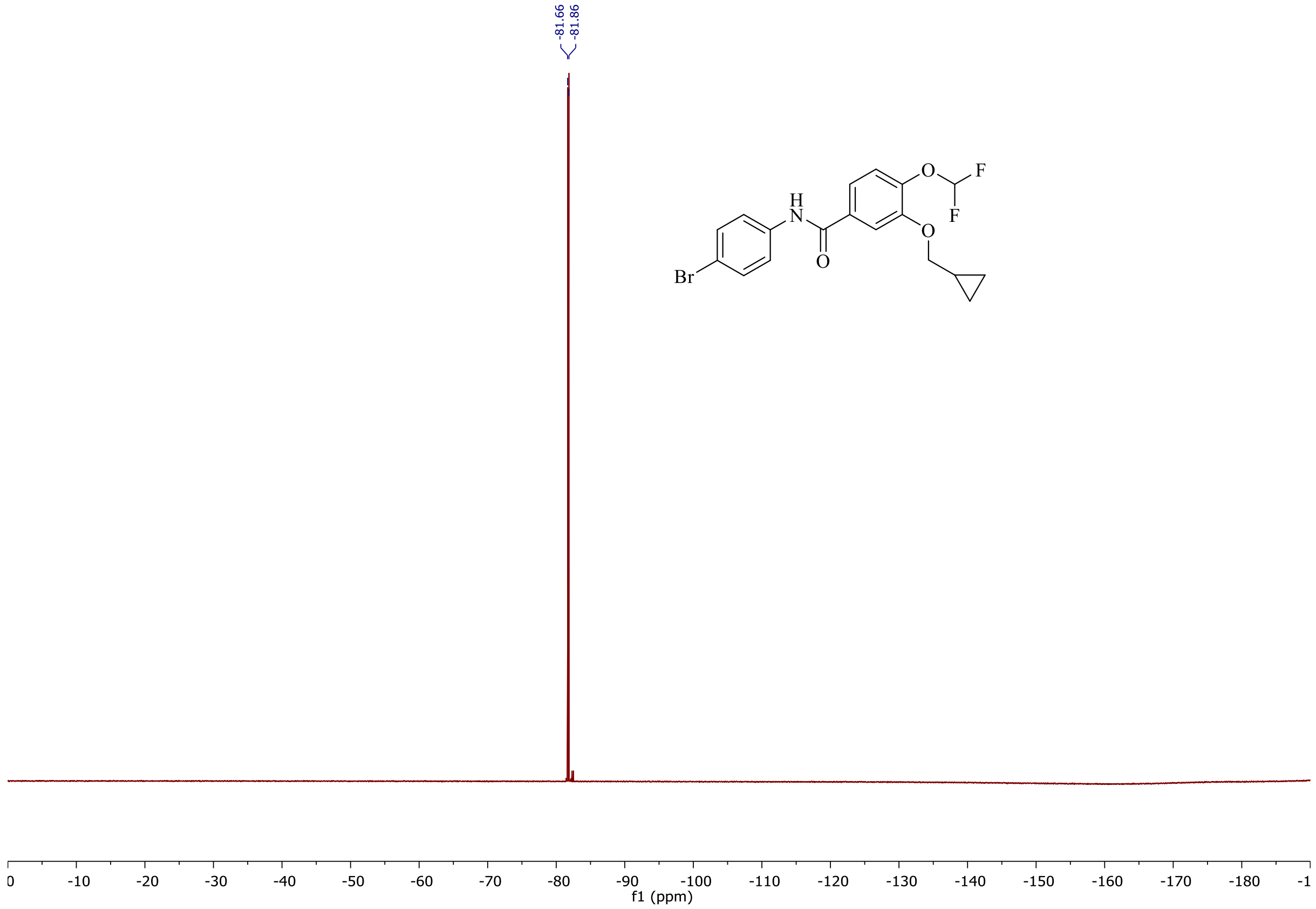
-81.66
-81.86
Br
H
N
O
O
F
F
O
0
-10
-20
-30
-40
-50
-60
-70
-80
-90
-100
-110
-120
-130
-140
-150
-160
-170
-180
-1
f1 (ppm)

N-(2-bromo-3,5-dimethoxyphenyl)-3-(cyclopropylmethoxy)-4-(difluoromethoxy)benzamide: (32)

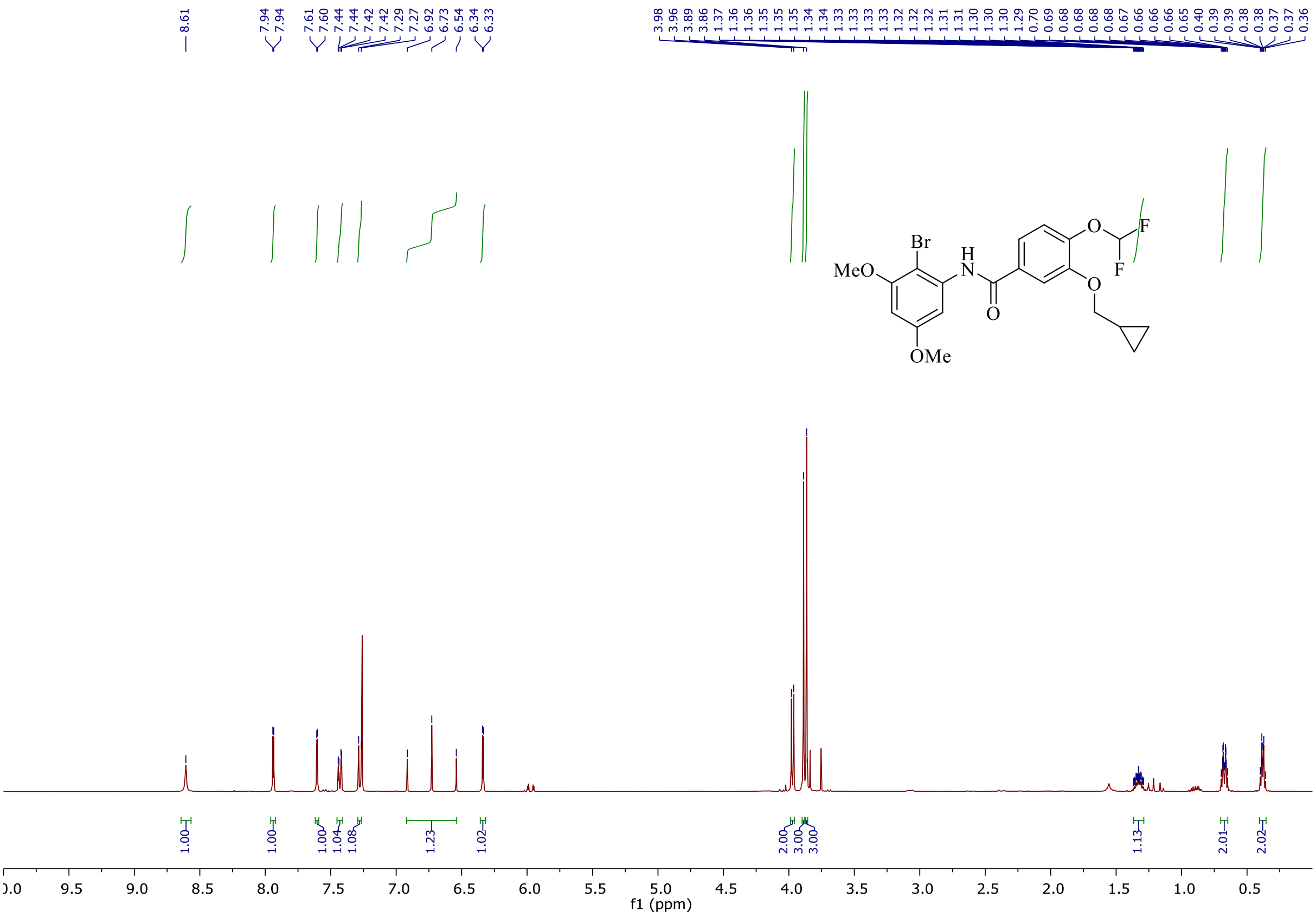

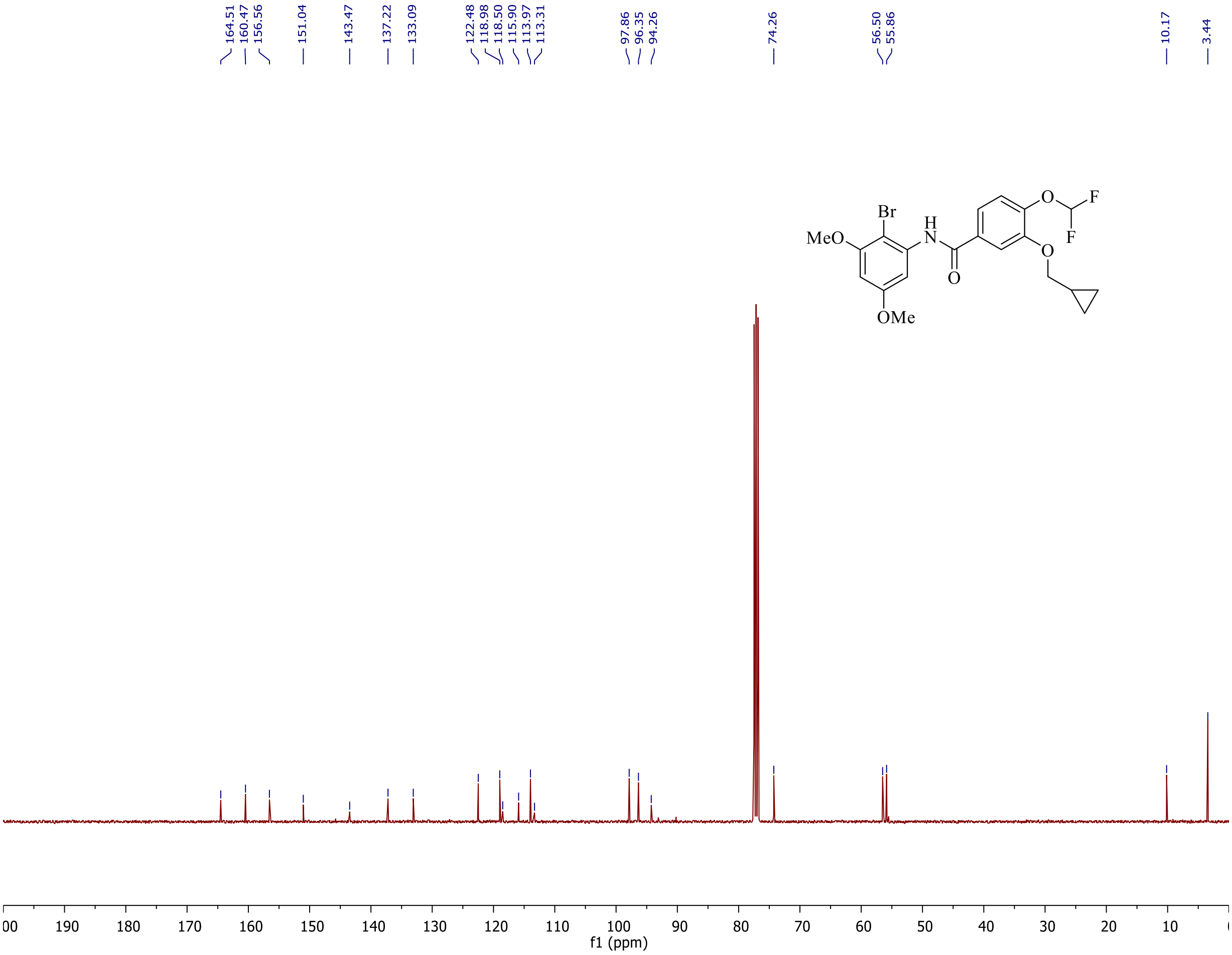

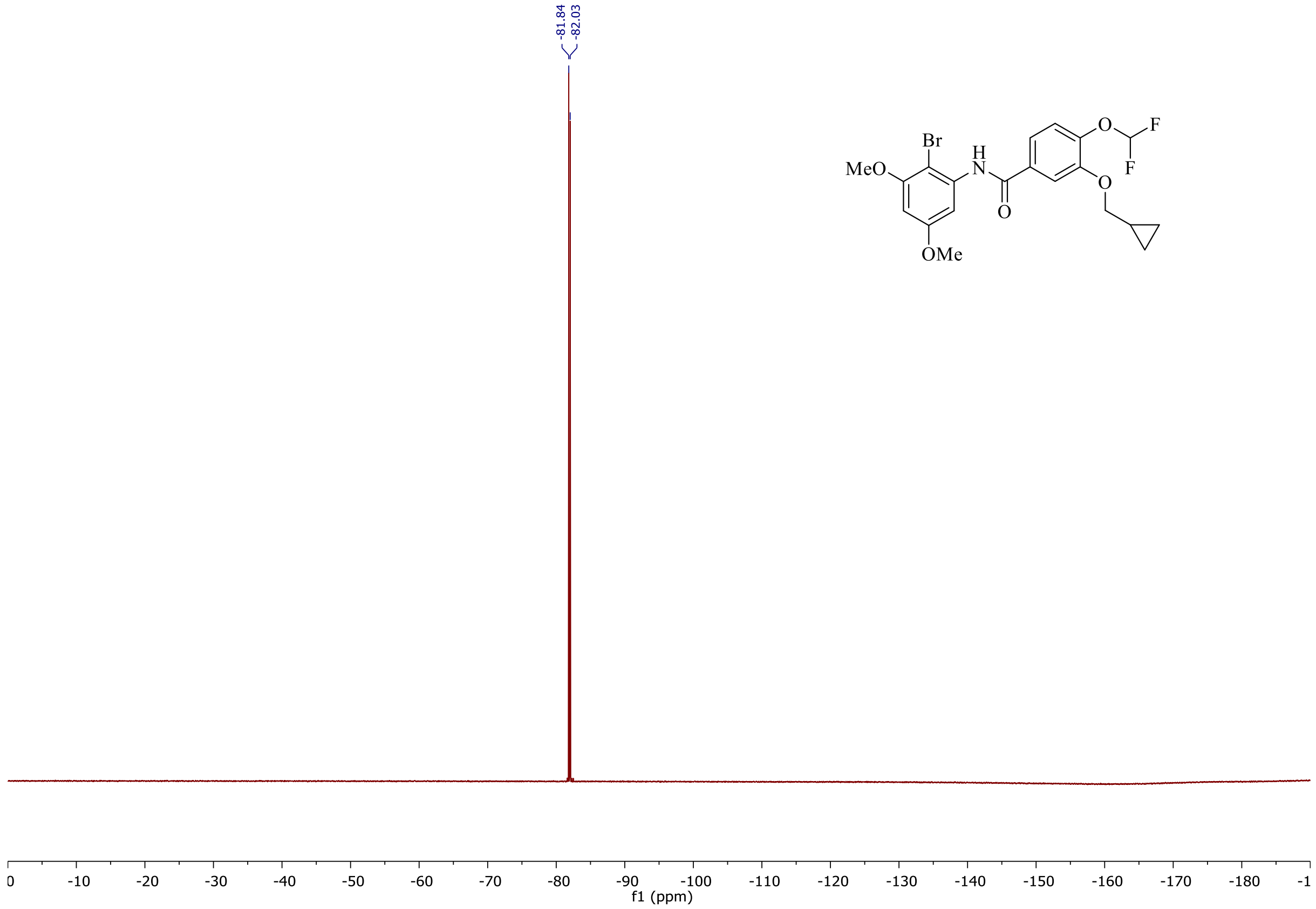
-81.84
-82.03
Br
H
N
MeO
O
OMe
O
F
F
O
0
-10
-20
-30
-40
-50
-60
-70
-80
-90
-100
-110
-120
-130
-140
-150
-160
-170
-180
f1 (ppm)
